# Epidemiological trends of urinary tract infections, urolithiasis and benign prostatic hyperplasia in 203 countries and territories from 1990 to 2019

**DOI:** 10.1186/s40779-021-00359-8

**Published:** 2021-12-09

**Authors:** Cong Zhu, Dan-Qi Wang, Hao Zi, Qiao Huang, Jia-Min Gu, Lu-Yao Li, Xing-Pei Guo, Fei Li, Cheng Fang, Xiao-Dong Li, Xian-Tao Zeng

**Affiliations:** 1 Department of Urology, Zhongnan Hospital of Wuhan University, Wuhan, 430071 Hubei China; 2 Center for Evidence-Based and Translational Medicine, Zhongnan Hospital of Wuhan University, Wuhan, 430071 Hubei China; 3 Department of Evidence-Based Medicine and Clinical Epidemiology, Second School of Clinical Medicine, Wuhan University, Wuhan, 430071 Hubei China; 4 Institutes of Evidence-Based Medicine and Knowledge Translation, Henan University, Kaifeng, 475000 Henan China; 5 Department of Urology, Huaihe Hospital of Henan University, Kaifeng, 475000 Henan China

**Keywords:** Urinary tract infections, Urolithiasis, Benign prostatic hyperplasia, Disease burden, Incidence, Mortality, Disability-adjusted life-years (DALYs)

## Abstract

**Background:**

Urinary tract infections (UTI), urolithiasis, and benign prostatic hyperplasia (BPH) are three of the most common nonmalignant conditions in urology. However, there is still a lack of comprehensive and updated epidemiological data. This study aimed to investigate the disease burden of UTI, urolithiasis, and BPH in 203 countries and territories from 1990 to 2019.

**Methods:**

Data were extracted from the Global Burden of Disease 2019, including incident cases, deaths, disability-adjusted life-years (DALYs) and corresponding age-standardized rate (ASR) from 1990 to 2019. Estimated annual percentage changes (EAPC) were calculated to evaluate the trends of ASR. The associations between disease burden and social development degrees were analyzed using a sociodemographic index (SDI).

**Results:**

Compared with 1990, the incident cases of UTI, urolithiasis, and BPH increased by 60.40%, 48.57%, and 105.70% in 2019, respectively. The age-standardized incidence rate (ASIR) of UTI increased (EAPC = 0.08), while urolithiasis (EAPC = − 0.83) and BPH (EAPC = − 0.03) decreased from 1990 to 2019. In 2019, the age-standardized mortality rate (ASMR) of UTI and urolithiasis were 3.13/100,000 and 0.17/100,000, respectively. BPH had the largest increase (110.56%) in DALYs in the past three decades, followed by UTI (68.89%) and urolithiasis (16.95%). The burden of UTI was mainly concentrated in South Asia and Tropical Latin America, while the burden of urolithiasis and BPH was recorded in Asia and Eastern Europe. Moreover, the ASIR and SDI of urolithiasis in high-SDI regions from 1990 to 2019 were negatively correlated, while the opposite trend was seen in low-SDI regions. In 2019, the ASIR of UTI in females was 3.59 times that of males, while the ASIR of urolithiasis in males was 1.96 times higher than that in females. The incidence was highest in the 30–34, 55–59, and 65–69 age groups among the UTI, urolithiasis, and BPH groups, respectively.

**Conclusion:**

Over the past three decades, the disease burden has increased for UTI but decreased for urolithiasis and BPH. The allocation of medical resources should be based more on the epidemiological characteristics and geographical distribution of diseases.

**Supplementary Information:**

The online version contains supplementary material available at 10.1186/s40779-021-00359-8.

## Background

Urinary tract infections (UTI), urolithiasis and benign prostatic hyperplasia (BPH) are three of the most common nonmalignant conditions in urology. Compared with the shortened survival caused by cancer, these urologic benign diseases affect individuals by impairing their quality of life to a great extent. In the United States, there were approximately 10.50 million ambulatory visits and 2–3 million emergency department visits for UTI in 2007, and $3.50 billion were spent in 2015 [1]. A systematic review and meta-analysis containing 31 studies reported that the lifetime prevalence of BPH was 26.20% (95% CI 22.80%–29.60%) [2]. The prevalence of urolithiasis varies in different regions, and the prevalence of urolithiasis was 7.00% in Australia in 2000, 5.06% in Spain in 2007, 8.80% in America in 2010 and 6.50% in China in 2015 [3–5]. The burden of urolithiasis has increased drastically over the last half century, and the annual estimated financial burden was $5.30 billion in 2014 [6, 7]. Over the past decades, the spectrum of diseases has changed with the aging population, socioeconomic development and advances in disease prevention and control. However, there is still a lack of comprehensive and updated epidemiological data on UTI, urolithiasis and BPH.

The Global Burden of Disease Study 2019 (GBD 2019) is a critical resource for informed policy-making and provides a tool to quantify health loss from hundreds of diseases, injuries, and risk factors. Regularly updated epidemiological data from the GBD database could help policymakers understand health trends over a period of time at the global, regional, and national levels. In this study, we used data from GBD 2019 to reveal the epidemiological trends of UTI, urolithiasis and BPH over the past 30 years according to global, regional, national, sociodemographic index (SDI), sex, and age.

## Methods

### Data sources

This study used data from the GBD 2019, which comprehensively analyzed the incidence, mortality, disability-adjusted life-years (DALYs) and age-standardized rate (ASR) of 369 diseases and injuries of different sexes and ages in 203 countries and territories around the world. The incidence, death, and DALYs as well as the corresponding ASR and 95% uncertainty interval (UI) of UTI, urolithiasis and BPH were obtained from the Global Health Data Exchange GBD Results Tool (http://ghdx.healthdata.org/). The SDI is a composite indicator of development status strongly correlated with health outcomes. SDI ranged from 0 to 1, where 0 represents the minimum level of development, and 1 represents the maximum level of development. The 203 countries and territories were categorized according to the SDI quintile into 5 groups: low-SDI, low-middle-SDI, middle-SDI, high-middle-SDI, and high-SDI regions. Age was classified into fifteen subgroups: 0–14, 15–19, 20–24, 25–29, 30–34, 35–39, 40–44, 45–49, 50–54, 55–59, 60–64, 65–69, 70–74, 75–79, and over 80 years.

The incidence of these three urologic benign diseases was estimated from the data of hospital discharges and claims, with a systematic literature review additionally used for urolithiasis. Then, the DisMod­MR 2.1 model, a Bayesian meta­regression tool, was used to produce estimates by age, sex, year, and country. Disability weight (DW) is the severity of health loss or the severity of nonfatal disability and is an important parameter reflecting the burden of disease. DW ranges from 0 to 1, where health is 0 and death is 1, and the more severe the disability, the closer it is to 1. Years lived with disability (YLD) is equal to the number of cases multiplied by duration until remission or death multiplied by DW. The mortality of UTI and urolithiasis was estimated from vital registration data and verbal autopsy data. The standard CODEm model with location-level covariates was used to model deaths, and then the results were adjusted using CodCorrect to reach final years of life lost (YLL). DALYs are obtained by adding YLD and YLL. BPH is a chronic, nonfatal disease; thus, GBD 2019 assumed that there was no excess mortality related to BPH. The DALYs of BPH are equal to YLD. Detailed data sources and model methods were reported in GBD 2019 [8].

### Definition of the three urologic benign diseases

In GBD 2019, UTI was defined as a kidney infection that can lead to systemic symptoms such as fever and weakness and can cause discomfort and difficulty with daily activities. Urolithiasis is an acute and usually symptomatic episode of urolithiasis, defined as stone formation located anywhere along the genitourinary tract. BPH is defined as a benign proliferation of prostatic tissue, often leading to symptoms such as urinary retention, bladder outlet obstruction, or urinary tract infection. The associated International Classification of Diseases (ICD) codes include N10, N10.0, N10.9, N11, N11.0, N11.1, N11.8, N11.9, N12, N12.0, N12.9, N13.6, N15, N15.1, N15.8, N15.9, N16, N16.0-N16.5, N16.8, N30, N30.0-N30.3, N30.8-N30.9, N34, N34.0-N34-3, and N39.0 for UTI; N20, N20.0, N20.1, N20.2, N20.9, N21, N21.1, N21.8, N21.9, N22, N22.0, N22.8, N23, and N23.0 for urolithiasis; and N40, N40.0, N40.1, N40.2, N40.3, and N40.9 for BPH [8].

### Statistical analyses

The ASR (per 100,000 population), which can reflect the differences between different groups composed of different ages or age compositions changing over time more accurately, was calculated on the basis of the formula: ASR = $\frac{{\mathop \sum \nolimits_{i = 1}^{A} a_{i} w_{i} }}{{\mathop \sum \nolimits_{i = 1}^{A} w_{i} }}$ × 100,000 (a_i_, where i denotes the *i*th age class, and the number of persons (or weight w_i_) in the same age subgroup i of the selected reference standard population). To comprehensively estimate the trends of ASR over time, the estimated annual percentage changes (EAPC) were calculated using a generalized linear model considering a Gaussian distribution for the ASR. With the EAPC methodology, an assumption of linearity on the log scale of ASR, which is equivalent to a constant change assumption, was made [9]. Thus, Y = α + βX + ε, where Y refers to ln (ASR), X represents the calendar year, and ε represents the error term. Based on this formula, β determines the positive or negative trends in ASR. The EAPC and its 95% confidence interval were calculated from the linear regression model (EAPC = 100 × [exp (β) − 1]). In this case, the outcome should be log-transformed ASR where the linear assumption holds. It is shown that when EAPC and the lower boundary of the 95% CI are positive, then ASR is in an upward trend. Conversely, when EAPC and the upper boundary of the 95% CI are negative, the ASR shows a descending trend. All statistical analyses were performed using R software (Version 3.6.1).

## Results

### Global incidence, mortality, and DALYs

**Fig. 1:**
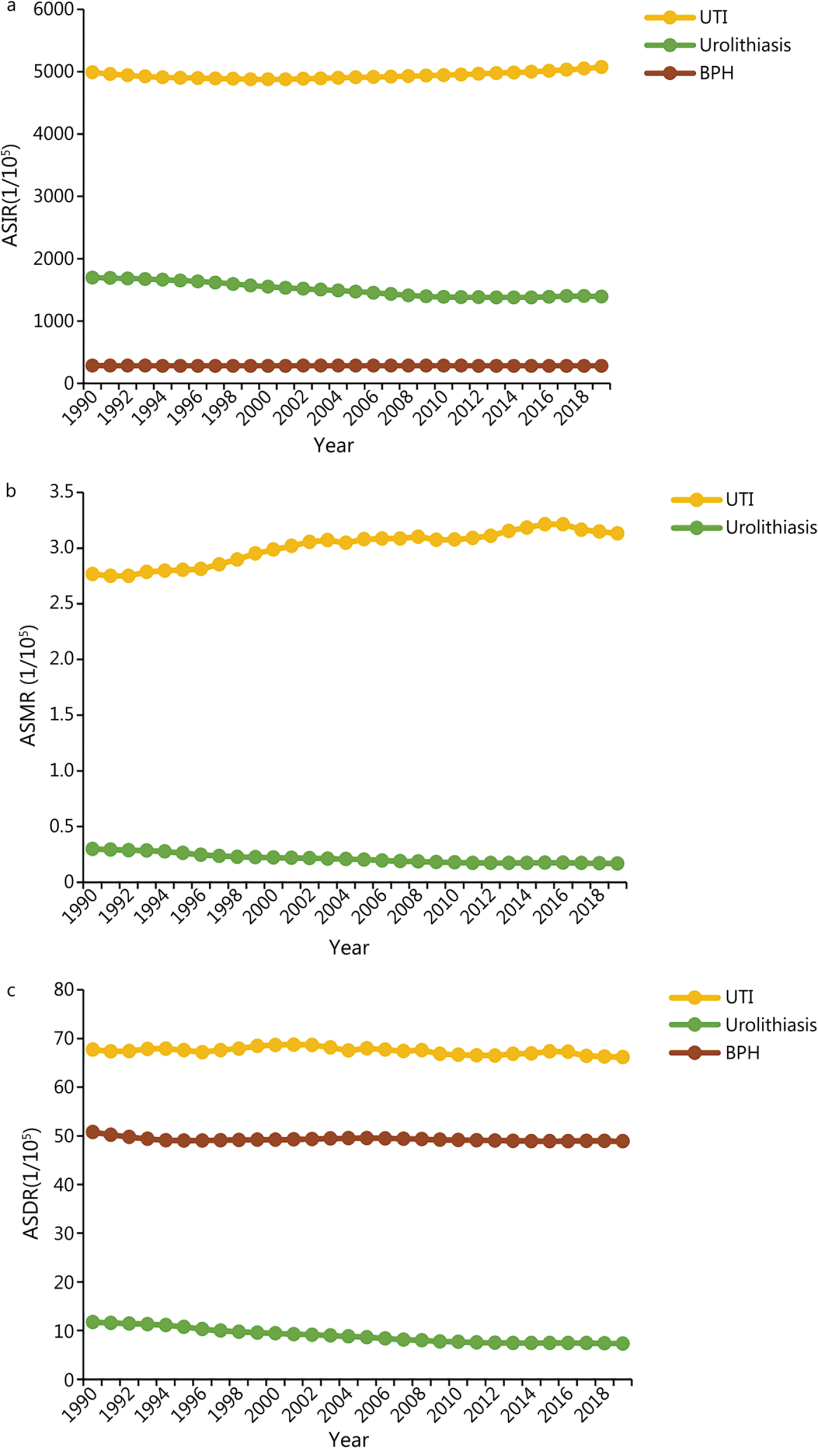
Global trends of the ASIR, ASMR, and ASDR of the three urologic benign diseases from 1990 to 2019. **a** ASIR. **b** ASMR. **c** ASDR. ASIR age-standardized incidence rate, ASMR age-standardized mortality rate, ASDR age-standardized DALY rate, BPH benign prostatic hyperplasia, UTI urinary tract infections

In 2019, the incident cases of UTI, urolithiasis, and BPH were 4046.12 × 10^5^ (95% UI 3594.25 to 4465.48), 1155.52 × 10^5^ (95% UI 930.45 to 1401.8), and 112.65 × 10^5^ (95% UI 87.9 to 144.55), respectively. Compared with 1990, the incident cases of UTI, urolithiasis, and BPH increased by 60.40%, 48.57%, and 105.70%, respectively. The age-standardized incidence rate (ASIR) of UTI (5075.89/100,000, 95% UI 4516.65/100,000 to 5594.10/100,000) was 3.64 times higher than that of urolithiasis (1394.03/100,000, 95% UI 1126.40/100,000 to 1688.16/100,000) and 18.10 times higher than that of BPH (280.40/100,000, 95% UI 219.62 to 360.32) in 2019 (Table 1). In addition, the ASIR of urolithiasis and BPH showed a decreasing trend with EAPCs of –0.83 (95% CI –0.92 to –0.74) and –0.03 (95% CI –0.05 to –0.01), while the ASIR of UTI showed an increasing trend (EAPC: 0.08, 95% CI 0.04 to 0.11) (Fig. 1a). From 1990 to 2019, the incident cases of these three diseases increased, while the ASIR of urolithiasis and BPH decreased.

**Table 1:** Global incidence, mortality, and DALYs of three urologic benign diseases from 1990 to 2019

	Urinary tract infections			Urolithiasis			Benign prostatic hyperplasia
	Both	Male	Female	Both	Male	Female	Male
*1990*^△^							
Incident case (× 10^5^)	2522.46 (2233.14 to 2792.97)	488.47 (437.22 to 536.93)	2033.99 (1795.48 to 2259.36)	777.76 (622.39 to 951.27)	527.82 (421.54 to 644.73)	249.94 (199.72 to 305.78)	54.76 (42.00 to 71.16)
Deaths (× 10^3^)	98.59 (89.03 to 106.32)	46.79 (39.61 to 52.73)	51.80 (46.42 to 56.17)	11.34 (7.28 to 13.78)	6.15 (3.08 to 8.03)	5.18 (3.51 to 6.12)	
DALYs (× 10^3^)	3079.89 (2651.63 to 3381.62)	1471.72 (1224.73 to 1656.34)	1608.17 (1362.18 to 1807.36)	516.73 (374.13 to 635.72)	308.57 (207.19 to 392.88)	208.16 (152.47 to 249.07)	884.19 (526.63 to 1337.22)
ASIR	4989.87 (4434.39 to 5499.05)	1984.80 (1784.33 to 2175.01)	4989.87 (4434.39 to 5499.05)	1696.18 (1358.11 to 2078.11)	2353.15 (1878.96 to 2879.17)	1066.85 (851.17 to 1305.09)	285.46 (221.45 to 370.09)
ASMR	2.77 (2.51 to 3.02)	3.14 (2.67 to 3.59)	2.56 (2.31 to 2.77)	0.30 (0.20 to 0.37)	0.38 (0.2 to 0.5)	0.25 (0.17 to 0.29)	
ASDR	67.73 (59.96 to 73.45)	69.74 (59.16 to 78.72)	67.42 (58.34 to 74.51)	11.75 (8.57 to 14.39)	14.76 (9.92 to 18.82)	9.11 (6.73 to 10.91)	50.76 (30.39 to 76.18)
*2019*^**△**^							
Incident case (× 10^5^)	4046.12 (3594.25 to 4465.48)	871.90 (780.14 to 954.2)	3174.22 (2809.71 to 3512.61)	1155.52 (930.45 to 1401.8)	761.05 (610.28 to 921.68)	394.47 (316.44 to 479.04)	112.65 (87.90 to 144.55)
Deaths (× 10^3^)	236.79 (198.43 to 259.03)	104.88 (82.41 to 117.43)	131.91 (111.61 to 145.72)	13.28 (10.62 to 16.27)	6.93 (4.5 to 9.14)	6.35 (5.17 to 8.29)	
DALYs (× 10^3^)	5201.67 (4454.04 to 5704.84)	2407.94 (1949.07 to 2706.29)	2793.74 (2402.91 to 3098.75)	604.31 (477.35 to 745.19)	362.3 (274.44 to 454.56)	242.01 (193.91 to 299.06)	1861.78 (1127.72 to 2782.26)
ASIR	5075.89 (4516.65 to 5594.10)	2211.69 (1988.65 to 2420.05)	7945.83 (7045.47 to 8792.4)	1394.03 (1126.40 to 1688.16)	1856.87 (1495.27 to 2245.34)	947.22 (761.21 to 1148.43)	280.40 (219.62 to 360.32)
ASMR	3.13 (2.61 to 3.43)	3.27 (2.54 to 3.65)	3.05 (2.58 to 3.37)	0.17 (0.14 to 0.21)	0.20 (0.13 to 0.26)	0.15 (0.12 to 0.19)	
ASDR	66.17 (56.56 to 72.5)	65.64 (52.8 to 73.33)	67.21 (57.89 to 74.67)	7.35 (5.82 to 9.04)	9.10 (6.92 to 11.34)	5.72 (4.58 to 7.07)	48.90 (29.68 to 72.63)
1990–2019*							
Incident case (%)	60.40	78.50	56.06	48.57	44.19	57.82	105.70
Deaths (%)	140.18	124.17	154.64	17.12	12.62	22.46	
DALYs (%)	68.89	63.61	73.72	16.95	17.41	16.26	110.56
ASIR (EAPC, 95% CI)	0.08 (0.04 to 0.11)	0.39 (0.37 to 0.41)	0 (− 0.04 to 0.04)	− 0.83 (− 0.92 to − 0.74)	− 1.01 (− 1.09 to − 0.92)	− 0.47 (− 0.59 to − 0.36)	− 0.03 (− 0.05 to − 0.01)
ASMR (EAPC, 95% CI)	0.55 (0.47 to 0.62)	0.28 (0.23 to 0.34)	0.70 (0.62 to 0.78)	− 2.05 (− 2.24 to − 1.86)	− 2.32 (− 2.47 to − 2.18)	− 1.88 (− 2.15 to − 1.61)	
ASDR (EAPC, 95% CI)	− 0.08 (− 0.11 to − 0.04)	− 0.17 (− 0.21 to − 0.12)	− 0.04 (− 0.09 to 0.01)	− 1.77 (− 1.91 to − 1.64)	− 1.83 (− 1.94 to − 1.72)	− 1.76 (− 1.96 to − 1.56)	− 0.06 (− 0.08 to − 0.03)

*DALYs* disability-adjusted life-years; *ASIR* age-standardized incidence rate; *ASMR* age-standardized mortality rate; *ASDR* age-standardized DALYs rate; *EAPC* estimated annual percentage change; *CI* confidence interval

^△^All data are reported as numbers or rates (per 100,000 persons with 95% uncertainty intervals)

*Percentage represents the change in cases, EAPC represents the change in rates

Compared with 1990, UTI and urolithiasis deaths increased by 140.18% and 17.12%, respectively, in 2019. The number of deaths and age-standardized mortality rate (ASMR) of UTI were 17.83 times and 18.53 times those of urolithiasis, respectively (Table 1). From 1990 to 2019, the ASMR of UTI increased from 2.77/10,000 (95% UI 2.51/100,000 to 3.02/100,000) to 3.13/100,000 (95% UI 2.61/100,000 to 3.43/100,000) with an EAPC of 0.55 (95% CI 0.47 to 0.62), while the ASMR of urolithiasis decreased from 0.30/100,000 (95% UI 0.20/100,000 to 0.37/100,000) to 0.17/100,000 (95% UI 0.14/100,000 to 0.21/100,000) with an EAPC of –2.05 (95% CI –2.24 to –1.86) (Table 1 and Fig. 1b). Overall, only urolithiasis had a significant decrease in ASMR.

Globally, UTI was attributed to the most DALYs among these three diseases, which were 8.61 times and 2.79 times that of urolithiasis and BPH, respectively. The largest increase in DALYs was found in BPH (110.56%), followed by UTI (68.89%) and urolithiasis (16.95%) during the study period. Generally, the age-standardized DALY rate (ASDR) of these three diseases showed a decreasing trend (Fig. 1c). The largest decrease was recorded in urolithiasis, with an EAPC of –1.77 (95% CI –1.91 to –1.64).

### Regional incidence, mortality, and DALYs

In 2019, more than one-fifth of UTI cases occurred in South Asia, Western Europe and Tropical Latin America. The top five regions with incident cases of urolithiasis were South Asia, East Asia, Eastern Europe, Southeast Asia, and Western Europe, which were the same as with BPH (Additional file 1: Table S1). Indeed, Eastern Europe showed the highest ASIR in urolithiasis (4433.72/100,000, 95% UI 3542.49/100,000 to 5414.66/100,000) and BPH (629.82/100,000, 95% UI 500.32/100,000 to 790.30/100,000). The highest ASIR of UTI was recorded in Andean Latin America (13,163.85/100,000, 95% UI 11,554.82/100,000 to 14,741.08/100,000) and Tropical Latin America (13,085.96/100,000, 95% UI 11,490.17/100,000 to 14,608.79/100,000). Overall, the EAPC of ASIR for these three urologic benign diseases in most regions was above zero (Table 2). Nevertheless, East Asia and High-income North America showed significant decreases in ASIRs for urolithiasis, with EAPCs of –2.68 (95% CI –2.95 to –2.42) and –2.02 (95% CI –2.33 to –1.71), respectively.

**Table 2:** EAPC of the ASIR, ASMR and ASDR of the three urologic benign diseases in global and 21 regions

Location	EAPC of ASIR (95% CI)			EAPC of ASMR (95% CI)		EAPC of ASDR (95% CI)		
	Urinary tract infections	Urolithiasis	Benign prostatic hyperplasia	Urinary tract infections	Urolithiasis	Urinary tract infections	Urolithiasis	Benign prostatic hyperplasia
Global	0.08 (0.04 to 0.11)	− 0.83 (− 0.92 to − 0.74)	− 0.03 (− 0.05 to − 0.01)	0.55 (0.47 to 0.62)	− 2.05 (− 2.24 to − 1.86)	− 0.08 (− 0.11 to − 0.04)	− 1.77 (− 1.91 to − 1.64)	− 0.06 (− 0.08 to − 0.03)
Andean Latin America	0.45 (0.40 to 0.50)	0.52 (0.44 to 0.59)	0.15 (0.03 to 0.28)	1.59 (1.10 to 2.08)	0.16 (− 0.04 to 0.37)	0.58 (0.18 to 0.98)	0.28 (0.19 to 0.36)	0.13 (0.01 to 0.26)
Australasia	0.09 (0.06 to 0.11)	− 0.35 (− 0.43 to − 0.26)	− 0.05 (− 0.08 to − 0.02)	0.86 (0.52 to 1.20)	− 2.15 (− 2.71 to − 1.59)	0.17 (− 0.06 to 0.39)	− 1.13 (− 1.41 to − 0.85)	− 0.04 (− 0.06 to − 0.02)
Caribbean	− 0.08 (− 0.08 to − 0.07)	0.66 (0.63 to 0.70)	0.06 (0 to 0.11)	2.45 (2.08 to 2.83)	1.60 (1.44 to 1.76)	1.91 (1.69 to 2.13)	1.08 (0.98 to 1.18)	0.02 (− 0.02 to 0.05)
Central Asia	0.03 (0.02 to 0.04)	0.05 (0.02 to 0.08)	0.09 (0.04 to 0.15)	2.56 (2.20 to 2.93)	1.74 (1.40 to 2.09)	1.70 (1.32 to 2.08)	0.29 (0.12 to 0.46)	0.05 (0.01 to 0.10)
Central Europe	− 0.28 (− 0.37 to − 0.20)	− 0.71 (− 0.99 to − 0.43)	− 0.17 (− 0.28 to − 0.07)	− 1.44 (− 2.14 to − 0.73)	− 6.31 (− 7.29 to − 5.32)	− 2.13 (− 2.75 to − 1.50)	− 3.18 (− 3.58 to − 2.78)	− 0.18 (− 0.27 to − 0.09)
Central Latin America	0.48 (0.30 to 0.66)	0.13 (− 0.2 to 0.47)	0.04 (0.01 to 0.06)	1.98 (1.68 to 2.29)	− 0.14 (− 0.37 to 0.08)	1.69 (1.37 to 2.01)	0.06 (− 0.19 to 0.31)	0 (− 0.03 to 0.02)
Central Sub-Saharan Africa	0.08 (0.07 to 0.09)	0.31 (0.25 to 0.37)	0.01 (0 to 0.02)	− 0.44 (− 0.51 to − 0.37)	− 0.78 (− 0.92 to − 0.64)	− 0.59 (− 0.67 to − 0.52)	− 0.54 (− 0.63 to − 0.45)	0.03 (0.02 to 0.04)
East Asia	− 0.19 (− 0.26 to − 0.13)	− 2.68 (− 2.95 to − 2.42)	− 0.09 (− 0.20 to 0.03)	− 1.15 (− 1.31 to − 0.98)	− 4.93 (− 5.21 to − 4.65)	− 2.35 (− 2.62 to − 2.07)	− 4.43 (− 4.68 to − 4.18)	− 0.16 (− 0.3 to − 0.02)
Eastern Europe	0.03 (0.02 to 0.04)	− 0.69 (− 0.84 to − 0.53)	0.04 (0 to 0.07)	− 0.43 (− 0.78 to − 0.08)	− 0.84 (− 1.28 to − 0.39)	− 0.86 (− 1.15 to − 0.58)	− 1.09 (− 1.35 to − 0.83)	0.03 (0.02 to 0.05)
Eastern Sub-Saharan Africa	0.09 (0.08 to 0.11)	0.15 (0.10 to 0.20)	0 (− 0.01 to 0.02)	− 0.51 (− 0.56 to − 0.45)	− 0.54 (− 0.58 to − 0.50)	− 0.91 (− 0.95 to − 0.87)	− 1.09 (− 1.16 to − 1.03)	0.02 (0.01 to 0.03)
High-income Asia Pacific	− 0.04 (− 0.10 to 0.03)	− 0.27 (− 0.35 to − 0.18)	− 0.09 (− 0.14 to − 0.03)	1.01 (0.70 to 1.32)	2.60 (2.33 to 2.87)	0.49 (0.29 to 0.69)	0.25 (0.16 to 0.33)	− 0.10 (− 0.14 to − 0.06)
High-income North America	− 0.30 (− 0.36 to − 0.25)	− 2.02 (− 2.33 to − 1.71)	0.12 (0.06 to 0.18)	− 0.08 (− 0.36 to 0.19)	0.80 (0.44 to 1.17)	0.02 (− 0.18 to 0.22)	− 1.01 (− 1.28 to − 0.73)	0.12 (0.07 to 0.17)
North Africa and Middle East	0.09 (0.08 to 0.10)	0.29 (0.25 to 0.33)	0.08 (0.07 to 0.10)	0.41 (0.13 to 0.69)	1.06 (0.66 to 1.47)	− 0.50 (− 0.69 to − 0.30)	0.20 (0.14 to 0.27)	0.07 (0.05 to 0.08)
Oceania	− 0.02 (− 0.04 to − 0.01)	0.14 (0.10 to 0.19)	0.19 (0.16 to 0.22)	− 0.43 (− 0.47 to − 0.39)	− 1.22 (− 1.32 to − 1.12)	− 0.30 (− 0.33 to − 0.26)	− 0.58 (− 0.63 to − 0.52)	0.22 (0.19 to 0.25)
South Asia	0.33 (0.30 to 0.35)	0.64 (0.50 to 0.78)	0.30 (0.15 to 0.44)	− 0.16 (− 0.29 to − 0.04)	− 2.65 (− 2.81 to − 2.49)	− 0.49 (− 0.56 to − 0.42)	− 1.20 (− 1.35 to − 1.05)	0.37 (0.22 to 0.52)
Southeast Asia	− 0.20 (− 0.26 to − 0.13)	− 0.82 (− 0.94 to − 0.69)	− 0.09 (− 0.19 to 0.01)	− 0.19 (− 0.29 to − 0.09)	− 0.88 (− 1.01 to − 0.75)	− 0.64 (− 0.73 to − 0.55)	− 1.15 (− 1.24 to − 1.07)	− 0.14 (− 0.26 to − 0.02)
Southern Latin America	0.12 (0.04 to 0.19)	− 0.21 (− 0.28 to − 0.14)	0.07 (0.02 to 0.11)	4.92 (4.29 to 5.56)	1.13 (0.74 to 1.52)	4.12 (3.6 to 4.64)	− 0.07 (− 0.16 to 0.02)	0.07 (0.04 to 0.09)
Southern Sub-Saharan Africa	0.01 (− 0.01 to 0.03)	0.11 (0.07 to 0.15)	0.12 (0.09 to 0.15)	− 0.08 (− 0.67 to 0.50)	− 0.17 (− 0.83 to 0.49)	− 0.39 (− 1.05 to 0.28)	− 0.22 (− 0.58 to 0.13)	0.12 (0.09 to 0.15)
Tropical Latin America	0.03 (− 0.04 to 0.11)	− 0.36 (− 0.44 to − 0.28)	− 0.03 (− 0.09 to 0.03)	3.50 (3.15 to 3.85)	4.00 (3.76 to 4.24)	2.28 (1.99 to 2.56)	2.07 (1.94 to 2.20)	− 0.05 (− 0.10 to 0.01)
Western Europe	− 0.28 (− 0.40 to − 0.15)	0.53 (0.38 to 0.68)	− 0.03 (− 0.11 to 0.04)	2.19 (1.83 to 2.54)	− 1.11 (− 1.55 to − 0.68)	1.38 (1.12 to 1.65)	− 0.03 (− 0.20 to 0.14)	− 0.08 (− 0.11 to − 0.06)
Western Sub-Saharan Africa	0.16 (0.15 to 0.17)	0.28 (0.23 to 0.33)	0.03 (0.02 to 0.04)	− 0.93 (− 1.02 to − 0.84)	− 1.29 (− 1.39 to − 1.20)	− 1.02 (− 1.11 to − 0.93)	− 0.45 (− 0.48 to − 0.42)	0.04 (0.03 to 0.05)

*EAPC* estimated annual percentage change; *ASIR* age-standardized incidence rate; *ASMR* age-standardized mortality rate; *ASDR* age-standardized DALYs rate; *CI* confidence interval

Regionally, most deaths from UTI occurred in South Asia, almost twice as many as in Western Europe. The deaths from urolithiasis were mainly distributed in East Asia, Southeast Asia, and South Asia. The highest ASMR of UTI and urolithiasis was recorded in Tropical Latin America (9.38, 95% UI 5.71 to 10.45) and Eastern Europe (0.55, 95% UI 0.45 to 0.66) (Additional file 1: Table S2). The regions with the largest increases in ASMR in UTI and urolithiasis were Southern Latin America and Tropical Latin America, with EAPCs of 4.92 (95% CI 4.29 to 5.56) and 4.00 (95% CI 3.76 to 4.24), respectively. In addition, East Asia and Central Europe showed the most significant decreasing trend of ASMR in both UTI and urolithiasis (Table 2).

Consistent with the incident cases and deaths, the region with the highest DALYs of UTI and urolithiasis was South Asia, and the region with the highest DALYs of BPH was East Asia. The highest ASDR of UTI was found in Tropical Latin America (167.29, 95% UI 114.40 to 183.79), and the highest ASDR of urolithiasis (23.61, 95% UI 18.69 to 29.23) and BPH (128.09, 95% UI 76.46 to 189.95) were found in Eastern Europe (Additional file 1: Table S3). The largest increase in the ASDR of UTI was observed in Southern Latin America, with an EAPC of 4.12 (95% CI 3.60 to 4.64) from 1990 to 2019. The EAPC of ASDR in urolithiasis and BPH in most regions was higher than the global level (Table 2). In 2019, the ASIR, ASMR, and ASDR of UTI in Tropical Latin America were higher than those in most other regions. For urolithiasis and BPH, Eastern Europe had the highest ASR in 2019.

### National incidence, mortality, and DALYs

In 2019, the three countries with the highest incidence of UTIs were India, Brazil and the USA. The most incident cases of urolithiasis and BPH were both found in India, China and the Russian Federation. The highest ASIR for UTI, urolithiasis, and BPH was found in Ecuador (15,542.88/100,000, 95% UI 13,708.11/100,000 to 17,401.43/100,000), the Russian Federation (4541.88/100,000, 95% UI 3648.94/100,000 to 5522.00/100,000), and Lithuania (661.64/100,000, 95% UI 547.15/100,000 to 790.35/100,000), respectively (Additional file 1: Table S4). From 1990 to 2019, Ecuador (EAPC = 1.09, 95% CI 0.95 to 1.24), Jordan (EAPC = 2.10, 95% CI 1.74 to 2.47), and Mauritius (EAPC = 0.42, 95% CI 0.38 to 0.46) had the largest increases in the ASIR of UTI, urolithiasis, and BPH, respectively (Additional file 1: Table S5).

India (55,558.41, 95% UI 42,788.06 to 66,161.17) had the highest deaths from UTIs, and China (2558.42, 95% UI 1733.75 to 3527.82) had the highest deaths from urolithiasis in 2019. The highest ASMRs of UTI and urolithiasis were recorded in Barbados (12.02/100,000, 95% UI 8.50/100,000 to 14.55/100,000) and Armenia (1.82/100,000, 95% UI 0.94/100,000 to 4.03/100,000), respectively (Additional file 1: Table S4). Armenia (EAPC = 8.48, 95% CI 7.28 to 9.69) and Portugal (EAPC = 8.28, 95% CI 7.36 to 9.20) had the largest increases in the ASMR of UTI. Costa Rica (EAPC = 7.18, 95% CI 6.30 to 8.06) and Georgia (EAPC = 7.12, 95% CI 5.62 to 8.64) had the largest increases in the ASMR of urolithiasis (Additional file 1: Table S6).

The highest DALYs were recorded in India for UTI and urolithiasis and in China for BPH. ASDR was highest in Tajikistan (242.35/100,000, 95% UI 172.84/100,000 to 301.91/100,000) for UTI, in Lithuania (136.44/100,000, 95% UI 85.33/100,000 to 200.10/100,000) for BPH and in Armenia (33.33/100,000, 95% UI 21.71/100,000 to 61.27/100,000) for urolithiasis (Additional file 1: Table S4). The most significant increase in ASDR was found in Portugal for UTI with an EAPC of 6.44 (95% CI 5.67 to 7.21), in Trinidad and Tobago (EAPC = 2.84, 95% CI 2.30 to 3.38) for urolithiasis and in Mauritius (EAPC = 0.46, 95% CI 0.42 to 0.49) for BPH (Additional file 1: Table S7). For these three diseases, India and China had the greatest number of incident cases, deaths, and DALYs; Armenia had the highest ASMR and ASDR of urolithiasis; and Lithuania had the highest ASIR and ASDR of BPH.

### Burden of the three urologic benign diseases by SDI

**Fig. 2:**
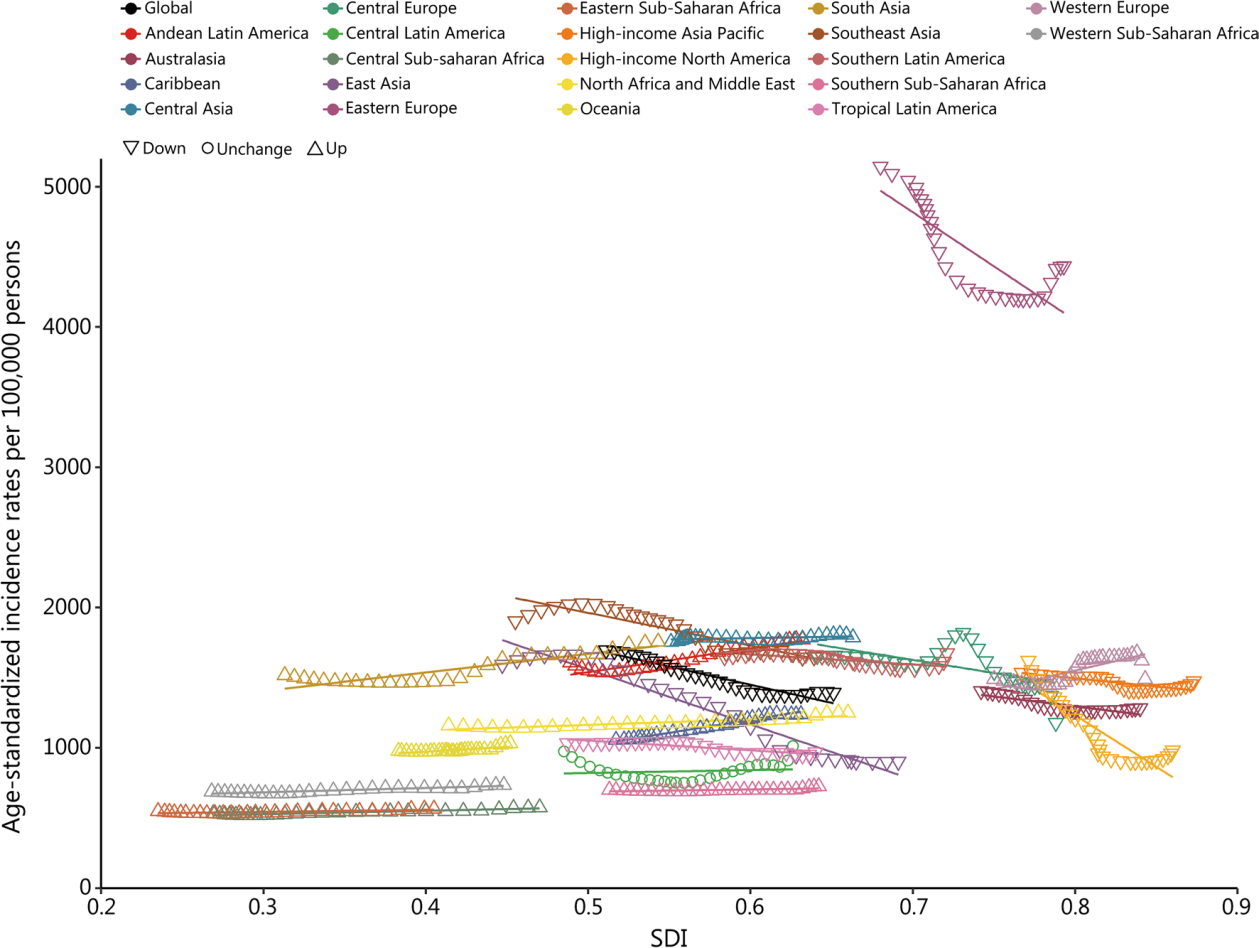
Age-standardized incidence rates for urolithiasis for 21 regions by SDI from 1990 to 2019. SDI sociodemographic index

**Fig. 3:**
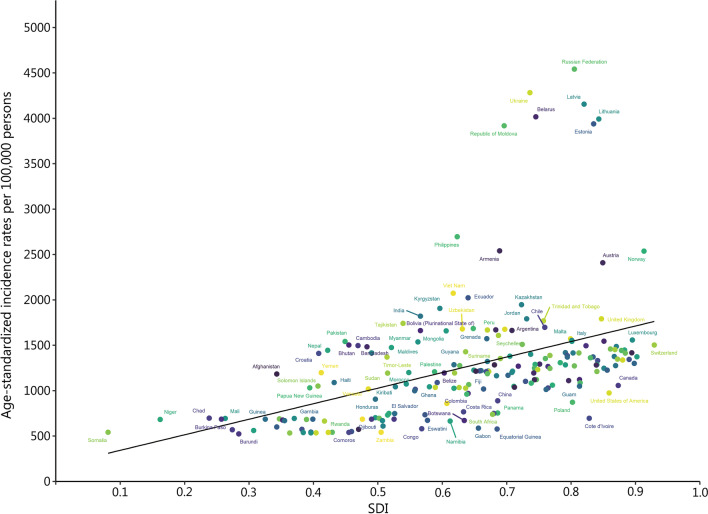
Age-standardized incidence rates for urolithiasis for 203 countries and territories by SDI in 2019. SDI sociodemographic index

The incident cases, deaths, and DALYs of UTI were mainly distributed in middle and low-middle SDI regions, while those of urolithiasis and BPH were mainly distributed in middle and high-middle SDI regions (Additional file 1: Tables S1-3). In addition, the ASIR and SDI of urolithiasis in regions with high SDI from 1990 to 2019 were negatively correlated, while the opposite trend was seen in regions with low SDI (Fig. 2). The ASDR and ASMR of urolithiasis were negatively correlated with the SDI in most GBD regions from 1990 to 2019 (Additional file 2: Figs. S1-2). Nationally, generally positive correlations between the ASIRs of these three urologic benign diseases and SDI of 203 countries and territories in 2019 were recorded (Fig. 3, Additional file 2: Figs. S3-4). The ASDR and SDI for urolithiasis and BPH in 2019 also presented a positive correlation (Additional file 2: Figs. S5-6). The ASDR and ASMR of UTI did not show obvious correlations with the SDI value at either the regional level or the national level (Additional file 2: Figs. S7-10). In addition, the ASIR and ASDR of BPH, ASIR of UTI did not show obvious correlations with SDI value at regional level, as well as ASMR of urolithiasis at the national level (Additional file 2: Figs. S11-14).

### Burden of the three urologic benign diseases by age and sex

In 2019, the incident cases of UTI were mainly concentrated in the 25 to 34 and 0 to 14-year age groups. The incident cases of urolithiasis were concentrated in the 50 to 54-year age group in both females and males. The ASIR of UTI in females was 3.59 times that of males, and the incidence was highest in the 30 to 34-year and over 80-year age groups among females and males, respectively. The ASIR of urolithiasis in males was 1.96 times higher than that of females and was highest in the 55 to 59-year age group in both sexes. For BPH, both incident cases and incidence were highest in the 65 to 69-year age group (Additional file 2: Fig. S15). The deaths and mortality rate of UTI and urolithiasis increased with age in both females and males. The ASMR was 1.36 times higher in males (0.20/100,000, 95% UI 0.13/100,000 to 0.26/100,000) with urolithiasis than in females (0.15/100,000, 95% UI 0.12/100,000 to 0.19/100,000), while males and females with UTI had roughly the same mortality across age groups (Additional file 2: Fig. S16). The DALYs of UTI mainly focus on the 0 to 14-year and over 80-year age groups, urolithiasis was mainly in the 45–54 age group, and BPH was mainly in the 65 to 69-year age group. The rate of DALYs increased with age for UTI and urolithiasis, while it was highest in the 75 to 79-year age group for BPH (Additional file 2: Fig. S17). Overall, the incidence of UTI and urolithiasis was highest in the 30 to 34-year age group among females and in the 55 to 59-year age group in both males and females, while the mortality and DALY rate increased with age in both UTI and urolithiasis. The disease burden of BPH was concentrated in the 65 to 74-year age group.

## Discussion

From 1990 to 2019, the global incident cases of these three urologic benign diseases showed a substantial increase. Meanwhile, we observed a decreasing trend of ASIR in urolithiasis and BPH during the study period, which indicates that the increase in cases may be due to population growth and the general aging process. UTI is one of the most common microbial infections in humans. Our results showed that the incidence of UTI remained much higher than those of urolithiasis and BPH, and the ASIR showed an increasing trend. The increased rates of antimicrobial resistance and changes in population structure may partially explain this phenomenon [10, 11]. UTI range in severity from mild self-limitation to severe sepsis, with a mortality rate of 20–40% [12]. In addition, for special populations of UTI such as elderly individuals, men, pregnant women, trauma patients, and patients who use urinary catheters in medical institutions, there are certain challenges in management, and the risk of death also increases [12–14]. Our results showed an increased ASMR for UTI over the past 30 years, which indicates that we need to pay more attention to those specific populations and improve treatment effects. The decrease in the ASMR of urolithiasis over the past 30 years is inseparable from the innovation of the surgical paradigm and the advancement of guidelines [7, 15]. In addition, it may be attributed to the early diagnosis and treatment of its comorbidities, such as chronic kidney disease or end-stage renal disease [16]. In these urologic benign diseases, UTI had the highest DALYs, and BPH had the most significant increase. Meanwhile, we found that the ASDR of these three urologic benign diseases decreased in recent decades. BPH often manifests as lower urinary tract symptoms (LUTS), which seriously affect quality of life. On the one hand, the aging population may explain the increased disease burden of BPH [17]. On the other hand, uneven allocation of medical resources in underdeveloped areas is still an important challenge, including the underdiagnosis, undertreatment, and disparate ratio of urologists and patients [17, 18]. In this study, we explored the global burden of these three urologic benign diseases and provided evidence for the targeted formulation of health care policies and the allocation of medical resources in the future.

At the regional and national levels, the number of incident cases, deaths and DALYs of UTI mainly distributed in South Asia and Western Europe, while the corresponding ASRs were the highest in Tropical Latin America. *Escherichia coli* is the most frequently identified uropathogen and a key organism causing dissemination of antibiotic resistance [19]. Community-acquired *Escherichia coli* resistance continued to rise in Europe in 2007–2008, with resistance to ampicillin (mean 28%) and sulfamethoxazole (mean 24%) being the most common [20]. The Global Prevalence Studies in Urology Clinics Studies 2003–2010, which included 1866 health care-associated UTI patients, showed that the global resistance rates of overall uropathogens to ciprofloxacin, trimethoprim-sulfamethoxazole, ampicillin and beta-lactamase inhibitors were all over 50% [21]. Similarly, the phenomenon was also found in American female outpatients [22]. Moreover, the resistance rate was more widespread in Asia, especially in Southeast Asia [23]. In addition, the lack of antibiotic use surveillance systems in some undeveloped areas or impropriate use of empirical antibiotics may further aggravate the antibiotic resistance problem [24, 25]. For urolithiasis and BPH, East Asia, Southeast Asia, and South Asia recorded the most incident cases, deaths and DALYs, while Eastern Europe had the highest corresponding ASR. Even so, East Asia was reported to have the largest decrease in ASMR in urolithiasis over the past three decades. In addition, we found that India had the most incident cases of these three urologic benign diseases, and China had the most deaths from urolithiasis and DALYs of BPH. China and India are the two most populous countries in the world, and the large population base and aging may be related to the most incident cases. In addition, urolithiasis has obvious geographical distribution characteristics due to factors such as mineral distribution, climate, ethnicity, and dietary habits [7]. Moreover, we found that the ASIR of these three urologic benign diseases was higher in countries with a high SDI in 2019, while the ASDR and ASMR of urolithiasis decreased with SDI in most regions from 1990 to 2019. High-SDI regions and countries may mean more patient engagements in medical care and a more complete medical and health care system. Therefore, the development of artificial intelligence-assisted medical systems and online clinic services is an effective way to help underdeveloped areas directly obtain external medical resources, which is conducive to achieving universal health coverage.

We found that 25- to 35-year-old women had the highest incidence of UTI, which is consistent with previous studies [26, 27]. Changes in behavioral factors and physiological functions may affect the incidence of UTI in women. First, the strongest risk factors for UTI in premenopausal women were sexual intercourse, use of spermicides, pregnancy, and previous UTI [28]. Second, estrogen reduction, changes in vaginal microbial flora and pH and impairment of bladder emptying function in elderly women also increase the risks. Finally, diabetes, dyslipidemia, urolithiasis, long-term indwelling catheters and neurogenic bladder are comorbid conditions that increase risk in both sexes [28, 29]. In contrast, evidence has revealed that the incidence of urolithiasis in males has been significantly higher than that in females over a century, and this sex gap is narrowing [30, 31]. Our findings showed that the ratio of males to females in ASIR has dropped from 2.21 to 1.96 in the past 30 years. This may be related to changes in diet, increases in metabolic syndrome, and innovation in surgical intervention [32, 33]. In addition, we observed that the ASMRs and ASDRs of UTI and urolithiasis dramatically increased over 70 years of age. This may be due to the poor tolerance of the elderly to severe infections and the proneness to complications, including decreased bone mineral density, cardiovascular disease, and chronic kidney disease [33]. To our knowledge, the prevalence of BPH increases with age, ranging from 50–75% over 50 years of age to 80% over 70 years of age [34]. We also found that the ASDR of BPH increased with age but decreased in individuals over 80 years old. Therefore, we inferred that most patients were treated with drug or surgical intervention in the early stage of disease, and the symptoms of LUTS/BPH were improved or controlled, so the DALYs and ASDR in the age group older than 80 years were decreased compared to those of the pre-80 age group.

Although this article comprehensively analyzed the burden of these three urologic benign diseases in different regions and countries around the world, it still had certain limitations. First, GBD data sources are limited and cannot cover all populations or regions, so the data only represent the general situation of a certain region. Second, the quality of the data is uneven, and the heterogeneity of the data can be caused by differences in the diagnostic standards, detection methods and supervision systems in regions with different levels of development. Third, due to the limitation of the definition of disease given by GBD, the burden of diseases may be underestimated.

## Conclusions

Over the past three decades, the disease burden has increased for UTIs but decreased for urolithiasis and BPH. The allocation of medical resources should be based more on the epidemiological characteristics and geographical distribution of diseases.

## Supplementary Information

**Additional file 1**: **Table S1.** Regional incident cases and ASIR of the three urologic benign diseases in 2019. **Table S2** Regional deaths and ASMR of urinary tract infections and urolithiasis in 2019. **Table S3** Regional DALYs and ASDR of the three urologic benign diseases in 2019. **Table S4** Incidence, mortality, and DALYs of the three urologic benign diseases among the top three and bottom three countries in 2019. **Table S5** EAPC of ASIR for the three urologic benign diseases in 203 countries and territories from 1990 to 2019. **Table S6** EAPC of ASMR for urinary tract infections and urolithiasis in 203 countries and territories from 1990 to 2019. **Table S7** EAPC of ASDR for the three urologic benign diseases in 203 countries and territories from 1990 to 2019.

**Additional file 2**: **Fig. S1.** Age-standardized DALYs rates for urolithiasis for 21 regions by SDI from 1990 to 2019. SDI sociodemographic index; DALYs disability-adjusted life-years. **Fig. S2** Age-standardized mortality rates for urolithiasis for 21 regions by SDI from 1990 to 2019. SDI sociodemographic index. **Fig. S3** Age-standardized incidence rates for urinary tract infections for 203 countries and territories by SDI in 2019. SDI sociodemographic index. **Fig. S4** Age-standardized incidence rates for benign prostatic hyperplasia for 203 countries and territories by SDI in 2019.SDI sociodemographic index. **Fig. S5** Age-standardized DALYs rates for urolithiasis for 203 countries and territories by SDI in 2019. SDI sociodemographic index; DALYs disability-adjusted life-years. **Fig. S6** Age-standardized DALYs rates for benign prostatic hyperplasia for 203 countries and territories by SDI in 2019. SDI sociodemographic index; DALYs disability-adjusted life-years. **Fig. S7** Age-standardized DALYs rates for urinary tract infections for 203 countries and territories by SDI in 2019. SDI sociodemographic index; DALYs disability-adjusted life-years. **Fig. S8** Age-standardized mortality rates for urinary tract infections for 203 countries and territories by SDI in 2019. SDI sociodemographic index. **Fig. S9** Age-standardized DALYs rates for urinary tract infections for 21 regions by SDI from 1990 to 2019. SDI sociodemographic index; DALYs disability-adjusted life-years. **Fig. S10** Age-standardized mortality rates for urinary tract infections for 21 regions by SDI from 1990 to 2019. SDI sociodemographic index. **Fig. S11** Age-standardized mortality rates for urolithiasis for 203 countries and territories by SDI in 2019. SDI sociodemographic index. **Fig. S12** Age-standardized incidence rates for urinary tract infections for 21 regions by SDI from 1990 to 2019. SDI sociodemographic index. **Fig. S13** Age-standardized incidence rates for benign prostatic hyperplasia for 21 regions by SDI from 1990 to 2019. SDI sociodemographic index. **Fig. S14** Age-standardized DALYs rates for benign prostatic hyperplasia for 21 regions by SDI from 1990 to 2019. SDI sociodemographic index; DALYs disability-adjusted life-years. **Fig. S15** Global incidence of the three urologic benign diseases by age and sex in 2019. **a** Urinary tract infections. **b** Urolithiasis. **c** Benign prostatic hyperplasia. **Fig. S16** Global mortality of urinary tract infections and urolithiasis by age and sex in 2019. **a** Urinary tract infections. **b** Urolithiasis. **Fig. S17** Global DALYs of the three urologic benign diseases by age and sex in 2019. **a** Urinary tract infections. **b** Urolithiasis. **c** Benign prostatic hyperplasia.

## Data Availability

The datasets generated during the current study are available in the Global Health Data Exchange query tool (http://ghdx.healthdata.org/gbd-results-tool).

## Abbreviations

ASR: Age-standardized rate
ASDR: Age-standardized DALYs rate
ASMR: Age-standardized mortality rate
ASIR: Age-standardized incidence rate
BPH: Benign prostatic hyperplasia
CI: Confidence interval
DALYs: Disability-adjusted life-years
DW: Disability weight
EAPC: Estimated annual percentage changes
GBD 2019: Global Burden of Disease Study 2019
ICD: International Classification of Diseases
SDI: Sociodemographic index
UI: Uncertainty interval
UTI: Urinary tract infections
YLD: Years lived with disability
YLL: Years of life lost
