# Supplementary material for: Epidemiological trends of urinary tract infections, urolithiasis and benign prostatic hyperplasia in 203 countries and territories from 1990 to 2019
Source: Mil Med Res. 2021 Dec 9;8:64. doi: 10.1186/s40779-021-00359-8 (PMC8656041; doi:10.1186/s40779-021-00359-8)
Supplement: Supplementary file 1 — Additional file 1: Table S1. Regional incident cases and ASIR of the three urologic benign diseases in 2019. Table S2 Regional deaths and ASMR of urinary tract infections and urolithiasis in 2019. Table S3 Regional DALYs and ASDR of the three urologic benign diseases in 2019. Table S4 Incidence, mortality, and DALYs of the three urologic benign diseases among the top three and bottom three countries in 2019. Table S5 EAPC of ASIR for the three urologic benign diseases in 203 countries and territories from 1990 to 2019. Table S6 EAPC of ASMR for urinary tract infections and urolithiasis in 203 countries and territories from 1990 to 2019. Table S7 EAPC of ASDR for the three urologic benign diseases in 203 countries and territories from 1990 to 2019. [file 40779_2021_359_MOESM1_ESM.pdf]

Protocol of the systematic literature review of urolithiasis.

For GBD 2010, a systematic review of the prevalence of acute urolithiasis throughout the world was conducted. This search was updated for GBD 2013 and again for GBD 2016. In the 2019 GBD, these data were also used to estimate the prevalence and incidence of urolithiasis, as well as hospital discharge and claim data.

The search terms used in PubMed as follow: (Urolithiasis[Title/Abstract] OR Kidney Stones[Title/Abstract]) AND (Prevalence[Title/Abstract] OR Incidence[Title/Abstract]) AND ("2013/01/01"[PDAT] : "3000"[PDAT]) AND "humans"[MeSH Terms].

The exclusion criteria:

1. Studies clearly not representative of the national population
2. Studies that did not provide primary data on epidemiological parameters, eg, a commentary piece
3. Studies of a specific type of urolithiasis

Table S1 Regional incident cases and ASIR of the three urologic benign diseases in 2019

	Urinary tract infections		Urolithiasis		Benign prostatic hyperplasia	
	**Incident cases No.×10^5 (95% UI)**	**ASIR per 100,000 population (95% UI)**	**Incident cases No.×10^5 (95% UI)**	**ASIR per 100,000 population (95% UI)**	**Incident cases No.×10^5 (95% UI)**	**ASIR per 100,000 population (95% UI)**
Global	4046.12 (3594.25 to 4465.48)	5075.89 (4516.65 to 5594.1)	1155.52 (930.45 to 1401.8)	1394.03 (1126.4 to 1688.16)	112.65 (87.9 to 144.55)	280.40 (219.62 to 360.32)
Low SDI	440.61 (381.98 to 495.31)	4678.93 (4086.05 to 5197.27)	78.71 (61.91 to 97.3)	981.88 (771.35 to 1212.27)	5.17 (3.92 to 6.76)	200.15 (151.88 to 264.06)
Low-middle SDI	1027.72 (900.26 to 1144.27)	5923.11 (5221.31 to 6569.32)	242.92 (193.66 to 298.7)	1460.63 (1159.28 to 1788.45)	20.76 (15.82 to 27.03)	307.22 (235.58 to 400.8)
Middle SDI	1106.45 (973.66 to 1223.9)	4391.91 (3898.24 to 4855.12)	335.94 (269.48 to 410.44)	1242.73 (1000.94 to 1510.56)	34.16 (26.64 to 44.1)	272.30 (212.87 to 351.87)
High-middle SDI	715.26 (634.4 to 788.18)	4628.14 (4134.87 to 5088.58)	294.33 (233.29 to 359.43)	1576.44 (1268.89 to 1918.41)	30.37 (23.73 to 38.89)	311.27 (243.32 to 398.47)
High SDI	729.52 (658.87 to 797.01)	6424.36 (5789.95 to 7027.05)	175.25 (143.15 to 211.86)	1288.65 (1053.86 to 1544.09)	17.3 (13.89 to 21.53)	207.64 (167.64 to 257.88)
Andean Latin America	84.05 (73.61 to 94.56)	13,163.85 (11,554.82 to 14,741.08)	11.07 (9.17 to 13.24)	1772.43 (1472.6 to 2110.69)	1.07 (0.81 to 1.38)	394.79 (298.63 to 508.45)
Australasia	29.61 (25.82 to 33.1)	9663.39 (8364.96 to 10868.94)	4.77 (3.74 to 5.9)	1283.37 (1004.68 to 1573.74)	0.55 (0.41 to 0.72)	237.28 (179.69 to 313.44)
Caribbean	40.73 (35.86 to 45.49)	8417.05 (7407.77 to 9375.77)	6.32 (4.96 to 7.88)	1239.73 (979.28 to 1540.36)	0.78 (0.59 to 1.03)	314.62 (237.33 to 417.48)
Central Asia	56.00 (49.79 to 62)	5970.63 (5349.58 to 6589.72)	16.55 (13.15 to 20.32)	1787.98 (1435.54 to 2174.91)	1.00 (0.77 to 1.3)	289.42 (224.58 to 376.01)
Central Europe	57.15 (51.75 to 62.98)	4448.93 (4062.8 to 4850.1)	17.74 (14.61 to 21.44)	1178.91 (977.08 to 1400.97)	3.26 (2.6 to 4.07)	341.13 (276.85 to 420.8)
Central Latin America	195.05 (175.59 to 215.25)	7877.88 (7121.34 to 8695.4)	25.84 (20.62 to 31.27)	1012.43 (810.44 to 1222.61)	4.86 (3.89 to 6.05)	436.91 (349.5 to 544.21)
Central Sub-Saharan Africa	38.94 (33.53 to 44.25)	3430.24 (2964.2 to 3886.98)	5.32 (4.13 to 6.57)	575.37 (446.63 to 711.22)	0.32 (0.23 to 0.43)	127.43 (94.12 to 171.33)
East Asia	231.94 (201.86 to 262.76)	1231.34 (1073.38 to 1381.03)	185.31 (147.86 to 226.69)	901.81 (727.27 to 1088.77)	29.65 (22.76 to 39.16)	280.96 (216.89 to 368.91)
Eastern Europe	188.36 (166.62 to 209.71)	8593.74 (7657.11 to 9508.02)	127.34 (102.01 to 156.01)	4433.72 (3542.49 to 5414.66)	9.04 (7.05 to 11.46)	629.82 (500.32 to 790.30)

	Urinary tract infections		Urolithiasis		Benign prostatic hyperplasia	
	Incident cases No.×10^5 (95% UI)	ASIR per 100,000 population (95% UI)	Incident cases No.×10^5 (95% UI)	ASIR per 100,000 population (95% UI)	Incident cases No.×10^5 (95% UI)	ASIR per 100,000 population (95% UI)
Eastern Sub-Saharan Africa	118.43 (102.3 to 133.93)	3521.32 (3067.27 to 3920.14)	15.43 (12.13 to 19.04)	565.68 (444.29 to 692.31)	1.05 (0.77 to 1.4)	132.58 (97.64 to 177.49)
High-income Asia Pacific	119.70 (109.16 to 130.58)	6342.29 (5751.94 to 6915.04)	39.47 (31.61 to 48.36)	1475.15 (1172.93 to 1795.88)	2.48 (1.86 to 3.25)	137.75 (104.23 to 181.27)
High-income North America	301.19 (277.6 to 327.39)	6768.74 (6234.02 to 7297.77)	47.4 (40.69 to 505.62)	982.95 (843.8 to 1137.38)	5.54 (4.64 to 6.67)	195.81 (165.9 to 233.77)
North Africa and Middle East	274.77 (238.94 to 307.31)	4386.18 (3855.51 to 4886.58)	74.48 (58.35 to 93.54)	1250.71 (985.87 to 1553.28)	2.53 (1.87 to 3.38)	110.72 (81.62 to 147.69)
Oceania	2.11 (1.81 to 2.41)	1828.56 (1570.93 to 2080.35)	1.10 (0.85 to 1.39)	1033.1 (799.54 to 1296.18)	0.13 (0.1 to 0.18)	374.69 (282.76 to 494.07)
South Asia	1266.51 (1105.11 to 1414.37)	7054.27 (6196.43 to 7835.85)	307.3 (241.42 to 382.42)	1757.72 (1382.75 to 2184.63)	25.18 (19.04 to 32.84)	348.05 (264.35 to 455.89)
Southeast Asia	168.08 (145.63 to 189.02)	2363.15 (2055.34 to 2643.39)	118.03 (95.43 to 142.76)	1652.63 (1348.2 to 1979.06)	11.68 (9.01 to 15.04)	390.85 (305.31 to 505.6)
Southern Latin America	52.48 (45.71 to 58.48)	7407.28 (6415.3 to 8249.83)	12.39 (9.58 to 15.6)	1674.47 (1295.15 to 2119.25)	0.54 (0.4 to 0.72)	143.46 (105.41 to 192.59)
Southern Sub-Saharan Africa	40.36 (34.76 to 45.50)	5084.92 (4407.11 to 5699.31)	5.46 (4.3 to 6.79)	725.49 (574.19 to 893.26)	0.48 (0.36 to 0.64)	193.24 (143.27 to 257.61)
Tropical Latin America	309.75 (271.35 to 346.13)	13,085.96 (11,490.17 to 14,608.79)	24.37 (19.77 to 29.26)	969.93 (789.43 to 1165.79)	2.29 (1.84 to 2.83)	201.11 (161.3 to 249.09)
Western Europe	318.26 (276.89 to 356.06)	7195.94 (6192.47 to 8136.97)	88.06 (70.37 to 108.74)	1490.21 (1181.37 to 1829.24)	9.08 (7.19 to 11.41)	243.82 (194.49 to 304.81)
Western Sub-Saharan Africa	152.64 (132.15 to 171.74)	3991.92 (3499.37 to 4451.57)	21.77 (17.28 to 26.67)	735.82 (579.31 to 902.54)	1.16 (0.86 to 1.55)	127.28 (94.42 to 170.92)

ASIR age-standardized incidence rate; *UI* uncertainty interval; *SDI* sociodemographic index

Table S2 Regional deaths and ASMR of urinary tract infections and urolithiasis in 2019

	Urinary tract infections		Urolithiasis	
	**Deaths No.×10^3 (95% UI)**	**ASMR per 100,000 population (95% UI)**	**Deaths No. (95% UI)**	**ASMR per 100,000 population (95% UI)**
Global	236.79 (198.43 to 259.03)	3.13 (2.61 to 3.43)	13278.95 (10615.96 to 16267.44)	0.17 (0.14 to 0.21)
Low SDI	18.04 (15.06 to 21.59)	3.72 (3.06 to 4.47)	656.81 (416 to 961.33)	0.15 (0.1 to 0.23)
Low-middle SDI	56.55 (43.24 to 65.6)	4.63 (3.58 to 5.36)	2754.55 (1396.33 to 3661.86)	0.22 (0.11 to 0.29)
Middle SDI	48.41 (42.11 to 54.11)	2.33 (2 to 2.64)	4114.06 (2820.73 to 5270.27)	0.18 (0.13 to 0.24)
High-middle SDI	49.93 (39.6 to 54.48)	2.62 (2.07 to 2.86)	3505.03 (3023.55 to 4339.08)	0.18 (0.15 to 0.22)
High SDI	63.74 (51.45 to 69.97)	2.77 (2.28 to 3.02)	2241.02 (1766.68 to 3027.61)	0.10 (0.08 to 0.14)
Andean Latin America	2.13 (1.44 to 2.73)	4.01 (2.7 to 5.13)	35.73 (17.07 to 49.87)	0.06 (0.03 to 0.09)
Australasia	1.33 (1.06 to 1.51)	2.26 (1.83 to 2.56)	54.84 (44.27 to 74.11)	0.10 (0.08 to 0.14)
Caribbean	1.54 (1.14 to 1.83)	2.99 (2.21 to 3.55)	91.54 (67.69 to 122.61)	0.18 (0.13 to 0.24)
Central Asia	3.35 (2.79 to 3.78)	4.55 (3.71 to 5.13)	243.38 (186.59 to 356.33)	0.44 (0.32 to 0.69)
Central Europe	3.86 (3.32 to 4.6)	1.75 (1.5 to 2.09)	143.69 (114.27 to 185.12)	0.07 (0.05 to 0.08)
Central Latin America	9.79 (7.89 to 11.31)	4.34 (3.49 to 5.01)	491.07 (393.81 to 688.17)	0.21 (0.17 to 0.29)
Central Sub-Saharan Africa	1.21 (0.69 to 2)	2.59 (1.45 to 4.31)	56.44 (28.68 to 95.4)	0.11 (0.05 to 0.19)
East Asia	10.61 (8.96 to 13.13)	0.66 (0.56 to 0.8)	2696.26 (1832.17 to 3657.5)	0.15 (0.10 to 0.2)
Eastern Europe	10.64 (9.12 to 12.08)	3.17 (2.72 to 3.61)	1892.87 (1549.38 to 2284.08)	0.55 (0.45 to 0.66)

	Urinary tract infections		Urolithiasis	
	**Deaths No.×10^3 (95% UI)**	**ASMR per 100,000 population (95% UI)**	**Deaths No. (95% UI)**	**ASMR per 100,000 population (95% UI)**
Eastern Sub-Saharan Africa	4.08 (2.3 to 6.54)	2.69 (1.47 to 4.38)	230.13 (131.56 to 411.51)	0.20 (0.12 to 0.34)
High-income Asia Pacific	9.97 (7.27 to 11.47)	1.56 (1.18 to 1.78)	704.88 (503.71 to 963.51)	0.11 (0.09 to 0.15)
High-income North America	24.47 (20.94 to 27.46)	3.44 (2.96 to 3.85)	623.69 (512.28 to 848.59)	0.09 (0.08 to 0.13)
North Africa and Middle East	3.75 (3.23 to 4.79)	1.13 (0.96 to 1.46)	116.07 (70 to 150.67)	0.03 (0.02 to 0.04)
Oceania	0.17 (0.13 to 0.21)	2.84 (2.28 to 3.66)	9.08 (3.49 to 14.8)	0.12 (0.05 to 0.2)
South Asia	66.77 (51.04 to 78.78)	5.36 (4.12 to 6.28)	2082.26 (1223.76 to 3081.7)	0.16 (0.1 to 0.25)
Southeast Asia	16.87 (12.46 to 21.21)	3.17 (2.4 to 4.11)	2184.99 (695.03 to 2947.24)	0.40 (0.13 to 0.55)
Southern Latin America	6.66 (4.1 to 7.5)	7.71 (4.76 to 8.68)	33.65 (25.58 to 44.22)	0.04 (0.03 to 0.05)
Southern Sub-Saharan Africa	0.64 (0.52 to 0.8)	1.26 (1.04 to 1.59)	28.72 (20.02 to 38.84)	0.05 (0.04 to 0.07)
Tropical Latin America	20.99 (12.87 to 23.37)	9.38 (5.71 to 10.45)	551.60 (433.31 to 931.34)	0.23 (0.18 to 0.39)
Western Europe	33.75 (24.15 to 37.55)	2.85 (2.07 to 3.16)	883.59 (728.78 to 1236.95)	0.08 (0.07 to 0.12)
Western Sub-Saharan Africa	4.19 (3.19 to 6.09)	2.17 (1.67 to 3.19)	124.46 (52.96 to 175.06)	0.08 (0.03 to 0.11)

ASMR age-standardized mortality rate; *UI* uncertainty interval; *SDI* socio-demographic index

Table S3 Regional DALYs and ASDR of the three urologic benign diseases in 2019

	Urinary tract infections		Urolithiasis		BPH	
	DALYs No.×10³ (95% UI)	ASDR per 100,000 population (95% UI)	DALYs No.×10³ (95% UI)	ASDR per 100,000 population (95% UI)	DALYs No.×10³ (95% UI)	ASDR per 100,000 population (95% UI)
Global	5201.67 (4454.04 to 5704.84)	66.17 (56.56 to 72.5)	604.31 (477.35 to 745.19)	7.35 (5.82 to 9.04)	1861.78 (1127.72 to 2782.26)	48.90 (29.68 to 72.63)
Low SDI	634.39 (532.89 to 759.54)	86.86 (72.95 to 103.86)	40.90 (29.58 to 54.79)	5.88 (4.33 to 7.87)	89.79 (53.32 to 137.7)	39.24 (23.19 to 59.44)
Low-middle SDI	1652.13 (1275.24 to 1897.46)	110.74 (85.51 to 127.52)	135.96 (97.54 to 173.9)	8.85 (6.24 to 11.25)	341.66 (202.4 to 518.23)	55.28 (33.08 to 83.44)
Middle SDI	1209.96 (1060.41 to 1318.77)	50.88 (44.77 to 55.65)	194.31 (150.5 to 240.02)	7.49 (5.81 to 9.17)	598.43 (357.82 to 897.79)	52.61 (31.72 to 78.37)
High-middle SDI	895.01 (781.34 to 969.55)	48.05 (42.17 to 52.04)	152.52 (121.89 to 187.14)	7.94 (6.33 to 9.79)	509.49 (308.29 to 760.32)	55.03 (33.36 to 81.73)
High SDI	807.62 (687.33 to 874.64)	42.21 (36.29 to 45.8)	80.26 (62.62 to 100.6)	5.30 (3.99 to 6.75)	321.40 (198.49 to 468.42)	37.08 (22.77 to 54.05)
Andean Latin America	43.38 (31.51 to 54.26)	75.84 (54.77 to 95)	3.93 (2.83 to 5.24)	6.40 (4.62 to 8.44)	18.84 (11.26 to 28.59)	71.93 (43.12 to 108.59)
Australasia	16.22 (13.71 to 18.18)	33.28 (28.23 to 37.79)	2.18 (1.67 to 2.8)	5.37 (4.04 to 6.95)	9.36 (5.48 to 14.38)	39.09 (22.85 to 60.23)
Caribbean	33.49 (25.59 to 39.8)	67.55 (51.67 to 80.22)	4.16 (3.21 to 5.32)	8.14 (6.26 to 10.45)	13.32 (7.8 to 20.6)	55.16 (32.24 to 85.06)
Central Asia	119.59 (100.85 to 135.52)	137.71 (115.62 to 155.19)	9.6 (7.57 to 12)	12.42 (9.88 to 15.89)	15.19 (8.92 to 22.99)	51.71 (30.52 to 77.53)
Central Europe	66.10 (57.05 to 81.68)	33.59 (29.04 to 41.66)	7.43 (5.68 to 9.61)	4.52 (3.4 to 5.95)	58.44 (36.42 to 85.66)	61.83 (38.57 to 90.21)
Central Latin America	213.67 (174.13 to 246.05)	90.34 (73.45 to 104.18)	20.59 (16.74 to 26.31)	8.24 (6.7 to 10.57)	86.21 (52.66 to 127.62)	81.77 (50.21 to 120.62)
Central Sub-Saharan Africa	42.14 (25.94 to 68.61)	58.18 (34.46 to 94.69)	3.29 (2.19 to 4.73)	4.33 (2.77 to 6.34)	4.52 (2.57 to 7.24)	21.78 (12.59 to 34.1)
East Asia	223.74 (191.76 to 277.02)	12.33 (10.64 to 15.16)	107.56 (84.19 to 134.39)	5.35 (4.17 to 6.69)	427.49 (253.82 to 656.07)	45.04 (26.69 to 68.95)
Eastern Europe	246.28 (216.01 to 283.56)	80.85 (71.16 to 93.34)	72.97 (58.62 to 89.34)	23.61 (18.69 to 29.23)	167.50 (99.12 to 253.41)	128.09 (76.46 to 189.95)

	Urinary tract infections		Urolithiasis		BPH	
	**DALYs No.×10³ (95% UI)**	**ASDR per 100,000 population (95% UI)**	**DALYs No.×10³ (95% UI)**	**ASDR per 100,000 population (95% UI)**	**DALYs No.×10³ (95% UI)**	**ASDR per 100,000 population (95% UI)**
Eastern Sub-Saharan Africa	142.05 (87.32 to 222.41)	60.91 (35.39 to 96.12)	10.68 (6.94 to 16.21)	4.83 (3.18 to 7.5)	15.94 (9.17 to 24.82)	22.97 (13.18 to 35.54)
High-income Asia Pacific	111.49 (88.19 to 125.49)	24.09 (19.67 to 27.37)	18.89 (14.52 to 23.73)	5.70 (4.18 to 7.45)	47.67 (28 to 73.05)	23.84 (14.03 to 36.17)
High-income North America	338.18 (303.64 to 368.11)	54.87 (49.37 to 59.69)	23.41 (18.77 to 29.01)	4.52 (3.59 to 5.63)	100.74 (64.28 to 147.67)	34.93 (22.38 to 51.39)
North Africa and Middle East	95.49 (82.69 to 112.48)	21.30 (18.53 to 25.89)	23.56 (15.9 to 32.82)	4.10 (2.82 to 5.65)	40.59 (23.34 to 63.44)	19.59 (11.4 to 30.3)
Oceania	6.07 (4.65 to 7.71)	64.34 (51.17 to 80.51)	0.62 (0.38 to 0.87)	6.25 (3.86 to 8.8)	1.92 (1.12 to 2.94)	64.36 (38 to 97.96)
South Asia	1997.24 (1528.22 to 2312.24)	130.35 (99.88 to 150.84)	135.5 (97.92 to 180.67)	8.33 (6 to 11.1)	417.82 (244.56 to 640.9)	63.57 (37.47 to 96.51)
Southeast Asia	444.66 (324.09 to 525.14)	71.52 (52.77 to 86.15)	85.75 (46.07 to 109.02)	13.06 (6.92 to 16.54)	184.29 (108.34 to 277.06)	69.74 (41.8 to 104.04)
Southern Latin America	95.77 (61.24 to 106.19)	115.15 (74.47 to 127.34)	4.15 (2.84 to 5.76)	5.54 (3.78 to 7.74)	9.21 (5.31 to 14.39)	24.82 (14.39 to 38.57)
Southern Sub-Saharan Africa	20.91 (16.5 to 25.65)	31.57 (25.23 to 38.66)	2.50 (1.87 to 3.26)	3.46 (2.63 to 4.44)	7.64 (4.5 to 12.04)	34.61 (20.18 to 53.54)
Tropical Latin America	383.07 (261.59 to 420.12)	167.29 (114.40 to 183.79)	21.19 (16.92 to 29.89)	8.55 (6.84 to 12.13)	36.95 (22.67 to 54.74)	34.28 (20.95 to 50.33)
Western Europe	384.48 (281.53 to 422.21)	39.75 (30.31 to 43.98)	36.84 (27.51 to 47.44)	5.55 (3.99 to 7.28)	180.11 (110.46 to 263.37)	44.90 (27.41 to 65.65)
Western Sub-Saharan Africa	177.66 (137.31 to 248.13)	53.90 (41.58 to 76.62)	9.52 (6.55 to 12.65)	3.61 (2.42 to 4.79)	18.03 (10.42 to 28.17)	22.18 (12.73 to 34.32)

DALYs disability-adjusted life-years; *ASDR* age-standardized DALYs rate; *UI* uncertainty interval; *SDI* sociodemographic index

Table S4 Incidence, mortality, and DALYs of the three urologic benign diseases among the top three and bottom three countries in 2019

Measure	Type	Sex	Top three countries			Bottom three countries		
Incident cases	UTI	Both	India (100,778,307.20)	Brazil (30,026,723.04)	USA (26,945,246.94)	Tokelau (26.23)	Niue (36.78)	Nauru (171.10)
		Male	India (27,832,258.69)	Brazil (5,170,471.90)	USA (4,431,363.38)	Tokelau (5.37)	Niue (7.09)	Nauru (28.69)
		Female	India (72,946,048.50)	Brazil (24,856,251.14)	USA (22,513,883.56)	Tokelau (20.86)	Niue (29.69)	Nauru (142.40)
	Urolithiasis	Both	India (25,291,358.92)	China (17,684,919.02)	Russian Federation (9,060,658.47)	Tokelau (13.91)	Niue (20.57)	Nauru (81.88)
		Male	India (16,179,034.50)	China (12,200,202.89)	Russian Federation (5,805,667.04)	Tokelau (9.47)	Niue (13.91)	Nauru (54.36)
		Female	India (9,112,324.42)	China (5,484,716.14)	Russian Federation (3,254,991.43)	Tokelau (4.44)	Niue (6.66)	Nauru (27.52)
	BPH	male	China (2,836,451.39)	India (2,144,039.59)	Russian Federation (627,099.31)	Tokelau (2.67)	Niue (3.91)	Nauru (6.21)
Deaths	UTI	Both	India (55,558.41)	USA (22,336.19)	Brazil (20,747.07)	Tokelau (0.03)	Niue (0.06)	Cook Islands (0.06)
		Male	India (27,057.73)	Brazil (8178.42)	USA (7780.56)	Tokelau (0.01)	Cook Islands (0.02)	Niue (0.02)
		Female	India (28,500.68)	USA (14,555.63)	Brazil (12,568.65)	Tokelau (0.02)	Niue (0.03)	Cook Islands (0.04)
	Urolithiasis	Both	China (2558.42)	India (1749.82)	Russian Federation (1439.81)	Tokelau (0)	Niue (0)	Nauru (0.01)
		Male	China (1723.67)	India (991.79)	Indonesia (716.68)	Tokelau (0)	Niue (0)	Cook Islands (0)
		Female	Russian Federation (902.91)	China (834.75)	India (758.03)	Tokelau (0)	Niue (0)	Greenland (0)
DALYs	UTI	Both	India (1,587,222.29)	Brazil (378,011.92)	USA (312,209.62)	Tokelau (0.79)	Niue (1.18)	Cook Islands (1.94)
		Male	India (757,889.30)	Brazil (158,957.10)	Pakistan (133,789.02)	Tokelau (0.26)	Niue (0.52)	Cook Islands (0.84)
		Female	India (829,332.99)	Brazil (219,054.82)	USA (201,529.82)	Tokelau (0.53)	Niue (0.66)	Cook Islands (1.10)

Measure	Type	Sex	Top three countries			Bottom three countries		
	Urolithiasis	Both	India (111,246.25)	China (102,209.20)	Russian Federation (52,587.52)	Tokelau (0.08)	Niue (0.10)	Nauru (0.53)
		Male	China (69,524.91)	India (66,658.19)	Russian Federation (26,983.05)	Tokelau (0.03)	Niue (0.05)	Nauru (0.25)
		Female	India (44,588.06)	China (32,684.29)	Russian Federation (25,604.47)	Tokelau (0.04)	Niue (0.05)	Nauru (0.28)
	BPH	male	China (406,212.26)	India (355,539.28)	Russian Federation (114,499.72)	Tokelau (0.43)	Niue (0.63)	Nauru (0.80)
ASIR ($1/10^5$)	UTI	Both	Ecuador (15,542.88)	Paraguay (13,559.18)	Brazil (13,075.47)	Democratic People's Republic of Korea (1210.52)	China (1228.31)	Papua New Guinea (1793.15)
		Male	Paraguay (6209.57)	Bhutan (5186.48)	Bangladesh (5133.83)	China (305.03)	Democratic People's Republic of Korea (360.77)	Solomon Islands (671.61)
		Female	Ecuador (26,717.29)	Brazil (21,199.59)	Paraguay (21,137.72)	Democratic People's Republic of Korea (2074.56)	China (2173.89)	Papua New Guinea (2962.87)
	Urolithiasis	Both	Russian Federation (4541.88)	Ukraine (4282.60)	Latvia (4156.67)	Burundi (525.01)	South Sudan (533.43)	Madagascar (535.88)
		Male	Russian Federation (6529.33)	Latvia (6222.38)	Ukraine (6037.09)	Eritrea (417.29)	South Sudan (417.49)	Somalia (417.69)
		Female	Russian Federation (2963.78)	Ukraine (2836.23)	Lithuania (2789.94)	Lesotho (454.42)	Zimbabwe (455.50)	Eswatini (457.18)
	BPH	male	Lithuania (661.64)	Russian Federation (631.78)	Ukraine (626.98)	Turkey (107.30)	Syrian Arab Republic (107.37)	Yemen (107.46)
ASMR ($1/10^5$)	UTI	Both	Barbados (12.02)	Seychelles (11.03)	Brunei Darussalam (10.62)	North Macedonia (0.19)	Montenegro (0.25)	Cook Islands (0.30)
		Male	Barbados (16.16)	Saint Kitts and Nevis (14.76)	Seychelles (13.17)	North Macedonia (0.24)	Cook Islands (0.25)	Egypt (0.30)
		Female	Brunei Darussalam (10.88)	Brazil (9.77)	Seychelles (9.26)	North Macedonia (0.15)	Montenegro (0.20)	Albania (0.24)
	Urolithiasis	Both	Armenia (1.82)	Kazakhstan (0.88)	Philippines (0.65)	North Macedonia (0)	Montenegro (0)	Romania (0.01)
		Male	Armenia (1.56)	Kazakhstan (1.09)	Philippines (0.96)	North Macedonia (0.01)	Montenegro (0.01)	United Republic of Tanzania (0.01)
		Female	Armenia (1.96)	Kazakhstan (0.77)	Russian Federation (0.59)	North Macedonia (0)	Montenegro (0)	Albania (0.01)

Measure	Type	Sex	Top three countries			Bottom three countries		
ASDR (1/10^5)	UTI	Both	Tajikistan (242.35)	Barbados (215.91)	Turkmenistan (205.59)	North Macedonia (6.82)	Montenegro (8.00)	Egypt (9.21)
		Male	Barbados (268.23)	Tajikistan (259.51)	Armenia (256.11)	North Macedonia (5.70)	Montenegro (7.06)	Egypt (7.90)
		Female	Tajikistan (228.61)	Turkmenistan (198.96)	Pakistan (190.27)	North Macedonia (8.14)	China (8.78)	Montenegro (9.26)
	Urolithiasis	Both	Armenia (33.33)	Russian Federation (24.65)	Philippines (22.66)	Cabo Verde (2.28)	Panama (2.49)	El Salvador (2.57)
		Male	Armenia (34.62)	Philippines (33.81)	Russian Federation (31.01)	United Republic of Tanzania (1.45)	Madagascar (1.59)	Comoros (1.62)
		Female	Armenia (31.68)	Russian Federation (19.94)	Kazakhstan (18.30)	Zimbabwe (1.68)	Cabo Verde (1.82)	Maldives (1.99)
	BPH	male	Lithuania (136.44)	Russian Federation (128.45)	Ukraine (127.62)	Syrian Arab Republic (18.81)	Yemen (18.86)	Lebanon (18.88)

DALYs disability-adjusted life-years; *ASIR* age-standardized incidence rate; *ASMR* age-standardized mortality rate; *ASDR* age-standardized DALYs rate

Table S5 EAPC of ASIR for the three urologic benign diseases in 203 countries and territories from 1990 to 2019

Location	EAPC (95% CI)		
	**Urinary tract infections**	**Urolithiasis**	**Benign prostatic hyperplasia**
**Afghanistan**	0 (–0.03 to 0.03)	0.28 (0.23 to 0.34)	0.11 (0.09 to 0.12)
**Albania**	0.08 (0.07 to 0.09)	0.02 (–0.01 to 0.06)	0.08 (0 to 0.16)
**Algeria**	0.09 (0.07 to 0.11)	0.22 (0.19 to 0.25)	0.07 (0.06 to 0.09)
**American Samoa**	0.09 (0.06 to 0.12)	–0.13 (–0.20 to –0.07)	0.19 (0.16 to 0.21)
**Andorra**	0.16 (0.12 to 0.20)	–0.04 (–0.07 to –0.02)	–0.03 (–0.12 to 0.06)
**Angola**	0.22 (0.21 to 0.22)	0.27 (0.21 to 0.33)	0.06 (0.05 to 0.07)
**Antigua and Barbuda**	–0.03 (–0.05 to –0.01)	0.43 (0.40 to 0.47)	0.05 (0 to 0.11)
**Argentina**	0.13 (0.11 to 0.15)	0.06 (0.03 to 0.09)	0.01 (–0.03 to 0.05)
**Armenia**	0.15 (0.13 to 0.16)	1.01 (0.87 to 1.15)	0.16 (0.11 to 0.22)
**Australia**	0.01 (–0.01 to 0.03)	–0.25 (–0.31 to –0.18)	0.01 (–0.02 to 0.04)
**Austria**	0.37 (0.28 to 0.47)	–0.39 (–0.57 to –0.22)	0.08 (0 to 0.15)
**Azerbaijan**	0.02 (0.01 to 0.03)	0.28 (0.21 to 0.34)	0.15 (0.09 to 0.20)
**Bahamas**	0.05 (0.03 to 0.06)	0.36 (0.34 to 0.38)	0.05 (0 to 0.11)
**Bahrain**	–0.01 (–0.05 to 0.03)	0.32 (0.29 to 0.36)	0.12 (0.08 to 0.16)
**Bangladesh**	0.17 (0.14 to 0.19)	0.40 (0.35 to 0.45)	0.11 (0.08 to 0.14)
**Barbados**	0.07 (0.05 to 0.08)	0.44 (0.38 to 0.50)	0.05 (0 to 0.11)
**Belarus**	–0.02 (–0.04 to –0.01)	–0.40 (–0.51 to –0.30)	0.03 (–0.01 to 0.06)
**Belgium**	–0.03 (–0.07 to 0.01)	1.88 (1.06 to 2.71)	–0.12 (–0.24 to 0.01)
**Belize**	0.14 (0.12 to 0.16)	0.55 (0.50 to 0.60)	0.13 (0.07 to 0.20)
**Benin**	0.04 (0.03 to 0.04)	0.34 (0.27 to 0.42)	0.05 (0.03 to 0.06)
**Bermuda**	0.02 (0.01 to 0.03)	0.33 (0.32 to 0.35)	0.05 (0 to 0.10)
**Bhutan**	0.10 (0.08 to 0.11)	0.45 (0.41 to 0.49)	0.16 (0.14 to 0.17)
**Bolivia**	0.12 (0.09 to 0.15)	0.18 (0.13 to 0.23)	0.03 (–0.05 to 0.11)
**Bosnia and Herzegovina**	–0.01 (–0.02 to 0.01)	0.11 (0.08 to 0.14)	0.16 (0.07 to 0.25)
**Botswana**	0.69 (0.35 to 1.04)	0.13 (0.05 to 0.21)	0.10 (0.08 to 0.13)
**Brazil**	0.04 (–0.04 to 0.12)	–0.38 (–0.46 to –0.29)	–0.04 (–0.10 to 0.02)
**Brunei**	0.05 (0.01 to 0.09)	–0.12 (–0.18 to –0.06)	–0.11 (–0.15 to –0.06)
**Bulgaria**	–0.05 (–0.06 to –0.03)	–1.27 (–1.53 to –1.00)	0.07 (–0.02 to 0.16)
**Burkina Faso**	0.07 (0.06 to 0.08)	0.34 (0.29 to 0.39)	0.03 (0.01 to 0.04)
**Burundi**	–0.05 (–0.06 to –0.04)	0.08 (0.04 to 0.12)	–0.05 (–0.06 to –0.04)
**Cambodia**	–0.01 (–0.04 to 0.03)	0.12 (0.09 to 0.15)	0.11 (0.09 to 0.13)
**Cameroon**	0 (0 to 0.01)	0.31 (0.24 to 0.38)	0.07 (0.06 to 0.09)

Location	EAPC (95% CI)		
	Urinary tract infections	Urolithiasis	Benign prostatic hyperplasia
**Canada**	0.02 (0.01 to 0.04)	0.25 (0.22 to 0.28)	–0.02 (–0.08 to 0.04)
**Cape Verde**	–0.07 (–0.09 to –0.05)	0.41 (0.34 to 0.49)	0.07 (0.06 to 0.09)
**Central African Republic**	0.06 (0.03 to 0.08)	0.29 (0.23 to 0.34)	0.01 (0 to 0.03)
**Chad**	–0.07 (–0.1 to –0.04)	0.44 (0.36 to 0.53)	0.04 (0.02 to 0.06)
**Chile**	0.09 (–0.17 to 0.34)	–0.91 (–1.14 to –0.68)	–0.01 (–0.06 to 0.04)
**China**	–0.20 (–0.27 to –0.13)	–2.80 (–3.07 to –2.52)	–0.09 (–0.20 to 0.03)
**Colombia**	0.10 (0.09 to 0.11)	0.14 (0.10 to 0.17)	0.01 (–0.02 to 0.05)
**Comoros**	0.06 (0.05 to 0.07)	0.20 (0.17 to 0.23)	–0.04 (–0.04 to –0.03)
**Congo**	0.07 (0.06 to 0.09)	0.35 (0.28 to 0.41)	0 (–0.01 to 0.01)
**Cook Islands**	0.29 (0.27 to 0.30)	0.07 (0.03 to 0.10)	0.24 (0.20 to 0.27)
**Costa Rica**	0.08 (0.06 to 0.11)	0.01 (–0.02 to 0.04)	0.05 (0 to 0.10)
**Croatia**	0.61 (0.43 to 0.78)	0.22 (–0.07 to 0.50)	–0.08 (–0.18 to 0.02)
**Cuba**	–0.01 (–0.02 to 0)	0.95 (0.89 to 1.00)	0.03 (–0.03 to 0.08)
**Cyprus**	–0.41 (–0.66 to –0.17)	–1.38 (–2.08 to –0.68)	–0.07 (–0.14 to 0)
**Czech Republic**	0.21 (0.14 to 0.28)	0.44 (0.05 to 0.83)	0.08 (0.01 to 0.15)
**Democratic Republic of the Congo**	0.04 (0.02 to 0.05)	0.31 (0.25 to 0.37)	0 (–0.01 to 0.01)
**Denmark**	0.08 (0.05 to 0.11)	0.22 (0.17 to 0.26)	–0.05 (–0.15 to 0.05)
**Djibouti**	0.10 (0.08 to 0.11)	0.30 (0.25 to 0.34)	0.01 (0 to 0.02)
**Dominica**	–0.06 (–0.08 to –0.04)	0.41 (0.38 to 0.45)	0.06 (–0.01 to 0.12)
**Dominican Republic**	–0.06 (–0.07 to –0.04)	0.31 (0.27 to 0.34)	0.07 (0.02 to 0.12)
**Ecuador**	1.09 (0.95 to 1.24)	1.48 (1.26 to 1.70)	0.36 (0.11 to 0.62)
**Egypt**	0.09 (0.07 to 0.10)	0.23 (0.19 to 0.27)	0.14 (0.12 to 0.15)
**El Salvador**	0.20 (0.18 to 0.22)	0.03 (–0.01 to 0.06)	0.10 (0.07 to 0.14)
**Equatorial Guinea**	0.10 (0.06 to 0.15)	0.37 (0.31 to 0.44)	0.11 (0.08 to 0.13)
**Eritrea**	0.07 (0.06 to 0.08)	0.23 (0.20 to 0.26)	–0.03 (–0.04 to –0.02)
**Estonia**	–0.12 (–0.14 to –0.11)	–0.53 (–0.64 to –0.41)	0.08 (0.04 to 0.12)
**Eswatini**	0.01 (–0.02 to 0.03)	0.20 (0.17 to 0.24)	0.15 (0.12 to 0.18)
**Ethiopia**	0.12 (0.10 to 0.14)	–0.10 (–0.15 to –0.05)	–0.03 (–0.06 to –0.01)
**Fiji**	–0.01 (–0.02 to 0.01)	0.22 (0.18 to 0.26)	0.15 (0.10 to 0.20)
**Finland**	–0.01 (–0.09 to 0.08)	–0.60 (–1.33 to 0.14)	–0.01 (–0.08 to 0.06)
**France**	0.04 (0.03 to 0.06)	0.05 (0.02 to 0.08)	–0.02 (–0.10 to 0.07)
**Gabon**	0.17 (0.16 to 0.18)	0.35 (0.30 to 0.41)	0.03 (0.01 to 0.05)
**Gambia**	0.12 (0.10 to 0.13)	0.26 (0.18 to 0.33)	0.03 (0.01 to 0.04)

Location	EAPC (95% CI)		
	Urinary tract infections	Urolithiasis	Benign prostatic hyperplasia
**Georgia**	0.25 (0.16 to 0.34)	–0.83 (–0.99 to –0.67)	0.12 (0.03 to 0.22)
**Germany**	0.09 (0.07 to 0.12)	2.00 (1.39 to 2.61)	–0.02 (–0.11 to 0.07)
**Ghana**	0.13 (0.12 to 0.15)	0.34 (0.19 to 0.49)	0.10 (0.08 to 0.11)
**Greece**	0.03 (0 to 0.06)	0.06 (0.03 to 0.08)	–0.05 (–0.13 to 0.03)
**Greenland**	0.06 (0.04 to 0.08)	0.11 (0.08 to 0.15)	–0.07 (–0.13 to –0.02)
**Grenada**	–0.06 (–0.07 to –0.05)	1.31 (1.20 to 1.43)	0.14 (0.08 to 0.21)
**Guam**	0.06 (0.01 to 0.12)	–0.13 (–0.21 to –0.04)	0.20 (0.17 to 0.22)
**Guatemala**	0.18 (0.16 to 0.21)	–0.03 (–0.06 to –0.01)	0.17 (0.12 to 0.21)
**Guinea**	0.07 (0.06 to 0.08)	0.37 (0.29 to 0.45)	0.01 (0 to 0.03)
**Guinea-Bissau**	0.05 (0.03 to 0.08)	0.23 (0.14 to 0.31)	0.03 (0.02 to 0.05)
**Guyana**	0.06 (0.04 to 0.08)	0.68 (0.58 to 0.77)	0.11 (0.04 to 0.18)
**Haiti**	0.07 (0.06 to 0.09)	0.33 (0.29 to 0.36)	0.03 (–0.02 to 0.08)
**Honduras**	0.09 (0.08 to 0.10)	–0.01 (–0.14 to 0.13)	0.09 (0.05 to 0.12)
**Hungary**	–0.01 (–0.03 to 0.01)	–0.90 (–1.01 to –0.80)	0.08 (–0.01 to 0.16)
**Iceland**	–0.26 (–0.34 to –0.18)	–0.62 (–0.83 to –0.40)	–0.02 (–0.12 to 0.07)
**India**	0.37 (0.35 to 0.40)	0.71 (0.54 to 0.87)	0.31 (0.14 to 0.48)
**Indonesia**	–0.73 (–0.89 to –0.56)	–2.79 (–3.19 to –2.39)	–0.32 (–0.56 to –0.08)
**Iran**	0.07 (0.05 to 0.1)	0.22 (0.15 to 0.28)	0.08 (0.06 to 0.10)
**Iraq**	0.09 (0.06 to 0.11)	0.15 (0.12 to 0.18)	0.09 (0.09 to 0.10)
**Ireland**	0.07 (0.04 to 0.1)	0.04 (0.01 to 0.06)	–0.04 (–0.12 to 0.04)
**Israel**	0 (–0.01 to 0.01)	0.25 (0.23 to 0.27)	–0.01 (–0.1 to 0.08)
**Italy**	–2.61 (–3.09 to –2.12)	–0.57 (–0.65 to –0.49)	–0.10 (–0.18 to –0.01)
**Ivory Coast**	0.06 (0.05 to 0.07)	0.32 (0.24 to 0.40)	0.03 (0.01 to 0.04)
**Jamaica**	–0.03 (–0.04 to –0.02)	0.59 (0.56 to 0.62)	0.15 (0.09 to 0.21)
**Japan**	–0.06 (–0.16 to 0.05)	–0.39 (–0.49 to –0.29)	–0.07 (–0.12 to –0.02)
**Jordan**	0.46 (0.39 to 0.53)	2.10 (1.74 to 2.47)	0.18 (0.08 to 0.28)
**Kazakhstan**	0.04 (0.02 to 0.05)	–0.63 (–0.75 to –0.51)	0.11 (0.06 to 0.17)
**Kenya**	0.15 (0.11 to 0.19)	0.53 (0.36 to 0.69)	–0.02 (–0.03 to –0.01)
**Kiribati**	0.08 (0.05 to 0.11)	0.02 (0 to 0.04)	0.22 (0.17 to 0.27)
**Kuwait**	0.27 (0.20 to 0.34)	0.12 (0.10 to 0.14)	0.05 (0.01 to 0.09)
**Kyrgyzstan**	0.08 (0.05 to 0.1)	0.81 (0.49 to 1.13)	0.09 (0.01 to 0.16)
**Laos**	0.06 (0.04 to 0.08)	–0.20 (–0.26 to –0.14)	0.13 (0.11 to 0.15)
**Latvia**	0.39 (0.03 to 0.75)	–0.88 (–1.08 to –0.68)	0.09 (0.05 to 0.13)

Location	EAPC (95% CI)		
	Urinary tract infections	Urolithiasis	Benign prostatic hyperplasia
**Lebanon**	0.16 (0.15 to 0.17)	0.22 (0.19 to 0.25)	–0.01 (–0.02 to 0)
**Lesotho**	–0.02 (–0.05 to 0)	0.17 (0.12 to 0.23)	0.17 (0.14 to 0.20)
**Liberia**	0.06 (0.04 to 0.08)	0.34 (0.26 to 0.42)	0.03 (0.01 to 0.05)
**Libya**	0.09 (0.07 to 0.11)	0.16 (0.12 to 0.20)	0.06 (0.04 to 0.08)
**Lithuania**	0.12 (–0.38 to 0.61)	–1.33 (–1.49 to –1.17)	0.06 (0.03 to 0.10)
**Luxembourg**	–0.02 (–0.18 to 0.13)	0.06 (–0.62 to 0.74)	–0.03 (–0.12 to 0.05)
**Macedonia**	0.03 (0.02 to 0.04)	0.05 (0.02 to 0.08)	0.12 (0.04 to 0.21)
**Madagascar**	0.02 (0 to 0.03)	0.16 (0.12 to 0.20)	–0.01 (–0.02 to 0.01)
**Malawi**	0.13 (0.12 to 0.14)	0.20 (0.17 to 0.23)	–0.01 (–0.02 to 0.01)
**Malaysia**	0.05 (0.02 to 0.07)	0.33 (0.27 to 0.38)	0.11 (0.08 to 0.13)
**Maldives**	–0.18 (–0.28 to –0.07)	0.44 (0.32 to 0.57)	0.06 (0.04 to 0.08)
**Mali**	0.04 (0.03 to 0.05)	0.32 (0.26 to 0.39)	0.03 (0.01 to 0.04)
**Malta**	–0.54 (–0.8 to –0.27)	1.92 (1.28 to 2.56)	0 (–0.08 to 0.08)
**Marshall Islands**	0.07 (0.04 to 0.09)	0.07 (0.05 to 0.09)	0.19 (0.17 to 0.22)
**Mauritania**	0.11 (0.1 to 0.13)	0.33 (0.27 to 0.39)	0.02 (0.01 to 0.04)
**Mauritius**	0.16 (0.13 to 0.18)	0.22 (0.18 to 0.26)	0.42 (0.38 to 0.46)
**Mexico**	0.87 (0.52 to 1.23)	0.20 (–0.42 to 0.83)	0.03 (–0.01 to 0.07)
**Micronesia**	0.06 (0.01 to 0.10)	0.14 (0.12 to 0.17)	0.28 (0.24 to 0.31)
**Moldova**	–0.04 (–0.05 to –0.03)	0.31 (0.24 to 0.38)	0.04 (0 to 0.07)
**Monaco**	0.04 (0.03 to 0.06)	0.09 (0.07 to 0.11)	–0.03 (–0.11 to 0.05)
**Mongolia**	0.08 (0.07 to 0.1)	0.19 (0.12 to 0.26)	0.12 (0.06 to 0.18)
**Montenegro**	0 (–0.01 to 0.01)	0.03 (0 to 0.06)	0.10 (0.02 to 0.19)
**Morocco**	0.08 (0.07 to 0.09)	0.21 (0.17 to 0.25)	0.09 (0.08 to 0.10)
**Mozambique**	0.08 (0.06 to 0.09)	0.37 (0.34 to 0.4)	0 (–0.01 to 0.01)
**Myanmar**	0.13 (0.10 to 0.17)	–0.35 (–0.44 to –0.26)	0.13 (0.11 to 0.15)
**Namibia**	0.13 (0.10 to 0.16)	0.15 (0.09 to 0.21)	0.01 (–0.03 to 0.04)
**Nauru**	0.20 (0.18 to 0.21)	0.03 (–0.02 to 0.07)	0.21 (0.19 to 0.23)
**Nepal**	–0.29 (–0.39 to –0.19)	0.36 (0.30 to 0.42)	0.05 (–0.02 to 0.11)
**Netherlands**	0.06 (0.04 to 0.08)	–0.23 (–0.27 to –0.20)	–0.02 (–0.1 to 0.07)
**New Zealand**	0.47 (0.39 to 0.54)	–0.83 (–1.05 to –0.61)	–0.3 (–0.38 to –0.22)
**Nicaragua**	0.06 (0.04 to 0.07)	0.11 (0.07 to 0.14)	0.08 (0.04 to 0.11)
**Niger**	0.12 (0.11 to 0.13)	0.29 (0.21 to 0.37)	0.04 (0.03 to 0.06)
**Nigeria**	0.27 (0.24 to 0.29)	0.18 (0.10 to 0.25)	0.02 (0.01 to 0.03)

Location	EAPC (95% CI)		
	Urinary tract infections	Urolithiasis	Benign prostatic hyperplasia
**Niue**	0 (–0.02 to 0.01)	0.24 (0.22 to 0.26)	0.22 (0.19 to 0.25)
**North Korea**	–0.24 (–0.25 to –0.23)	0.13 (0.10 to 0.16)	0.13 (0.10 to 0.15)
**Northern Mariana Islands**	0.09 (–0.01 to 0.19)	–0.02 (–0.05 to 0.02)	0.19 (0.15 to 0.22)
**Norway**	–0.15 (–0.23 to –0.06)	0.64 (0.42 to 0.86)	–0.01 (–0.07 to 0.06)
**Oman**	–0.03 (–0.13 to 0.08)	0.31 (0.24 to 0.38)	0.16 (0.15 to 0.17)
**Pakistan**	0.19 (0.18 to 0.21)	0.31 (0.22 to 0.41)	0.28 (0.26 to 0.3)
**Palau**	–0.15 (–0.22 to –0.08)	0.23 (0.18 to 0.28)	0.23 (0.20 to 0.27)
**Palestine**	0.02 (–0.01 to 0.05)	0.28 (0.25 to 0.31)	0.13 (0.11 to 0.14)
**Panama**	0.04 (0.02 to 0.05)	0.02 (–0.01 to 0.05)	0.09 (0.06 to 0.13)
**Papua New Guinea**	–0.01 (–0.02 to 0)	0.16 (0.11 to 0.21)	0.19 (0.17 to 0.22)
**Paraguay**	–0.01 (–0.04 to 0.01)	0.08 (0.06 to 0.10)	0.20 (0.17 to 0.22)
**Peru**	0.16 (0.13 to 0.18)	0.14 (0.09 to 0.18)	0.05 (–0.03 to 0.13)
**Philippines**	0.18 (0.13 to 0.24)	0.40 (0.24 to 0.56)	0.07 (0.05 to 0.08)
**Poland**	–1.31 (–1.51 to –1.12)	–3.87 (–4.47 to –3.27)	–0.68 (–1.00 to –0.36)
**Portugal**	–0.33 (–0.41 to –0.26)	0.30 (0.26 to 0.35)	–0.02 (–0.11 to 0.07)
**Puerto Rico**	0.05 (0.03 to 0.07)	0.29 (0.26 to 0.31)	0.10 (0.04 to 0.17)
**Qatar**	–0.46 (–0.82 to –0.11)	0.38 (0.35 to 0.42)	0.07 (0.03 to 0.11)
**Romania**	0.27 (0.19 to 0.34)	2.01 (1.37 to 2.66)	0.08 (0.01 to 0.15)
**Russia**	0.06 (0.05 to 0.08)	–0.62 (–0.79 to –0.46)	0.04 (0 to 0.07)
**Rwanda**	0.14 (0.12 to 0.16)	0.13 (0.09 to 0.18)	–0.05 (–0.07 to –0.04)
**Saint Kitts and Nevis**	–0.06 (–0.08 to –0.05)	0.31 (0.29 to 0.32)	0.08 (0.02 to 0.14)
**Saint Lucia**	–0.02 (–0.04 to 0)	0.47 (0.45 to 0.49)	–0.01 (–0.07 to 0.05)
**Saint Vincent and the Grenadines**	–0.03 (–0.05 to 0)	0.65 (0.61 to 0.69)	0.11 (0.05 to 0.17)
**Samoa**	0.03 (0 to 0.06)	0.13 (0.10 to 0.16)	0.20 (0.16 to 0.23)
**San Marino**	0.18 (0.16 to 0.20)	–0.06 (–0.08 to –0.05)	–0.02 (–0.11 to 0.07)
**Sao Tome and Principe**	0.04 (0.02 to 0.05)	0.36 (0.30 to 0.42)	0.03 (0.02 to 0.05)
**Saudi Arabia**	0.11 (0.09 to 0.14)	0.22 (0.19 to 0.26)	0.03 (0.01 to 0.05)
**Senegal**	0.05 (0.04 to 0.05)	0.32 (0.25 to 0.39)	0.04 (0.03 to 0.06)
**Serbia**	–0.08 (–0.1 to –0.06)	1.63 (1.13 to 2.14)	0.10 (0.03 to 0.18)
**Seychelles**	–0.01 (–0.06 to 0.04)	0.47 (0.43 to 0.52)	0.12 (0.10 to 0.13)
**Sierra Leone**	–0.01 (–0.03 to 0.01)	0.36 (0.28 to 0.44)	0.03 (0.02 to 0.05)
**Singapore**	0 (–0.02 to 0.02)	–0.02 (–0.09 to 0.04)	–0.09 (–0.13 to –0.05)
**Slovakia**	0.18 (–0.06 to 0.42)	0.23 (–0.56 to 1.04)	0.02 (–0.04 to 0.08)

Location	EAPC (95% CI)		
	Urinary tract infections	Urolithiasis	Benign prostatic hyperplasia
**Slovenia**	–0.07 (–0.11 to –0.02)	0.70 (0.20 to 1.19)	0.03 (–0.04 to 0.10)
**Solomon Islands**	0.12 (0.1 to 0.14)	0.01 (–0.02 to 0.04)	0.28 (0.25 to 0.31)
**Somalia**	0.04 (0.03 to 0.05)	0.13 (0.11 to 0.16)	–0.01 (–0.03 to 0)
**South Africa**	–0.02 (–0.04 to 0.01)	0.12 (0.07 to 0.17)	0.11 (0.08 to 0.14)
**South Korea**	0.01 (0 to 0.03)	0.10 (0.06 to 0.13)	0.06 (0 to 0.12)
**South Sudan**	0.21 (0.19 to 0.23)	0.26 (0.22 to 0.31)	0.01 (–0.01 to 0.02)
**Spain**	0.01 (–0.02 to 0.04)	0.12 (0.1 to 0.14)	–0.03 (–0.13 to 0.06)
**Sri Lanka**	0.28 (0.25 to 0.31)	0.09 (0.02 to 0.15)	0.20 (0.18 to 0.22)
**Sudan**	0.07 (0.05 to 0.10)	0.22 (0.18 to 0.26)	0.07 (0.06 to 0.09)
**Suriname**	0.08 (0.05 to 0.10)	0.88 (0.76 to 0.99)	0.15 (0.10 to 0.21)
**Sweden**	–0.02 (–0.08 to 0.04)	–0.14 (–0.42 to 0.13)	–0.15 (–0.24 to –0.06)
**Switzerland**	–0.12 (–0.49 to 0.26)	0.24 (–0.15 to 0.64)	–0.04 (–0.11 to 0.03)
**Syria**	0.18 (0.13 to 0.24)	0.21 (0.18 to 0.23)	0.03 (0.02 to 0.05)
**Tajikistan**	0.03 (0.01 to 0.05)	0.29 (0.26 to 0.33)	0.12 (0.06 to 0.17)
**Tanzania**	0.04 (0.03 to 0.06)	0.23 (0.18 to 0.28)	0 (–0.01 to 0.02)
**Thailand**	0.05 (0.01 to 0.10)	–0.76 (–0.93 to –0.59)	–0.02 (–0.03 to –0.02)
**Timor-Leste**	0.09 (0.07 to 0.11)	0.16 (0.10 to 0.22)	0.11 (0.09 to 0.12)
**Togo**	0.11 (0.10 to 0.12)	0.25 (0.17 to 0.34)	0.03 (0.01 to 0.05)
**Tokelau**	–0.12 (–0.16 to –0.08)	0.35 (0.31 to 0.40)	0.24 (0.21 to 0.27)
**Tonga**	0.04 (0.01 to 0.06)	0.24 (0.21 to 0.26)	0.20 (0.16 to 0.23)
**Trinidad and Tobago**	0.04 (0.02 to 0.05)	1.52 (1.30 to 1.74)	0.05 (0 to 0.10)
**Tunisia**	0.09 (0.08 to 0.10)	0.20 (0.17 to 0.23)	0.02 (0.01 to 0.04)
**Turkey**	0.38 (0.30 to 0.47)	0.34 (0.31 to 0.36)	–0.01 (–0.02 to 0.01)
**Turkmenistan**	0.13 (0.09 to 0.17)	0.31 (0.23 to 0.39)	0.13 (0.08 to 0.18)
**Tuvalu**	–0.28 (–0.30 to –0.26)	0.35 (0.31 to 0.40)	0.22 (0.20 to 0.25)
**Uganda**	0.12 (0.11 to 0.13)	0.21 (0.17 to 0.25)	–0.03 (–0.04 to –0.02)
**UK**	0.35 (–0.39 to 1.09)	0.83 (0.61 to 1.05)	0.02 (–0.08 to 0.13)
**Ukraine**	–0.09 (–0.12 to –0.06)	–0.94 (–1.10 to –0.78)	0.02 (–0.01 to 0.06)
**United Arab Emirates**	–0.06 (–0.14 to 0.02)	0.28 (0.25 to 0.31)	0.06 (0.04 to 0.08)
**Uruguay**	0.11 (0.09 to 0.13)	0.10 (0.07 to 0.13)	0 (–0.04 to 0.04)
**USA**	–0.34 (–0.4 to –0.27)	–2.25 (–2.59 to –1.91)	0.13 (0.07 to 0.20)
**Uzbekistan**	0.03 (0.01 to 0.06)	0.21 (0.15 to 0.26)	0.15 (0.09 to 0.20)
**Vanuatu**	0.05 (0.04 to 0.07)	0.17 (0.11 to 0.23)	0.27 (0.24 to 0.30)

Location	EAPC (95% CI)		
	Urinary tract infections	Urolithiasis	Benign prostatic hyperplasia
**Venezuela**	0.01 (0 to 0.02)	0.02 (–0.07 to 0.11)	0.06 (0.02 to 0.09)
**Vietnam**	–0.08 (–0.11 to –0.05)	1.85 (1.55 to 2.15)	0.26 (0.18 to 0.34)
**Virgin Islands**	0.06 (0.04 to 0.08)	1.19 (0.93 to 1.45)	0.08 (0.02 to 0.13)
**Yemen**	0.10 (0.08 to 0.12)	0.22 (0.17 to 0.26)	0.07 (0.05 to 0.09)
**Zambia**	0.08 (0.07 to 0.09)	0.05 (0.01 to 0.10)	0.02 (0.01 to 0.04)
**Zimbabwe**	0 (–0.03 to 0.03)	0 (–0.02 to 0.02)	0.09 (0.06 to 0.11)

EAPC estimated annual percentage change; *ASIR* age-standardized incidence rate; *CI* confidence interval

Table S6 EAPC of ASMR for urinary tract infections and urolithiasis in 203 countries and territories from 1990 to 2019

Location	EAPC (95% CI)	
	Urinary tract infections	Urolithiasis
**Afghanistan**	0.95 (0.83 to 1.07)	2.43 (2.13 to 2.73)
**Albania**	–3.50 (–4.13 to –2.86)	–7.30 (–8.72 to –5.85)
**Algeria**	0.52 (0.27 to 0.78)	1.46 (1.07 to 1.86)
**American Samoa**	–0.85 (–1.21 to –0.49)	–3.87 (–5.23 to –2.49)
**Andorra**	–0.28 (–0.45 to –0.11)	–1.02 (–1.35 to –0.69)
**Angola**	–1.00 (–1.15 to –0.85)	–2.06 (–2.27 to –1.86)
**Antigua and Barbuda**	3.99 (3.53 to 4.44)	2.46 (2.17 to 2.74)
**Argentina**	7.27 (6.30 to 8.25)	3.57 (2.94 to 4.21)
**Armenia**	8.48 (7.28 to 9.69)	4.11 (3.53 to 4.69)
**Australia**	1.08 (0.59 to 1.58)	–2.56 (–3.13 to –1.98)
**Austria**	–0.58 (–1.46 to 0.30)	–3.47 (–4.78 to –2.13)
**Azerbaijan**	3.59 (2.09 to 5.11)	4.41 (3.38 to 5.45)
**Bahamas**	2.52 (2.11 to 2.93)	1.51 (1.21 to 1.82)
**Bahrain**	2.70 (1.87 to 3.53)	2.63 (1.34 to 3.94)
**Bangladesh**	–0.21 (–0.45 to 0.04)	–2.07 (–2.35 to –1.79)
**Barbados**	3.72 (3.03 to 4.41)	1.47 (1.22 to 1.72)
**Belarus**	–0.52 (–1.06 to 0.02)	–1.13 (–1.33 to –0.94)
**Belgium**	4.90 (4.27 to 5.53)	2.34 (1.86 to 2.83)
**Belize**	3.78 (2.93 to 4.63)	2.70 (1.88 to 3.52)
**Benin**	–1.03 (–1.12 to –0.94)	–1.96 (–2.08 to –1.83)
**Bermuda**	1.90 (1.50 to 2.29)	0.71 (0.30 to 1.11)
**Bhutan**	0.84 (0.77 to 0.91)	–1.47 (–1.56 to –1.38)
**Bolivia**	1.18 (1.09 to 1.27)	–0.57 (–0.71 to –0.43)
**Bosnia and Herzegovina**	–5.85 (–6.75 to –4.95)	–5.90 (–7.07 to –4.71)
**Botswana**	–0.04 (–0.35 to 0.27)	–0.15 (–0.53 to 0.24)
**Brazil**	3.48 (3.12 to 3.84)	4.00 (3.76 to 4.24)
**Brunei**	0.91 (0.65 to 1.17)	–0.28 (–0.57 to 0)
**Bulgaria**	–5.11 (–6.02 to –4.2)	–10.59 (–12.10 to –9.06)
**Burkina Faso**	–0.17 (–0.35 to 0.01)	–0.56 (–0.69 to –0.42)
**Burundi**	–0.14 (–0.30 to 0.01)	–0.81 (–0.95 to –0.68)
**Cambodia**	–0.06 (–0.13 to 0.01)	–1.18 (–1.31 to –1.06)
**Cameroon**	–1.12 (–1.25 to –0.98)	–2.45 (–2.65 to –2.24)

Location	EAPC (95% CI)	
	Urinary tract infections	Urolithiasis
Canada	1.69 (1.30 to 2.07)	2.03 (1.55 to 2.51)
Cape Verde	0.77 (0.50 to 1.05)	–0.71 (–1.18 to –0.24)
Central African Republic	–0.18 (–0.26 to –0.10)	0.17 (0 to 0.34)
Chad	–1.12 (–1.30 to –0.93)	–1.94 (–2.09 to –1.78)
Chile	0.62 (0.30 to 0.94)	–1.30 (–1.74 to –0.85)
China	–2.75 (–3.16 to –2.34)	–5.10 (–5.40 to –4.79)
Colombia	2.44 (1.78 to 3.10)	2.20 (1.32 to 3.08)
Comoros	0.09 (–0.03 to 0.21)	–0.15 (–0.26 to –0.05)
Congo	–0.82 (–1.02 to –0.63)	–1.49 (–1.70 to –1.28)
Cook Islands	–0.77 (–1.01 to –0.53)	–2.00 (–2.31 to –1.68)
Costa Rica	4.06 (3.50 to 4.63)	7.18 (6.30 to 8.06)
Croatia	2.24 (1.84 to 2.65)	2.40 (0.99 to 3.82)
Cuba	3.90 (3.59 to 4.21)	1.60 (1.27 to 1.93)
Cyprus	0.08 (–0.36 to 0.53)	–2.87 (–3.16 to –2.58)
Czech Republic	–2.31 (–3.07 to –1.56)	–7.19 (–8.22 to –6.14)
Democratic Republic of the Congo	–0.29 (–0.35 to –0.23)	–0.37 (–0.53 to –0.22)
Denmark	2.16 (1.46 to 2.87)	0.70 (0.26 to 1.15)
Djibouti	0.98 (0.74 to 1.21)	0.43 (0.24 to 0.61)
Dominica	3.62 (3.24 to 4.01)	2.83 (2.60 to 3.06)
Dominican Republic	1.67 (1.44 to 1.91)	–0.10 (–0.87 to 0.67)
Ecuador	3.02 (2.54 to 3.51)	1.72 (1.22 to 2.22)
Egypt	–0.12 (–0.22 to –0.02)	1.49 (1.23 to 1.76)
El Salvador	–0.43 (–0.65 to –0.22)	–0.87 (–1.38 to –0.36)
Equatorial Guinea	–0.34 (–0.67 to –0.01)	–2.03 (–2.49 to –1.57)
Eritrea	0.42 (0.24 to 0.59)	0.51 (0.36 to 0.65)
Estonia	–4.42 (–4.83 to –4.01)	–6.47 (–7.24 to –5.69)
Eswatini	–0.10 (–0.68 to 0.49)	0.30 (–0.47 to 1.08)
Ethiopia	–2.30 (–2.44 to –2.16)	–2.34 (–2.50 to –2.19)
Fiji	1.55 (1.33 to 1.78)	3.88 (3.11 to 4.66)
Finland	–4.94 (–5.63 to –4.25)	–1.2 (–1.64 to –0.76)
France	–0.27 (–0.52 to –0.02)	–1.64 (–1.79 to –1.49)
Gabon	0.23 (0.08 to 0.38)	–0.64 (–0.84 to –0.44)
Gambia	–0.04 (–0.17 to 0.08)	–1.21 (–1.36 to –1.06)

Location	EAPC (95% CI)	
	Urinary tract infections	Urolithiasis
**Georgia**	4.07 (3.2 to 4.94)	7.12 (5.62 to 8.64)
**Germany**	3.53 (3.22 to 3.85)	–0.39 (–1.14 to 0.37)
**Ghana**	0.37 (0.12 to 0.62)	0.31 (0.13 to 0.50)
**Greece**	–0.31 (–1.77 to 1.16)	0.46 (–0.55 to 1.48)
**Greenland**	–0.71 (–1.03 to –0.38)	–3.90 (–4.65 to –3.14)
**Grenada**	4.11 (3.58 to 4.63)	2.92 (2.49 to 3.35)
**Guam**	–2.63 (–3.25 to –2.00)	–8.97 (–10.48 to –7.44)
**Guatemala**	3.18 (2.84 to 3.52)	–1.44 (–1.83 to –1.05)
**Guinea**	–0.85 (–0.93 to –0.78)	–1.25 (–1.36 to –1.14)
**Guinea–Bissau**	–1.59 (–1.70 to –1.49)	–2.05 (–2.16 to –1.94)
**Guyana**	3.14 (2.63 to 3.65)	1.58 (1.17 to 1.99)
**Haiti**	1.25 (1.12 to 1.39)	0.57 (0.43 to 0.71)
**Honduras**	1.19 (0.96 to 1.42)	–0.25 (–0.48 to –0.03)
**Hungary**	–2.37 (–2.83 to –1.90)	–4.42 (–5.21 to –3.61)
**Iceland**	1.12 (0.68 to 1.57)	–1.15 (–1.39 to –0.91)
**India**	–0.44 (–0.56 to –0.31)	–3.02 (–3.24 to –2.81)
**Indonesia**	0.49 (0.42 to 0.56)	–0.26 (–0.34 to –0.19)
**Iran**	1.26 (0.77 to 1.76)	1.87 (0.79 to 2.95)
**Iraq**	–2.77 (–3.14 to –2.40)	–4.80 (–5.44 to –4.16)
**Ireland**	–0.36 (–0.57 to –0.15)	–1.20 (–1.77 to –0.62)
**Israel**	2.81 (1.60 to 4.03)	1.28 (0.20 to 2.38)
**Italy**	1.58 (1.03 to 2.12)	–3.53 (–4.25 to –2.80)
**Ivory Coast**	–1.21 (–1.34 to –1.07)	–1.72 (–1.89 to –1.56)
**Jamaica**	3.04 (2.26 to 3.83)	6.74 (6.04 to 7.46)
**Japan**	1.25 (0.88 to 1.62)	3.23 (2.92 to 3.54)
**Jordan**	1.76 (1.45 to 2.07)	–2.06 (–2.39 to –1.74)
**Kazakhstan**	0.14 (–0.26 to 0.54)	0.28 (–0.09 to 0.64)
**Kenya**	0.97 (0.83 to 1.10)	1.18 (1.08 to 1.29)
**Kiribati**	–0.38 (–0.43 to –0.33)	–1.21 (–1.29 to –1.13)
**Kuwait**	5.79 (5.08 to 6.51)	4.53 (3.75 to 5.31)
**Kyrgyzstan**	1.43 (0.37 to 2.50)	–2.79 (–3.46 to –2.11)
**Laos**	–0.94 (–1.1 to –0.77)	–2.33 (–2.57 to –2.10)
**Latvia**	–1.89 (–2.42 to –1.37)	–2.65 (–3.09 to –2.22)

Location	EAPC (95% CI)	
	Urinary tract infections	Urolithiasis
**Lebanon**	0.13 (–0.06 to 0.32)	2.16 (1.71 to 2.62)
**Lesotho**	2.16 (1.85 to 2.48)	2.57 (2.13 to 3.02)
**Liberia**	–1.45 (–1.66 to –1.23)	–2.15 (–2.41 to –1.88)
**Libya**	1.81 (1.49 to 2.14)	3.60 (3.11 to 4.09)
**Lithuania**	–0.28 (–0.69 to 0.13)	–4.01 (–4.42 to –3.60)
**Luxembourg**	1.51 (1.24 to 1.78)	–1.88 (–2.10 to –1.66)
**Macedonia**	–2.38 (–3.09 to –1.67)	–2.85 (–3.73 to –1.97)
**Madagascar**	–0.10 (–0.22 to 0.02)	–0.15 (–0.24 to –0.07)
**Malawi**	–0.12 (–0.21 to –0.04)	–0.02 (–0.08 to 0.04)
**Malaysia**	1.61 (1.34 to 1.89)	0.29 (0.07 to 0.50)
**Maldives**	–1.84 (–2.01 to –1.67)	–4.42 (–4.67 to –4.16)
**Mali**	–1.15 (–1.31 to –0.98)	–2.33 (–2.53 to –2.14)
**Malta**	1.20 (0.96 to 1.43)	–1.85 (–2.18 to –1.51)
**Marshall Islands**	–0.37 (–0.46 to –0.29)	–1.02 (–1.10 to –0.94)
**Mauritania**	–1.87 (–1.94 to –1.80)	–3.42 (–3.58 to –3.26)
**Mauritius**	4.63 (3.94 to 5.31)	–1.51 (–2.6 to –0.41)
**Mexico**	1.87 (1.22 to 2.52)	–0.27 (–0.57 to 0.03)
**Micronesia**	–0.43 (–0.58 to –0.28)	–1.53 (–1.64 to –1.42)
**Moldova**	–1.02 (–1.32 to –0.71)	–0.24 (–0.94 to 0.46)
**Monaco**	1.15 (0.88 to 1.41)	–0.14 (–0.28 to 0)
**Mongolia**	–4.33 (–4.87 to –3.78)	–5.97 (–6.71 to –5.23)
**Montenegro**	0.10 (–0.01 to 0.2)	0.40 (0.27 to 0.53)
**Morocco**	1.86 (1.66 to 2.07)	3.07 (2.68 to 3.46)
**Mozambique**	0.85 (0.76 to 0.95)	0.39 (0.28 to 0.50)
**Myanmar**	–0.71 (–0.92 to –0.50)	–1.76 (–2.05 to –1.47)
**Namibia**	–0.91 (–1.31 to –0.51)	–1.34 (–1.81 to –0.86)
**Nauru**	–0.77 (–1.04 to –0.49)	–1.39 (–1.48 to –1.29)
**Nepal**	1.34 (1.06 to 1.62)	–0.74 (–1.14 to –0.35)
**Netherlands**	0.12 (–0.45 to 0.70)	–3.08 (–3.44 to –2.71)
**New Zealand**	0.04 (–0.61 to 0.69)	–0.90 (–1.43 to –0.36)
**Nicaragua**	1.37 (1.10 to 1.65)	1.22 (0.58 to 1.87)
**Niger**	–1.54 (–1.67 to –1.41)	–2.61 (–2.80 to –2.41)
**Nigeria**	–1.08 (–1.21 to –0.96)	–2.35 (–2.54 to –2.15)

Location	EAPC (95% CI)	
	Urinary tract infections	Urolithiasis
**Niue**	–1.35 (–1.48 to –1.22)	–1.99 (–2.10 to –1.88)
**North Korea**	–1.08 (–1.23 to –0.93)	–1.02 (–1.27 to –0.78)
**Northern Mariana Islands**	–1.60 (–1.86 to –1.34)	–2.78 (–3.6 to –1.95)
**Norway**	0.91 (0.61 to 1.20)	–2.36 (–2.83 to –1.88)
**Oman**	3.76 (2.87 to 4.66)	4.16 (3.44 to 4.89)
**Pakistan**	1.08 (0.78 to 1.39)	–0.48 (–0.8 to –0.16)
**Palau**	–0.8 (–0.86 to –0.74)	–1.42 (–1.50 to –1.34)
**Palestine**	–1.11 (–1.6 to –0.61)	–1.53 (–2.23 to –0.83)
**Panama**	4.24 (3.75 to 4.73)	2.16 (1.03 to 3.30)
**Papua New Guinea**	–0.46 (–0.50 to –0.43)	–1.25 (–1.33 to –1.18)
**Paraguay**	3.83 (3.27 to 4.39)	3.00 (2.28 to 3.73)
**Peru**	1.43 (0.68 to 2.18)	–0.22 (–0.79 to 0.35)
**Philippines**	–0.11 (–0.28 to 0.05)	–1.04 (–1.17 to –0.91)
**Poland**	0.36 (–0.89 to 1.62)	–8.98 (–10.25 to –7.69)
**Portugal**	8.28 (7.36 to 9.20)	1.12 (0.03 to 2.23)
**Puerto Rico**	2.42 (1.50 to 3.35)	3.67 (2.78 to 4.57)
**Qatar**	0.68 (0.34 to 1.02)	0.42 (0 to 0.84)
**Romania**	–1.82 (–2.13 to –1.50)	–0.30 (–0.94 to 0.34)
**Russia**	–0.44 (–0.87 to 0)	–0.59 (–1.15 to –0.03)
**Rwanda**	–0.6 (–0.76 to –0.43)	–1.02 (–1.22 to –0.81)
**Saint Kitts and Nevis**	2.84 (2.43 to 3.24)	0.77 (0.45 to 1.10)
**Saint Lucia**	2.83 (2.44 to 3.22)	0.96 (0.68 to 1.24)
**Saint Vincent and the Grenadines**	3.05 (2.85 to 3.25)	1.25 (0.83 to 1.67)
**Samoa**	–1.39 (–1.49 to –1.28)	–2.19 (–2.33 to –2.05)
**San Marino**	2.26 (1.92 to 2.60)	0.42 (0.17 to 0.68)
**Sao Tome and Principe**	0.51 (0.27 to 0.75)	–1.41 (–1.66 to –1.15)
**Saudi Arabia**	–0.09 (–0.52 to 0.34)	1.35 (0.99 to 1.71)
**Senegal**	–1.10 (–1.21 to –0.99)	–2.33 (–2.46 to –2.2)
**Serbia**	–2.64 (–2.97 to –2.31)	–1.05 (–1.22 to –0.88)
**Seychelles**	3.21 (2.87 to 3.54)	1.93 (1.50 to 2.36)
**Sierra Leone**	–0.58 (–0.66 to –0.49)	–1.31 (–1.44 to –1.18)
**Singapore**	–1.49 (–2.01 to –0.97)	–5.19 (–6.25 to –4.12)
**Slovakia**	–0.17 (–0.68 to 0.33)	–4.39 (–4.74 to –4.03)

Location	EAPC (95% CI)	
	Urinary tract infections	Urolithiasis
**Slovenia**	–4.12 (–4.62 to –3.62)	–5.53 (–5.93 to –5.13)
**Solomon Islands**	–0.19 (–0.29 to –0.09)	–0.86 (–0.98 to –0.75)
**Somalia**	0.55 (0.46 to 0.64)	0.79 (0.68 to 0.90)
**South Africa**	–0.39 (–1.06 to 0.28)	–0.49 (–1.2 to 0.22)
**South Korea**	0.53 (–0.15 to 1.20)	–1.11 (–1.71 to –0.51)
**South Sudan**	0.02 (–0.01 to 0.05)	–0.26 (–0.34 to –0.18)
**Spain**	2.95 (2.55 to 3.35)	–1.39 (–1.71 to –1.08)
**Sri Lanka**	3.69 (2.68 to 4.71)	0.59 (–0.13 to 1.31)
**Sudan**	0.98 (0.71 to 1.25)	2.55 (2.14 to 2.97)
**Suriname**	3.08 (2.60 to 3.57)	2.19 (1.68 to 2.70)
**Sweden**	–1.46 (–1.84 to –1.09)	–0.57 (–1.30 to 0.16)
**Switzerland**	2.32 (1.66 to 3.00)	–2.65 (–2.95 to –2.35)
**Syria**	–3.46 (–4.09 to –2.83)	–3.76 (–4.29 to –3.23)
**Tajikistan**	2.87 (2.46 to 3.28)	2.07 (1.84 to 2.30)
**Tanzania**	0.63 (0.49 to 0.77)	0.04 (–0.05 to 0.14)
**Thailand**	–2.89 (–3.40 to –2.39)	–2.16 (–2.80 to –1.52)
**Timor–Leste**	0.23 (0.06 to 0.39)	–0.46 (–0.63 to –0.29)
**Togo**	–1.20 (–1.34 to –1.06)	–1.96 (–2.12 to –1.80)
**Tokelau**	–1.28 (–1.33 to –1.23)	–2.65 (–2.77 to –2.52)
**Tonga**	–0.20 (–0.48 to 0.07)	–0.99 (–1.2 to –0.78)
**Trinidad and Tobago**	1.95 (1.51 to 2.40)	3.58 (2.84 to 4.32)
**Tunisia**	0.81 (0.65 to 0.97)	2.00 (1.66 to 2.34)
**Turkey**	4.02 (3.12 to 4.94)	1.24 (0.49 to 1.99)
**Turkmenistan**	5.58 (4.64 to 6.54)	4.77 (4.32 to 5.23)
**Tuvalu**	–1.38 (–1.48 to –1.28)	–2.21 (–2.28 to –2.14)
**Uganda**	0.34 (0.15 to 0.53)	0.35 (0.16 to 0.55)
**UK**	3.96 (3.06 to 4.86)	0.69 (0.37 to 1.02)
**Ukraine**	–0.36 (–0.68 to –0.04)	–1.27 (–1.61 to –0.93)
**United Arab Emirates**	0.04 (–0.52 to 0.61)	2.86 (1.99 to 3.73)
**Uruguay**	5.72 (4.98 to 6.46)	2.84 (2.42 to 3.27)
**USA**	–0.16 (–0.44 to 0.11)	0.65 (0.30 to 1.01)
**Uzbekistan**	2.54 (1.97 to 3.11)	4.27 (3.82 to 4.72)
**Vanuatu**	–0.21 (–0.30 to –0.11)	–0.84 (–1.01 to –0.68)

Location	EAPC (95% CI)	
	Urinary tract infections	Urolithiasis
**Venezuela**	2.69 (1.94 to 3.45)	0.48 (–0.37 to 1.35)
**Vietnam**	–0.53 (–1.03 to –0.03)	–1.23 (–1.75 to –0.71)
**Virgin Islands**	1.85 (1.60 to 2.11)	1.67 (1.43 to 1.92)
**Yemen**	1.27 (1.02 to 1.53)	2.78 (2.39 to 3.17)
**Zambia**	–0.12 (–0.32 to 0.08)	–0.29 (–0.37 to –0.20)
**Zimbabwe**	1.20 (0.78 to 1.62)	0.98 (0.40 to 1.56)

EAPC estimated annual percentage change; *ASMR* age-standardized mortality rate; *CI* confidence interval

Table S7 EAPC of ASDR for the three urologic benign diseases in 203 countries and territories from 1990 to 2019

Location	EAPC (95% CI)		
	Urinary tract infections	Urolithiasis	Benign prostatic hyperplasia
**Afghanistan**	0.38(0.26 to 0.50)	0.76(0.68 to 0.85)	0.08(0.06 to 0.09)
**Albania**	–3.13(–3.68 to –2.59)	–1.48(–1.93 to –1.04)	0.07(0.03 to 0.10)
**Algeria**	–0.12(–0.30 to 0.07)	0.26(0.21 to 0.30)	0.07(0.06 to 0.08)
**American Samoa**	–0.85(–1.20 to –0.51)	–2.32(–3.02 to –1.61)	0.21(0.19 to 0.24)
**Andorra**	–0.50(–0.64 to –0.35)	–0.59(–0.75 to –0.44)	–0.04(–0.07 to –0.01)
**Angola**	–1.32(–1.47 to –1.17)	–1.65(–1.78 to –1.52)	0.07(0.06 to 0.08)
**Antigua and Barbuda**	3.21(2.83 to 3.58)	1.14(1.04 to 1.24)	0.02(–0.01 to 0.06)
**Argentina**	6.18(5.41 to 6.95)	0.31(0.26 to 0.36)	0.01(–0.01 to 0.03)
**Armenia**	6.35(5.37 to 7.35)	2.46(2.09 to 2.83)	0.15(0.11 to 0.19)
**Australia**	0.32(–0.02 to 0.66)	–1.18(–1.44 to –0.92)	0.03(0.02 to 0.04)
**Austria**	–1.07(–1.72 to –0.41)	–1.18(–1.51 to –0.84)	0.10(0.08 to 0.12)
**Azerbaijan**	2.07(0.82 to 3.33)	0.89(0.71 to 1.08)	0.11(0.07 to 0.15)
**Bahamas**	2.20(1.86 to 2.54)	0.84(0.71 to 0.97)	0.02(–0.01 to 0.06)
**Bahrain**	1.44(0.83 to 2.05)	0.50(0.30 to 0.69)	0.09(0.04 to 0.14)
**Bangladesh**	–1.14(–1.3 to –0.99)	–0.70(–0.79 to –0.60)	0.14(0.11 to 0.17)
**Barbados**	3.18(2.59 to 3.78)	0.89(0.75 to 1.03)	0.01(–0.03 to 0.05)
**Belarus**	–1.11(–1.60 to –0.61)	–1.01(–1.14 to –0.89)	–0.01(–0.03 to 0.01)
**Belgium**	3.77(3.27 to 4.27)	1.77(1.07 to 2.47)	–0.13(–0.21 to –0.04)
**Belize**	3.51(2.77 to 4.25)	1.75(1.33 to 2.18)	0.12(0.07 to 0.17)
**Benin**	–1.10(–1.17 to –1.03)	–0.67(–0.72 to –0.62)	0.07(0.05 to 0.08)
**Bermuda**	1.10(0.86 to 1.34)	0.33(0.20 to 0.47)	0.01(–0.02 to 0.05)
**Bhutan**	–0.15(–0.22 to –0.09)	–0.86(–0.95 to –0.77)	0.18(0.16 to 0.19)
**Bolivia**	0.25(0.18 to 0.33)	–0.36(–0.45 to –0.27)	0.02(–0.05 to 0.08)
**Bosnia and Herzegovina**	–5.46(–6.28 to –4.63)	–1.44(–1.74 to –1.14)	0.11(0.06 to 0.15)
**Botswana**	–0.01(–0.38 to 0.35)	0.00(–0.19 to 0.19)	0.08(0.06 to 0.11)
**Brazil**	2.26(1.96 to 2.55)	2.08(1.95 to 2.21)	–0.05(–0.11 to 0.01)
**Brunei**	0.57(0.26 to 0.87)	–0.56(–0.78 to –0.33)	–0.12(–0.14 to –0.10)
**Bulgaria**	–5.09(–5.97 to –4.21)	–5.78(–6.81 to –4.74)	0.03(–0.01 to 0.06)
**Burkina Faso**	–0.01(–0.20 to 0.19)	–0.22(–0.29 to –0.14)	0.06(0.04 to 0.09)
**Burundi**	–0.51(–0.65 to –0.38)	–1.25(–1.38 to –1.12)	–0.04(–0.05 to –0.03)
**Cambodia**	–0.71(–0.79 to –0.63)	–1.19(–1.29 to –1.08)	0.11(0.09 to 0.12)

Location	EAPC (95% CI)		
	Urinary tract infections	Urolithiasis	Benign prostatic hyperplasia
**Cameroon**	–1.03(–1.15 to –0.90)	–1.01(–1.10 to –0.92)	0.11(0.09 to 0.13)
**Canada**	1.21(0.93 to 1.50)	0.76(0.59 to 0.93)	0.07(0.04 to 0.10)
**Cape Verde**	–0.37(–0.59 to –0.16)	0.14(0.07 to 0.22)	0.06(0.04 to 0.08)
**Central African Republic**	–0.27(–0.35 to –0.18)	0.15(0.01 to 0.29)	0.03(0.01 to 0.04)
**Chad**	–1.12(–1.29 to –0.95)	–0.66(–0.72 to –0.60)	0.05(0.03 to 0.06)
**Chile**	0.14(–0.14 to 0.42)	–1.09(–1.34 to –0.83)	–0.01(–0.04 to 0.02)
**China**	–3.19(–3.60 to –2.77)	–4.57(–4.84 to –4.31)	–0.18(–0.32 to –0.03)
**Colombia**	1.77(1.23 to 2.32)	0.90(0.54 to 1.26)	0.01(–0.02 to 0.04)
**Comoros**	–0.17(–0.38 to 0.04)	–0.40(–0.56 to –0.25)	–0.02(–0.03 to –0.01)
**Congo**	–1.21(–1.41 to –1.00)	–1.22(–1.38 to –1.05)	0.02(0.01 to 0.02)
**Cook Islands**	–0.57(–0.76 to –0.37)	–0.73(–0.86 to –0.59)	0.26(0.22 to 0.29)
**Costa Rica**	3.27(2.91 to 3.64)	0.83(0.70 to 0.96)	0.01(–0.01 to 0.03)
**Croatia**	1.20(0.89 to 1.51)	0.57(0.39 to 0.74)	–0.13(–0.22 to –0.04)
**Cuba**	2.81(2.60 to 3.02)	1.30(1.13 to 1.48)	–0.03(–0.06 to 0.01)
**Cyprus**	–0.48(–0.85 to –0.10)	–1.89(–2.4 to –1.38)	–0.07(–0.09 to –0.06)
**Czech Republic**	–2.92(–3.59 to –2.25)	–3.43(–4.16 to –2.70)	0.02(–0.01 to 0.04)
**Democratic Republic of the Congo**	–0.36(–0.43 to –0.29)	–0.18(–0.27 to –0.08)	0.02(0.01 to 0.03)
**Denmark**	0.81(0.29 to 1.32)	0.09(–0.07 to 0.24)	–0.04(–0.08 to 0.00)
**Djibouti**	0.70(0.44 to 0.96)	0.02(–0.11 to 0.15)	0.02(0.01 to 0.02)
**Dominica**	2.65(2.38 to 2.92)	0.96(0.90 to 1.01)	0.01(–0.04 to 0.05)
**Dominican Republic**	0.93(0.77 to 1.08)	0.11(–0.06 to 0.29)	0.04(0.01 to 0.07)
**Ecuador**	1.99(1.65 to 2.32)	1.39(1.11 to 1.67)	0.38(0.09 to 0.68)
**Egypt**	–0.50(–0.57 to –0.43)	0.28(0.25 to 0.31)	0.13(0.12 to 0.15)
**El Salvador**	–1.01(–1.23 to –0.79)	–0.22(–0.34 to –0.11)	0.08(0.06 to 0.09)
**Equatorial Guinea**	–1.20(–1.55 to –0.85)	–1.71(–2.08 to –1.34)	0.17(0.13 to 0.20)
**Eritrea**	0.08(–0.09 to 0.25)	–0.02(–0.12 to 0.08)	–0.03(–0.04 to –0.02)
**Estonia**	–4.51(–4.88 to –4.13)	–2.95(–3.34 to –2.55)	0.07(0.06 to 0.08)
**Eswatini**	0.12(–0.49 to 0.74)	0.36(–0.13 to 0.85)	0.13(0.10 to 0.16)
**Ethiopia**	–3.00(–3.14 to –2.87)	–2.84(–2.96 to –2.71)	0.01(–0.01 to 0.02)
**Fiji**	1.43(1.18 to 1.67)	1.09(0.89 to 1.29)	0.16(0.10 to 0.22)
**Finland**	–4.35(–4.84 to –3.85)	–0.71(–1.29 to –0.13)	0.02(0.00 to 0.03)
**France**	–0.33(–0.55 to –0.12)	–0.43(–0.48 to –0.37)	0.00(–0.03 to 0.03)
**Gabon**	–0.14(–0.30 to 0.02)	–0.56(–0.71 to –0.42)	0.03(0.01 to 0.05)

Location	EAPC (95% CI)		
	Urinary tract infections	Urolithiasis	Benign prostatic hyperplasia
**Gambia**	–0.19(–0.37 to –0.02)	–0.17(–0.25 to –0.08)	0.02(0.01 to 0.04)
**Georgia**	2.25(1.58 to 2.92)	–0.19(–0.33 to –0.06)	–0.01(–0.11 to 0.09)
**Germany**	2.01(1.76 to 2.26)	1.22(0.77 to 1.68)	–0.01(–0.04 to 0.03)
**Ghana**	0.23(–0.01 to 0.47)	0.32(0.17 to 0.48)	0.09(0.08 to 0.11)
**Greece**	–0.45(–1.51 to 0.61)	0.09(0.03 to 0.14)	–0.03(–0.06 to –0.01)
**Greenland**	–1.02(–1.31 to –0.72)	–0.30(–0.41 to –0.20)	0.03(0.01 to 0.06)
**Grenada**	3.68(3.28 to 4.09)	2.19(1.97 to 2.40)	0.12(0.07 to 0.16)
**Guam**	–1.66(–2.14 to –1.17)	–3.21(–3.9 to –2.52)	0.23(0.20 to 0.25)
**Guatemala**	2.70(2.28 to 3.12)	–1.07(–1.36 to –0.79)	0.16(0.14 to 0.18)
**Guinea**	–0.94(–1.02 to –0.85)	–0.37(–0.44 to –0.31)	0.03(0.01 to 0.04)
**Guinea-Bissau**	–1.79(–1.89 to –1.69)	–1.17(–1.22 to –1.13)	0.05(0.03 to 0.07)
**Guyana**	3.10(2.64 to 3.57)	1.41(1.09 to 1.74)	0.10(0.03 to 0.16)
**Haiti**	0.80(0.67 to 0.93)	0.28(0.15 to 0.41)	0.00(–0.03 to 0.04)
**Honduras**	0.30(0.11 to 0.49)	–0.68(–0.78 to –0.59)	0.06(0.05 to 0.07)
**Hungary**	–2.61(–3.00 to –2.22)	–3.55(–4.16 to –2.95)	0.07(0.03 to 0.11)
**Iceland**	0.67(0.35 to 0.99)	–0.88(–1.08 to –0.67)	–0.02(–0.06 to 0.01)
**India**	–0.70(–0.78 to –0.62)	–1.37(–1.56 to –1.17)	0.35(0.17 to 0.53)
**Indonesia**	–0.15(–0.20 to –0.09)	–1.27(–1.40 to –1.14)	–0.43(–0.74 to –0.12)
**Iran**	0.87(0.45 to 1.29)	0.44(0.27 to 0.61)	0.10(0.08 to 0.12)
**Iraq**	–3.01(–3.40 to –2.63)	–1.73(–1.94 to –1.52)	0.12(0.11 to 0.14)
**Ireland**	–0.90(–1.06 to –0.74)	–0.49(–0.67 to –0.32)	–0.03(–0.06 to –0.01)
**Israel**	2.11(1.13 to 3.11)	0.40(0.09 to 0.71)	0.00(–0.03 to 0.03)
**Italy**	0.04(–0.38 to 0.46)	–1.41(–1.65 to –1.16)	–0.09(–0.13 to –0.05)
**Ivory Coast**	–1.30(–1.44 to –1.15)	–0.52(–0.57 to –0.46)	0.06(0.04 to 0.07)
**Jamaica**	2.70(2.01 to 3.40)	2.27(2.05 to 2.49)	0.12(0.08 to 0.16)
**Japan**	0.82(0.58 to 1.06)	0.39(0.31 to 0.48)	–0.08(–0.11 to –0.04)
**Jordan**	0.97(0.76 to 1.19)	1.54(1.26 to 1.82)	0.20(0.08 to 0.32)
**Kazakhstan**	–0.51(–0.88 to –0.14)	–0.34(–0.64 to –0.04)	0.07(0.03 to 0.11)
**Kenya**	0.95(0.77 to 1.14)	0.79(0.68 to 0.89)	0.00(–0.01 to 0.01)
**Kiribati**	–0.76(–0.81 to –0.70)	–1.08(–1.15 to –1.02)	0.25(0.19 to 0.30)
**Kuwait**	4.05(3.44 to 4.66)	0.22(0.18 to 0.25)	0.03(–0.01 to 0.07)
**Kyrgyzstan**	1.10(0.10 to 2.12)	–1.37(–1.80 to –0.94)	0.08(0.02 to 0.13)
**Laos**	–1.54(–1.71 to –1.37)	–2.20(–2.38 to –2.01)	0.14(0.12 to 0.15)

Location	EAPC (95% CI)		
	Urinary tract infections	Urolithiasis	Benign prostatic hyperplasia
**Latvia**	–2.21(–2.67 to –1.76)	–2.13(–2.45 to –1.81)	0.10(0.09 to 0.11)
**Lebanon**	–0.11(–0.28 to 0.05)	0.27(0.23 to 0.30)	–0.06(–0.07 to –0.04)
**Lesotho**	2.26(1.92 to 2.60)	1.64(1.37 to 1.90)	0.15(0.12 to 0.17)
**Liberia**	–1.93(–2.23 to –1.63)	–0.88(–1.05 to –0.72)	0.03(0.01 to 0.04)
**Libya**	1.12(0.88 to 1.36)	0.35(0.28 to 0.41)	0.01(–0.01 to 0.04)
**Lithuania**	–0.66(–0.99 to –0.33)	–2.87(–3.18 to –2.56)	0.05(0.05 to 0.06)
**Luxembourg**	0.80(0.64 to 0.95)	–0.32(–0.84 to 0.20)	–0.03(–0.06 to 0.00)
**Macedonia**	–1.99(–2.45 to –1.53)	–0.12(–0.19 to –0.06)	0.08(0.04 to 0.12)
**Madagascar**	–0.39(–0.45 to –0.33)	–0.41(–0.48 to –0.34)	0.02(0.01 to 0.03)
**Malawi**	–0.30(–0.41 to –0.2)	–0.73(–0.80 to –0.65)	0.01(0.00 to 0.02)
**Malaysia**	1.18(0.92 to 1.45)	0.25(0.16 to 0.34)	0.13(0.11 to 0.15)
**Maldives**	–2.48(–2.69 to –2.26)	–0.49(–0.68 to –0.30)	0.06(0.05 to 0.08)
**Mali**	–1.29(–1.48 to –1.09)	–1.09(–1.21 to –0.96)	0.06(0.04 to 0.08)
**Malta**	0.82(0.61 to 1.04)	0.72(0.32 to 1.12)	0.00(–0.02 to 0.01)
**Marshall Islands**	–0.26(–0.38 to –0.13)	–0.62(–0.71 to –0.52)	0.21(0.19 to 0.24)
**Mauritania**	–2.02(–2.07 to –1.96)	–1.59(–1.69 to –1.49)	0.04(0.02 to 0.05)
**Mauritius**	4.16(3.55 to 4.78)	–0.19(–0.35 to –0.03)	0.46(0.42 to 0.49)
**Mexico**	1.88(1.24 to 2.52)	0.00(–0.36 to 0.37)	–0.02(–0.06 to 0.02)
**Micronesia**	–0.55(–0.71 to –0.40)	–1.09(–1.19 to –1.00)	0.31(0.27 to 0.35)
**Moldova**	–1.27(–1.55 to –0.99)	0.01(–0.33 to 0.35)	0.02(0.01 to 0.03)
**Monaco**	–0.43(–0.56 to –0.30)	–0.02(–0.06 to 0.01)	–0.04(–0.07 to –0.01)
**Mongolia**	–4.45(–4.98 to –3.91)	–2.01(–2.33 to –1.68)	0.09(0.05 to 0.14)
**Montenegro**	–0.32(–0.43 to –0.21)	0.01(0.00 to 0.03)	0.05(0.01 to 0.10)
**Morocco**	1.03(0.89 to 1.17)	0.43(0.38 to 0.47)	0.07(0.06 to 0.08)
**Mozambique**	0.84(0.71 to 0.96)	0.05(–0.11 to 0.20)	0.00(–0.01 to 0.01)
**Myanmar**	–1.35(–1.60 to –1.11)	–1.79(–2.05 to –1.53)	0.16(0.14 to 0.17)
**Namibia**	–0.90(–1.32 to –0.47)	–0.58(–0.81 to –0.35)	–0.02(–0.05 to 0.01)
**Nauru**	–0.73(–1.08 to –0.38)	–0.98(–1.13 to –0.84)	0.22(0.20 to 0.24)
**Nepal**	0.31(0.00 to 0.63)	–0.51(–0.76 to –0.26)	0.10(0.04 to 0.17)
**Netherlands**	–0.22(–0.78 to 0.33)	–1.51(–1.69 to –1.33)	0.00(–0.04 to 0.03)
**New Zealand**	–0.39(–0.93 to 0.14)	–0.90(–1.28 to –0.53)	–0.32(–0.41 to –0.23)
**Nicaragua**	0.46(0.10 to 0.82)	0.51(0.22 to 0.80)	0.06(0.04 to 0.08)
**Niger**	–1.93(–2.09 to –1.76)	–1.20(–1.31 to –1.09)	0.05(0.04 to 0.07)

Location	EAPC (95% CI)		
	Urinary tract infections	Urolithiasis	Benign prostatic hyperplasia
**Nigeria**	–1.20(–1.32 to –1.08)	–0.69(–0.77 to –0.62)	0.04(0.03 to 0.05)
**Niue**	–1.38(–1.51 to –1.24)	–1.00(–1.08 to –0.93)	0.23(0.20 to 0.26)
**North Korea**	–1.32(–1.42 to –1.22)	–0.82(–0.98 to –0.67)	0.12(0.1 to 0.14)
**Northern Mariana Islands**	–1.61(–1.87 to –1.34)	–1.14(–1.46 to –0.83)	0.20(0.17 to 0.23)
**Norway**	0.31(0.08 to 0.55)	–0.21(–0.43 to 0.00)	0.06(0.04 to 0.08)
**Oman**	2.59(1.81 to 3.37)	0.64(0.54 to 0.74)	0.16(0.14 to 0.17)
**Pakistan**	0.86(0.60 to 1.12)	–0.23(–0.41 to –0.06)	0.31(0.30 to 0.33)
**Palau**	–0.73(–0.77 to –0.70)	–0.62(–0.67 to –0.58)	0.25(0.22 to 0.29)
**Palestine**	–1.32(–1.76 to –0.87)	–0.19(–0.30 to –0.07)	0.10(0.09 to 0.11)
**Panama**	3.40(3.02 to 3.77)	0.25(0.07 to 0.44)	0.07(0.05 to 0.08)
**Papua New Guinea**	–0.33(–0.39 to –0.27)	–0.65(–0.70 to –0.60)	0.24(0.21 to 0.26)
**Paraguay**	2.95(2.51 to 3.38)	1.25(0.95 to 1.55)	0.14(0.13 to 0.15)
**Peru**	0.40(–0.23 to 1.03)	–0.07(–0.17 to 0.03)	0.02(–0.04 to 0.09)
**Philippines**	–0.15(–0.31 to 0.00)	–0.66(–0.76 to –0.57)	0.08(0.07 to 0.09)
**Poland**	–0.49(–1.56 to 0.60)	–5.84(–6.31 to –5.37)	–0.69(–1.03 to –0.35)
**Portugal**	6.44(5.67 to 7.21)	0.38(0.09 to 0.66)	0.02(–0.01 to 0.05)
**Puerto Rico**	2.12(1.37 to 2.89)	0.65(0.54 to 0.76)	0.08(0.04 to 0.12)
**Qatar**	–0.01(–0.27 to 0.25)	0.27(0.21 to 0.34)	0.04(0.00 to 0.08)
**Romania**	–2.37(–2.74 to –2.00)	1.86(1.27 to 2.46)	0.06(0.04 to 0.08)
**Russia**	–0.92(–1.23 to –0.60)	–1.06(–1.38 to –0.75)	0.04(0.03 to 0.05)
**Rwanda**	–1.21(–1.41 to –1.01)	–1.87(–2.13 to –1.61)	–0.02(–0.03 to –0.01)
**Saint Kitts and Nevis**	2.26(1.90 to 2.61)	0.24(0.12 to 0.37)	0.05(0.01 to 0.09)
**Saint Lucia**	2.66(2.35 to 2.97)	0.84(0.70 to 0.98)	–0.05(–0.09 to –0.01)
**Saint Vincent and the Grenadines**	2.61(2.39 to 2.82)	0.93(0.73 to 1.12)	0.08(0.03 to 0.13)
**Samoa**	–1.29(–1.38 to –1.21)	–1.20(–1.29 to –1.11)	0.23(0.19 to 0.27)
**San Marino**	1.89(1.60 to 2.17)	0.46(0.30 to 0.61)	–0.02(–0.05 to 0.01)
**Sao Tome and Principe**	0.01(–0.25 to 0.28)	–0.43(–0.56 to –0.29)	0.01(0.00 to 0.03)
**Saudi Arabia**	–0.11(–0.51 to 0.29)	0.23(0.21 to 0.26)	0.00(–0.02 to 0.02)
**Senegal**	–1.28(–1.44 to –1.11)	–0.91(–1.01 to –0.82)	0.05(0.04 to 0.07)
**Serbia**	–2.86(–3.17 to –2.55)	0.87(0.51 to 1.23)	0.05(0.03 to 0.07)
**Seychelles**	2.72(2.43 to 3.01)	1.04(0.80 to 1.27)	0.09(0.08 to 0.10)
**Sierra Leone**	–0.63(–0.71 to –0.54)	–0.26(–0.30 to –0.22)	0.05(0.04 to 0.07)
**Singapore**	–1.98(–2.49 to –1.48)	–1.09(–1.43 to –0.75)	–0.09(–0.11 to –0.07)

Location	EAPC (95% CI)		
	Urinary tract infections	Urolithiasis	Benign prostatic hyperplasia
**Slovakia**	–0.87(–1.23 to –0.50)	–1.43(–1.83 to –1.02)	–0.01(–0.01 to 0.00)
**Slovenia**	–3.90(–4.31 to –3.49)	–0.91(–1.23 to –0.60)	0.01(–0.01 to 0.04)
**Solomon Islands**	–0.18(–0.30 to –0.05)	–0.56(–0.66 to –0.46)	0.30(0.27 to 0.32)
**Somalia**	0.40(0.32 to 0.49)	0.21(0.10 to 0.32)	–0.02(–0.03 to –0.01)
**South Africa**	–0.98(–1.76 to –0.19)	–0.49(–0.88 to –0.11)	0.11(0.08 to 0.14)
**South Korea**	–0.30(–0.82 to 0.23)	–0.40(–0.54 to –0.25)	0.05(0.01 to 0.09)
**South Sudan**	–0.26(–0.31 to –0.20)	–0.35(–0.45 to –0.25)	0.02(0.01 to 0.02)
**Spain**	1.99(1.64 to 2.33)	–0.37(–0.47 to –0.27)	0.00(–0.04 to 0.03)
**Sri Lanka**	3.46(2.51 to 4.42)	0.17(0.11 to 0.23)	0.19(0.18 to 0.21)
**Sudan**	0.28(0.06 to 0.50)	0.43(0.37 to 0.49)	0.06(0.04 to 0.08)
**Suriname**	2.68(2.25 to 3.12)	1.65(1.34 to 1.96)	0.13(0.09 to 0.16)
**Sweden**	–1.57(–1.86 to –1.29)	–0.47(–0.89 to –0.06)	–0.18(–0.28 to –0.08)
**Switzerland**	0.91(0.58 to 1.25)	–0.34(–0.59 to –0.08)	0.02(0.01 to 0.03)
**Syria**	–3.88(–4.54 to –3.22)	–0.47(–0.56 to –0.39)	0.00(–0.02 to 0.02)
**Tajikistan**	1.63(1.15 to 2.11)	0.74(0.56 to 0.91)	0.08(0.04 to 0.12)
**Tanzania**	0.49(0.34 to 0.64)	–0.10(–0.22 to 0.02)	0.03(0.02 to 0.04)
**Thailand**	–3.67(–4.22 to –3.12)	–2.36(–2.89 to –1.83)	–0.01(–0.02 to 0.00)
**Timor-Leste**	–0.49(–0.74 to –0.24)	–0.59(–0.76 to –0.41)	0.12(0.08 to 0.16)
**Togo**	–1.33(–1.47 to –1.20)	–0.78(–0.86 to –0.69)	0.04(0.02 to 0.06)
**Tokelau**	–1.48(–1.57 to –1.40)	–1.39(–1.48 to –1.31)	0.26(0.23 to 0.29)
**Tonga**	–0.16(–0.42 to 0.10)	–0.41(–0.51 to –0.30)	0.22(0.19 to 0.26)
**Trinidad and Tobago**	1.64(1.26 to 2.02)	2.84(2.30 to 3.38)	0.01(–0.03 to 0.05)
**Tunisia**	0.16(0.03 to 0.29)	0.24(0.21 to 0.26)	–0.01(–0.03 to 0.01)
**Turkey**	1.12(0.58 to 1.67)	0.07(–0.09 to 0.22)	–0.05(–0.06 to –0.03)
**Turkmenistan**	5.21(4.33 to 6.10)	1.79(1.65 to 1.93)	0.12(0.08 to 0.15)
**Tuvalu**	–1.64(–1.7 to –1.57)	–1.45(–1.53 to –1.36)	0.25(0.22 to 0.28)
**Uganda**	0.18(–0.05 to 0.42)	–0.10(–0.30 to 0.10)	0.00(–0.01 to 0.00)
**UK**	3.05(2.30 to 3.82)	0.65(0.41 to 0.90)	–0.1(–0.13 to –0.07)
**Ukraine**	–0.55(–0.93 to –0.16)	–1.07(–1.31 to –0.82)	0.03(0.02 to 0.03)
**United Arab Emirates**	0.05(–0.31 to 0.41)	0.55(0.46 to 0.65)	0.03(0.01 to 0.06)
**Uruguay**	4.80(4.16 to 5.44)	0.54(0.47 to 0.61)	0.00(–0.02 to 0.02)
**USA**	–0.03(–0.23 to 0.18)	–1.19(–1.48 to –0.9)	0.12(0.06 to 0.18)
**Uzbekistan**	1.81(1.26 to 2.35)	0.29(0.25 to 0.33)	0.11(0.07 to 0.14)

Location	EAPC (95% CI)		
	Urinary tract infections	Urolithiasis	Benign prostatic hyperplasia
**Vanuatu**	–0.11(–0.23 to 0.01)	–0.43(–0.55 to –0.31)	0.33(0.29 to 0.36)
**Venezuela**	2.14(1.53 to 2.75)	0.39(–0.19 to 0.97)	0.04(0.01 to 0.06)
**Vietnam**	–0.62(–0.98 to –0.27)	0.51(0.17 to 0.84)	0.29(0.19 to 0.38)
**Virgin Islands**	1.45(1.21 to 1.70)	1.39(1.17 to 1.62)	0.02(–0.01 to 0.06)
**Yemen**	0.71(0.51 to 0.92)	0.47(0.41 to 0.52)	0.06(0.05 to 0.08)
**Zambia**	–0.39(–0.6 to –0.18)	–0.86(–0.98 to –0.75)	0.03(0.02 to 0.04)
**Zimbabwe**	1.41(0.97 to 1.84)	0.52(0.20 to 0.85)	0.08(0.06 to 0.10)

EAPC estimated annual percentage change; *ASDR* age-standardized DALYs rate; *DALYs* disability-adjusted life-years; *CI* confidence interval
