# Supplementary material for: Epidemiological trends of urinary tract infections, urolithiasis and benign prostatic hyperplasia in 203 countries and territories from 1990 to 2019
Source: Mil Med Res. 2021 Dec 9;8:64. doi: 10.1186/s40779-021-00359-8 (PMC8656041; doi:10.1186/s40779-021-00359-8)
Supplement: Supplementary file 2 — Additional file 2: Fig. S1. Age-standardized DALYs rates for urolithiasis for 21 regions by SDI from 1990 to 2019. SDI sociodemographic index; DALYs disability-adjusted life-years. Fig. S2 Age-standardized mortality rates for urolithiasis for 21 regions by SDI from 1990 to 2019. SDI sociodemographic index. Fig. S3 Age-standardized incidence rates for urinary tract infections for 203 countries and territories by SDI in 2019. SDI sociodemographic index. Fig. S4 Age-standardized incidence rates for benign prostatic hyperplasia for 203 countries and territories by SDI in 2019.SDI sociodemographic index. Fig. S5 Age-standardized DALYs rates for urolithiasis for 203 countries and territories by SDI in 2019. SDI sociodemographic index; DALYs disability-adjusted life-years. Fig. S6 Age-standardized DALYs rates for benign prostatic hyperplasia for 203 countries and territories by SDI in 2019. SDI sociodemographic index; DALYs disability-adjusted life-years. Fig. S7 Age-standardized DALYs rates for urinary tract infections for 203 countries and territories by SDI in 2019. SDI sociodemographic index; DALYs disability-adjusted life-years. Fig. S8 Age-standardized mortality rates for urinary tract infections for 203 countries and territories by SDI in 2019. SDI sociodemographic index. Fig. S9 Age-standardized DALYs rates for urinary tract infections for 21 regions by SDI from 1990 to 2019. SDI sociodemographic index; DALYs disability-adjusted life-years. Fig. S10 Age-standardized mortality rates for urinary tract infections for 21 regions by SDI from 1990 to 2019. SDI sociodemographic index. Fig. S11 Age-standardized mortality rates for urolithiasis for 203 countries and territories by SDI in 2019. SDI sociodemographic index. Fig. S12 Age-standardized incidence rates for urinary tract infections for 21 regions by SDI from 1990 to 2019. SDI sociodemographic index. Fig. S13 Age-standardized incidence rates for benign prostatic hyperplasia for 21 regions by SDI from 1990 to 2019. S [file 40779_2021_359_MOESM2_ESM.pdf]

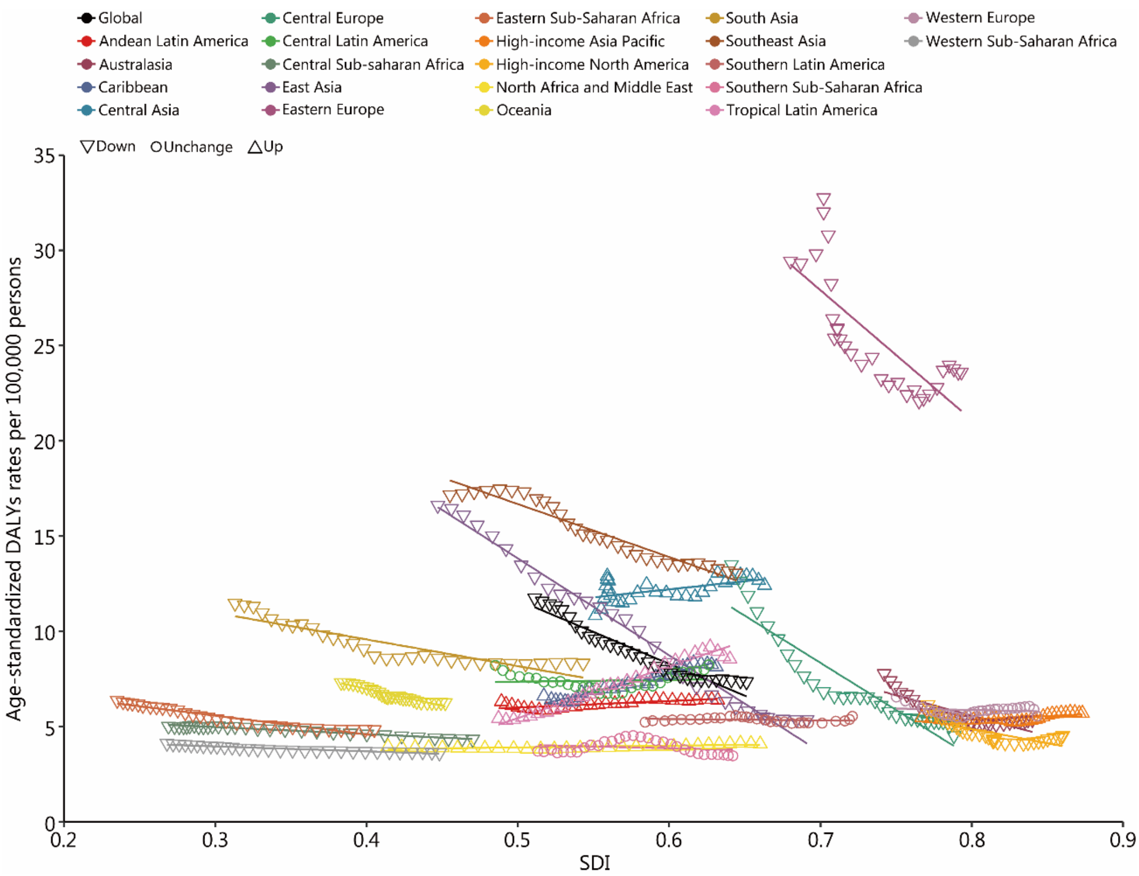

Fig. S1 Age-standardized DALYs rates for urolithiasis for 21 regions by SDI from 1990 to 2019. SDI sociodemographic index; DALYs disability-adjusted life-years

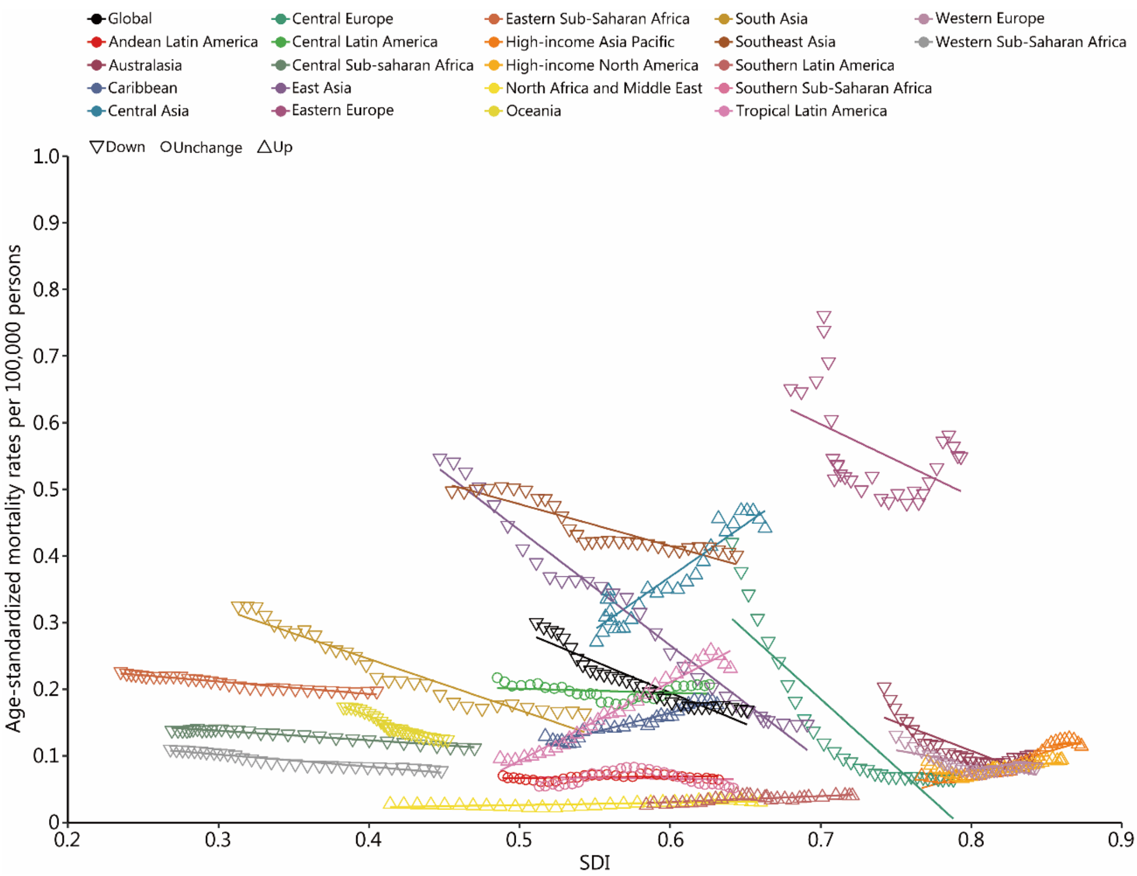

Fig. S2 Age-standardized mortality rates for urolithiasis for 21 regions by SDI from 1990 to 2019. SDI sociodemographic index

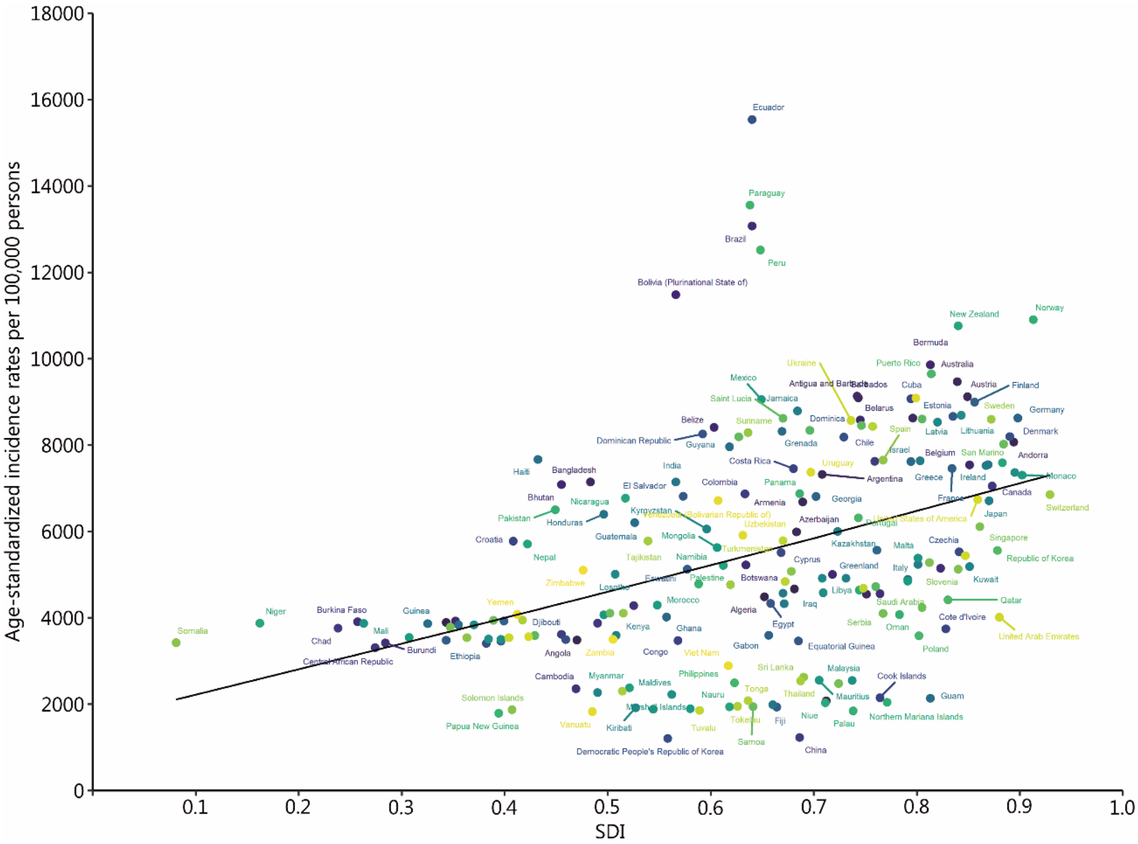

Fig. S3 Age-standardized incidence rates for urinary tract infections for 203 countries and territories by SDI in 2019. SDI sociodemographic index

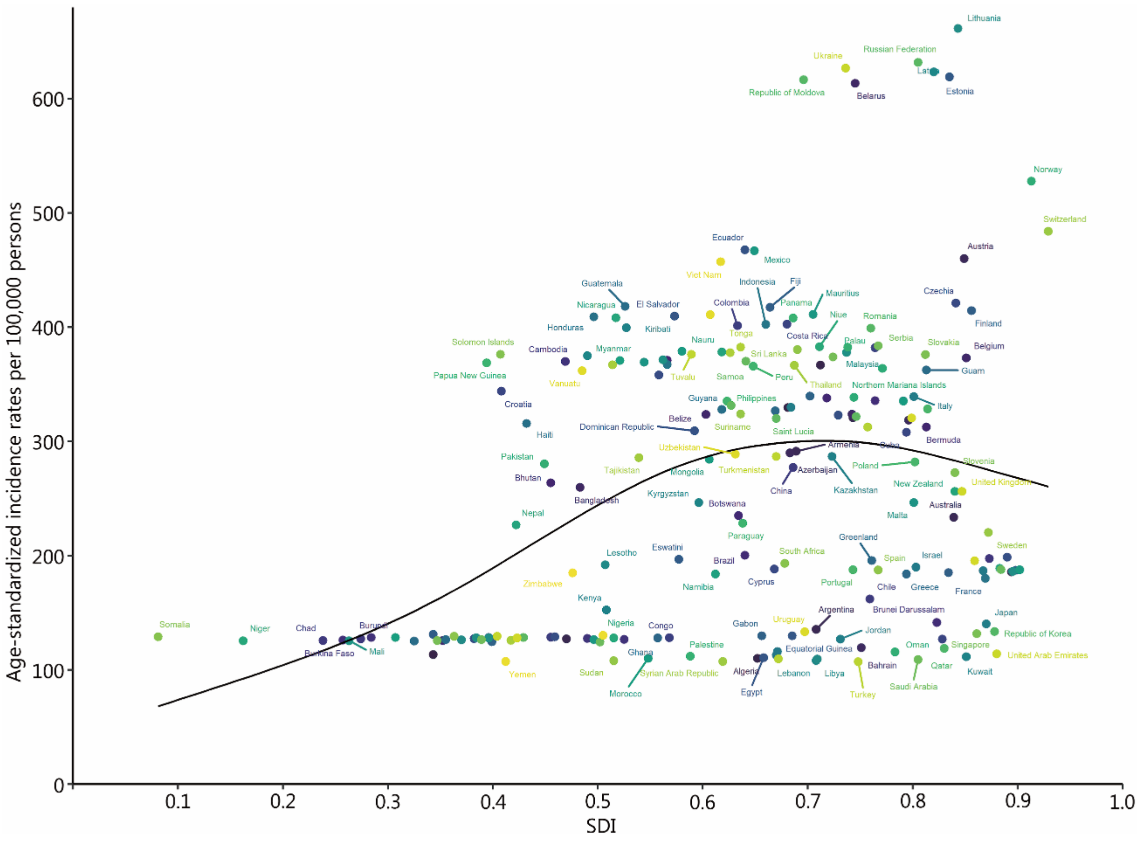

Fig. S4 Age-standardized incidence rates for benign prostatic hyperplasia for 203 countries and territories by SDI in 2019. SDI sociodemographic index

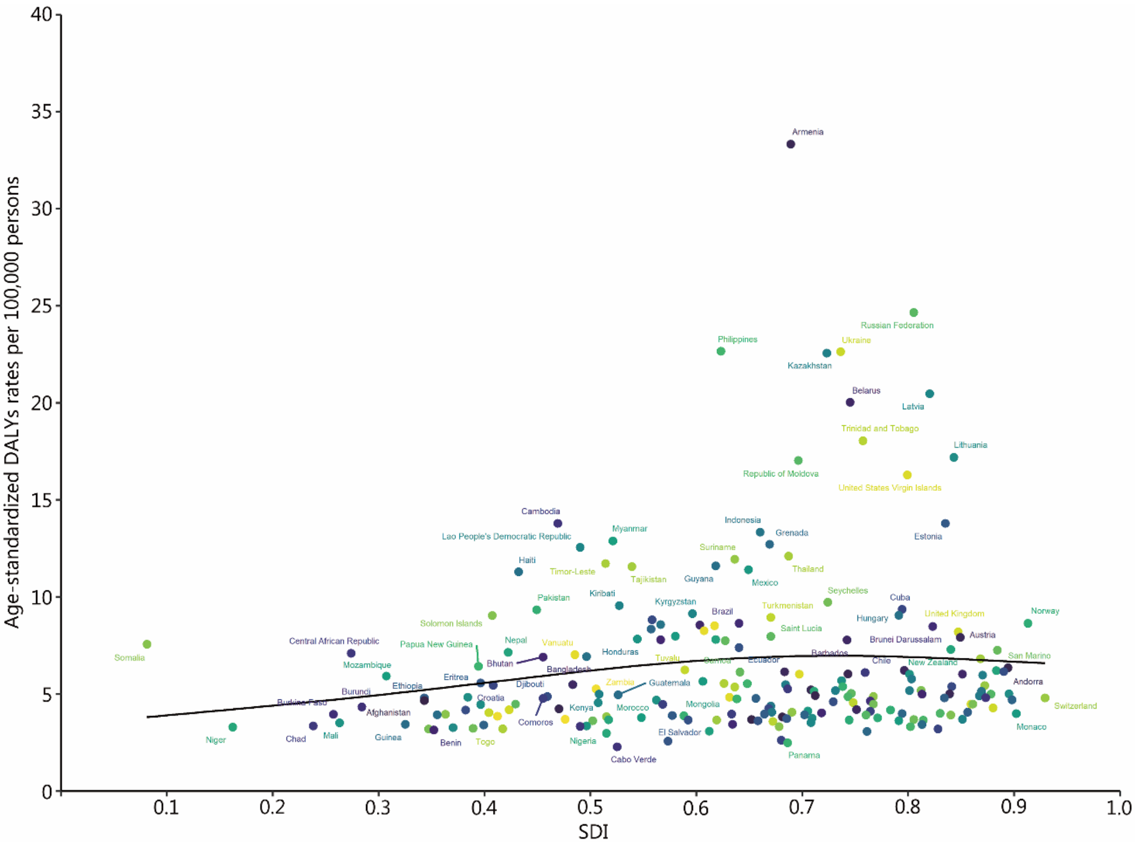

Fig. S5 Age-standardized DALYs rates for urolithiasis for 203 countries and territories by SDI in 2019. SDI sociodemographic index; DALYs disability-adjusted life-years

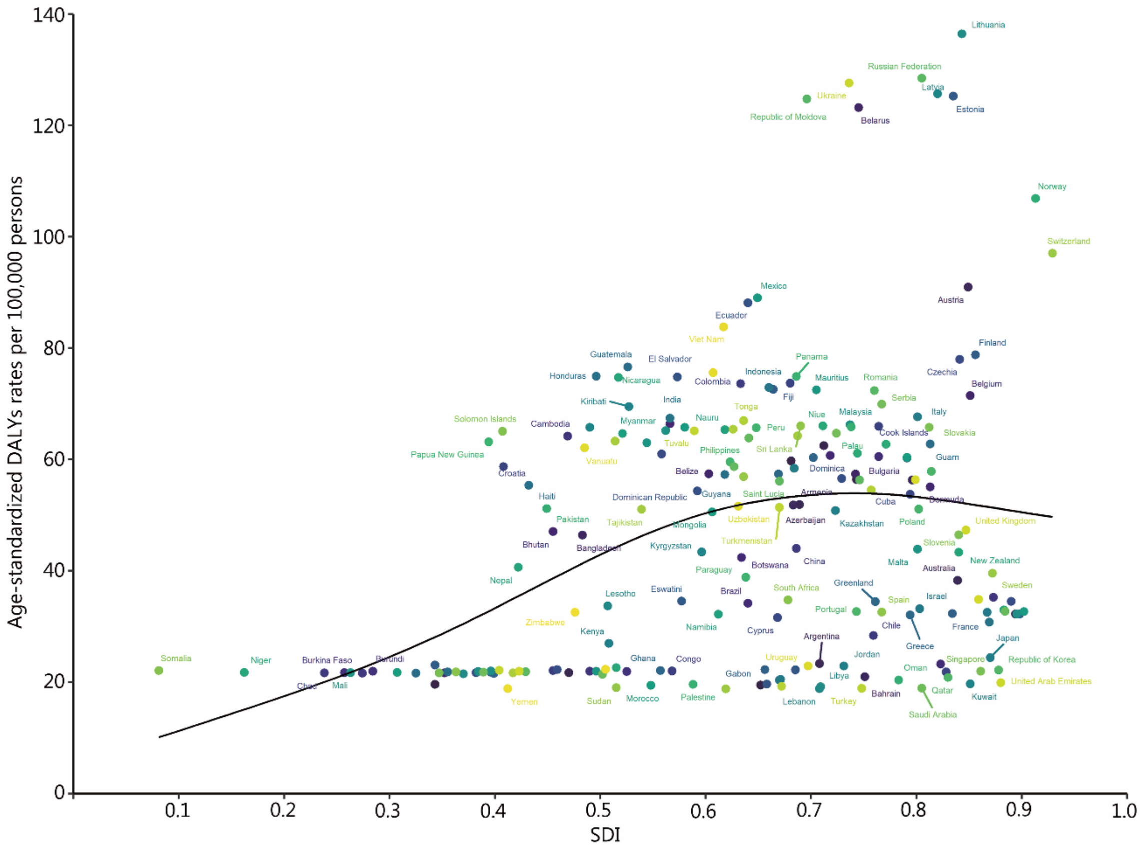

Fig. S6 Age-standardized DALYs rates for benign prostatic hyperplasia for 203 countries and territories by SDI in 2019. SDI sociodemographic index; DALYs disability-adjusted life-years

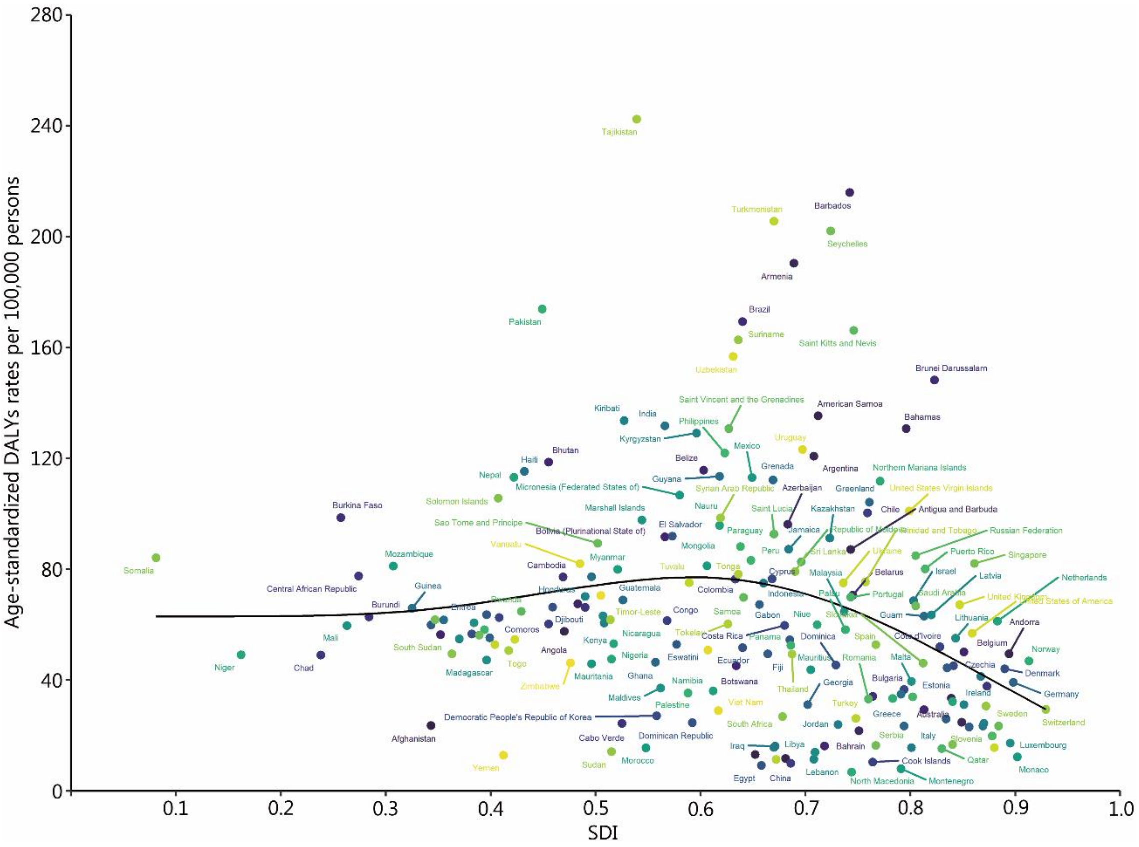

Fig. S7 Age-standardized DALYs rates for urinary tract infections for 203 countries and territories by SDI in 2019. SDI sociodemographic index; DALYs disability-adjusted life-years

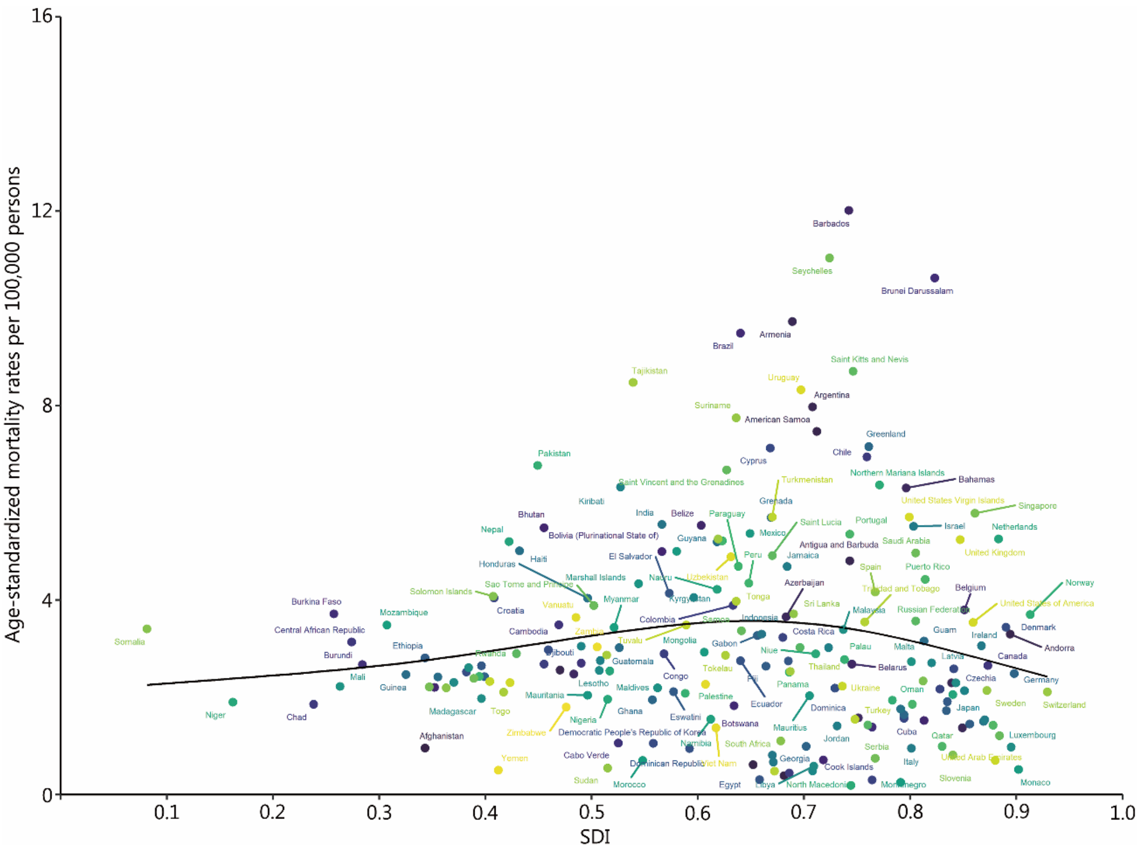

Fig. S8 Age-standardized mortality rates for urinary tract infections for 203 countries and territories by SDI in 2019. SDI sociodemographic index

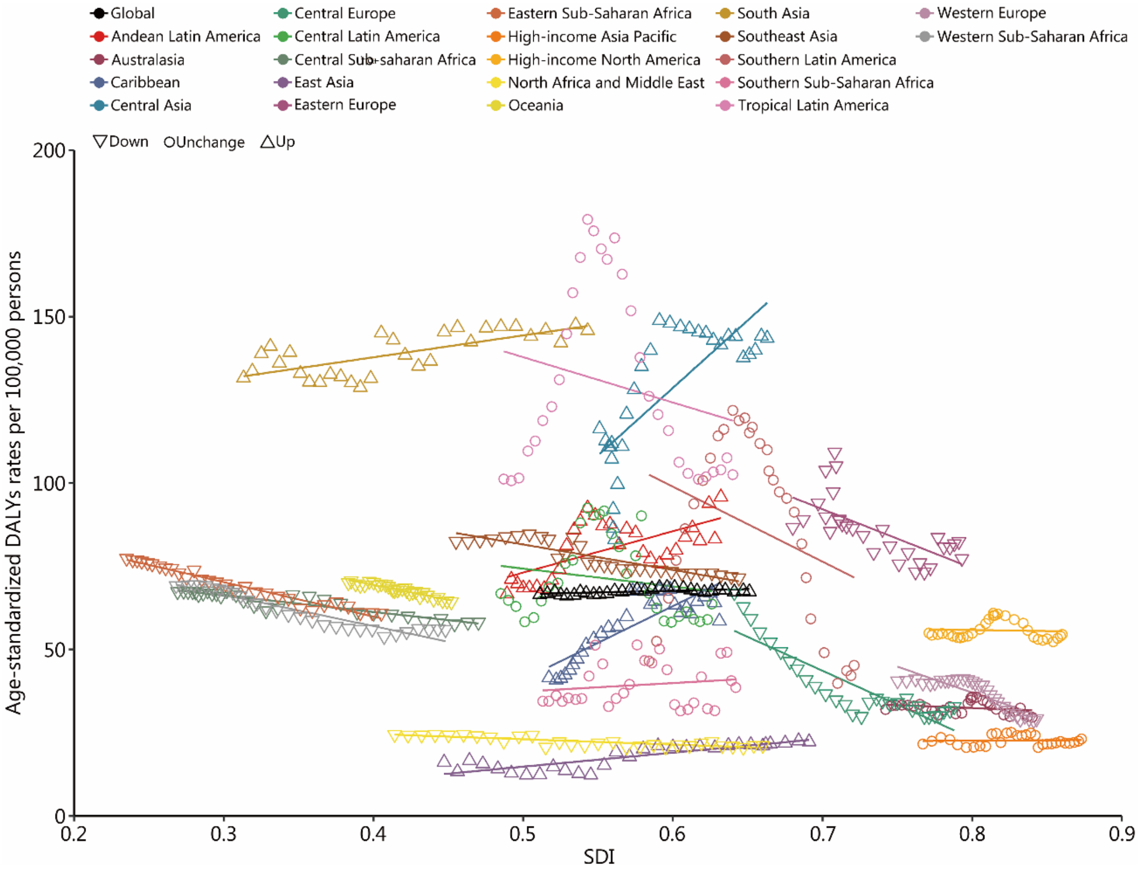

Fig. S9 Age-standardized DALYs rates for urinary tract infections for 21 regions by SDI from 1990 to 2019. *SDI* sociodemographic index; *DALYs* disability-adjusted life-years

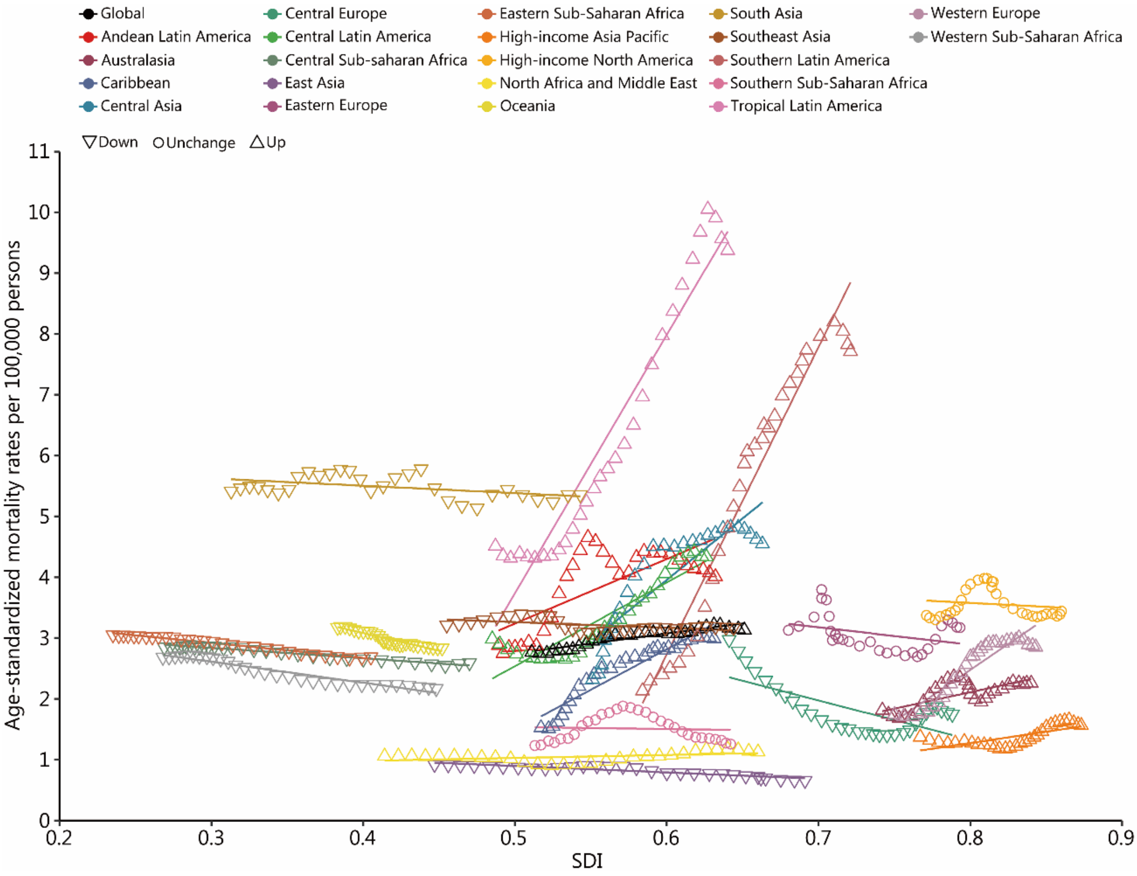

Fig. S10 Age-standardized mortality rates for urinary tract infections for 21 regions by SDI from 1990 to 2019. SDI sociodemographic index

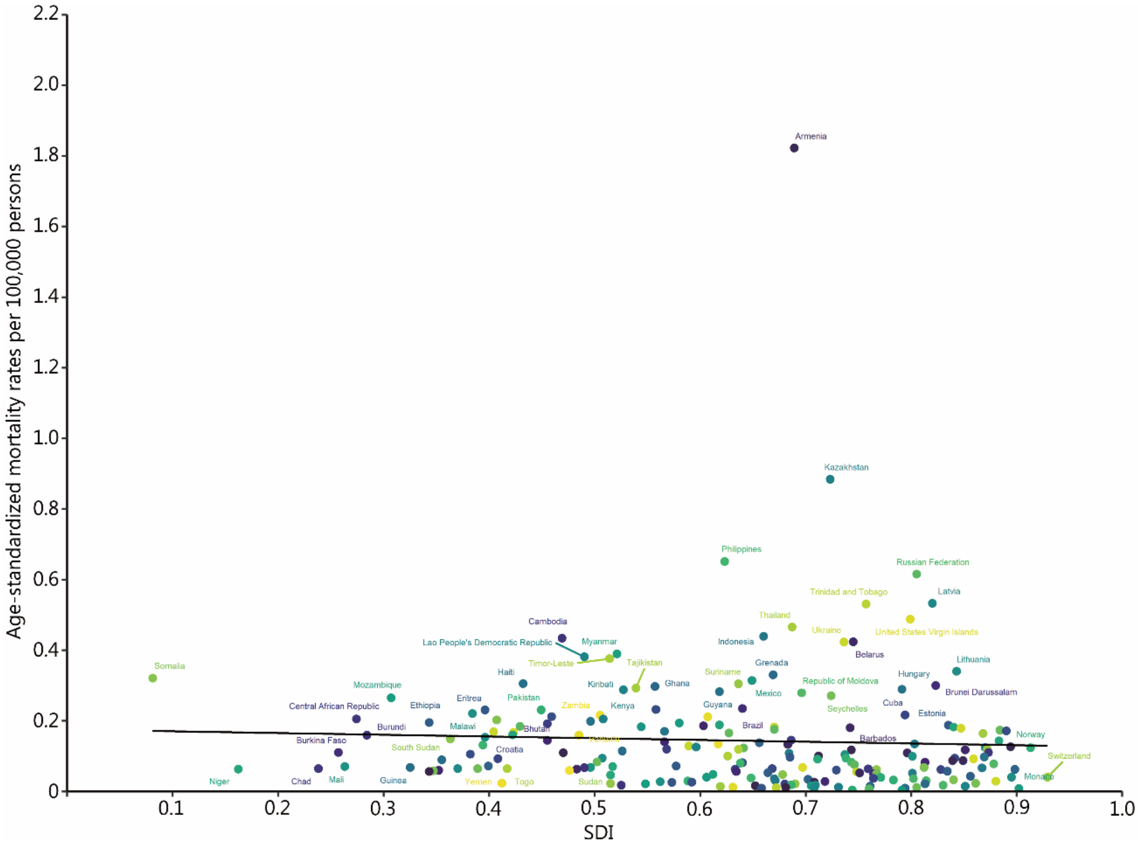

Fig. S11 Age-standardized mortality rates for urolithiasis for 203 countries and territories by SDI in 2019. SDI sociodemographic index

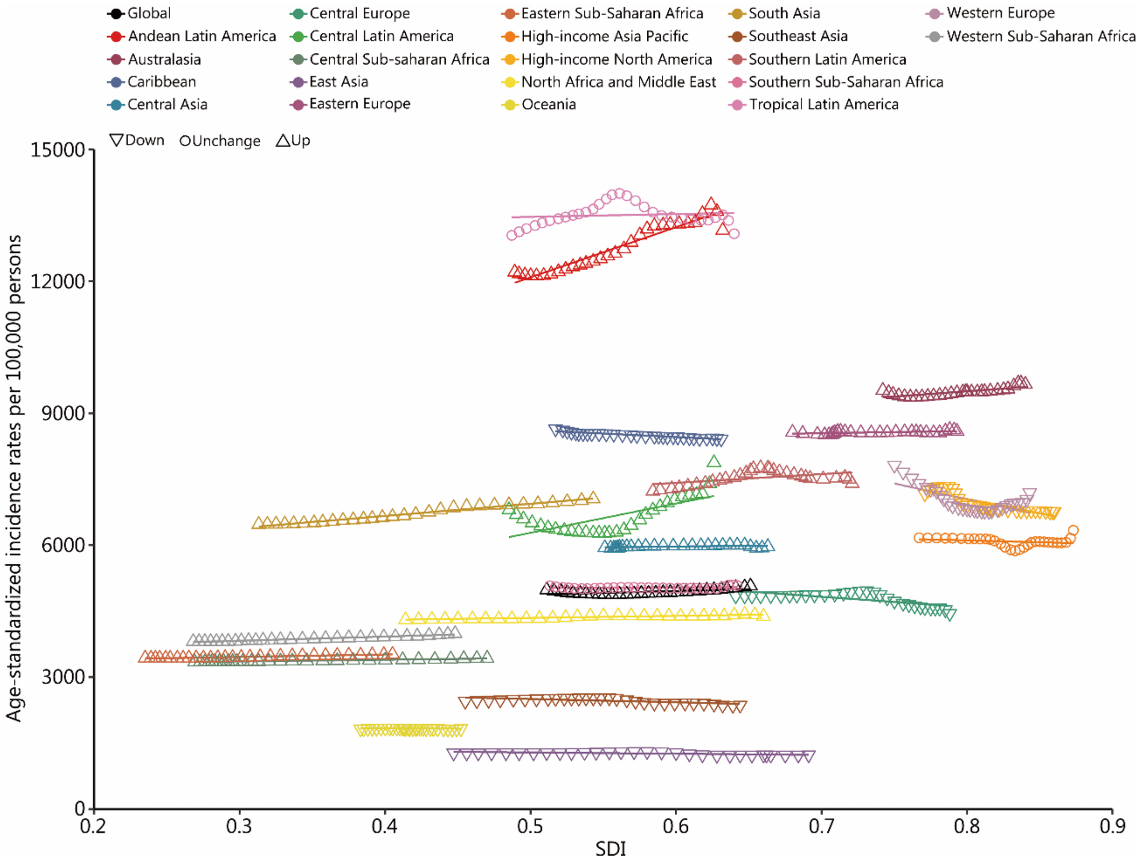

Fig. S12 Age-standardized incidence rates for urinary tract infections for 21 regions by SDI from 1990 to 2019. SDI sociodemographic index

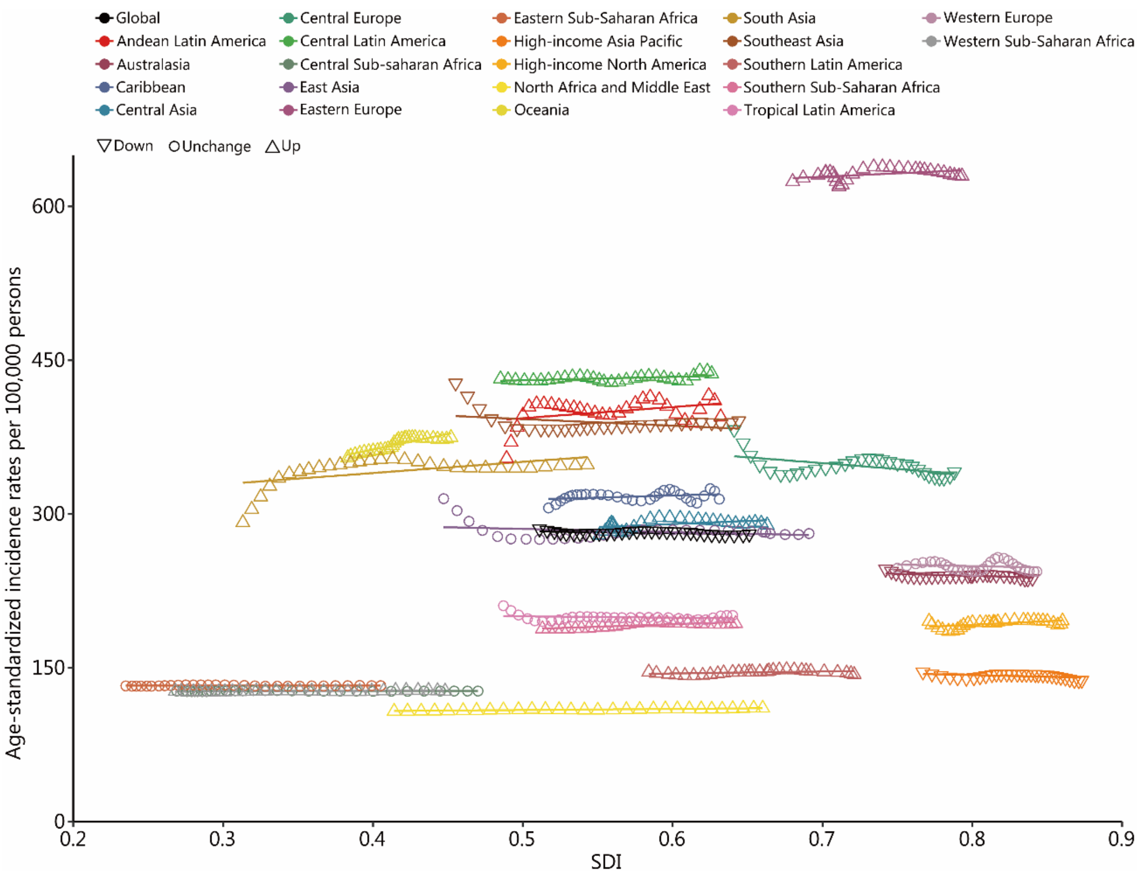

Fig. S13 Age-standardized incidence rates for benign prostatic hyperplasia for 21 regions by SDI from 1990 to 2019. SDI sociodemographic index

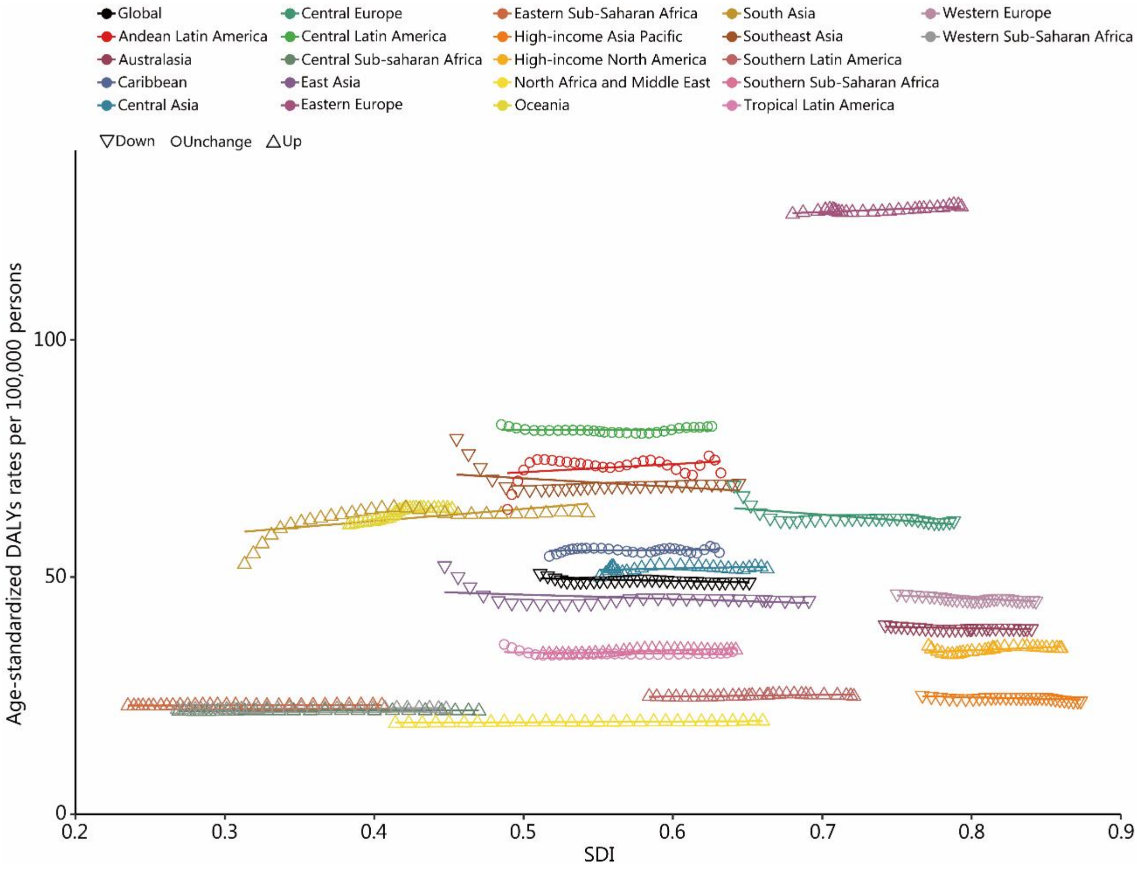

Fig. S14 Age-standardized DALYs rates for benign prostatic hyperplasia for 21 regions by SDI from 1990 to 2019. SDI sociodemographic index; DALYs disability-adjusted life-years

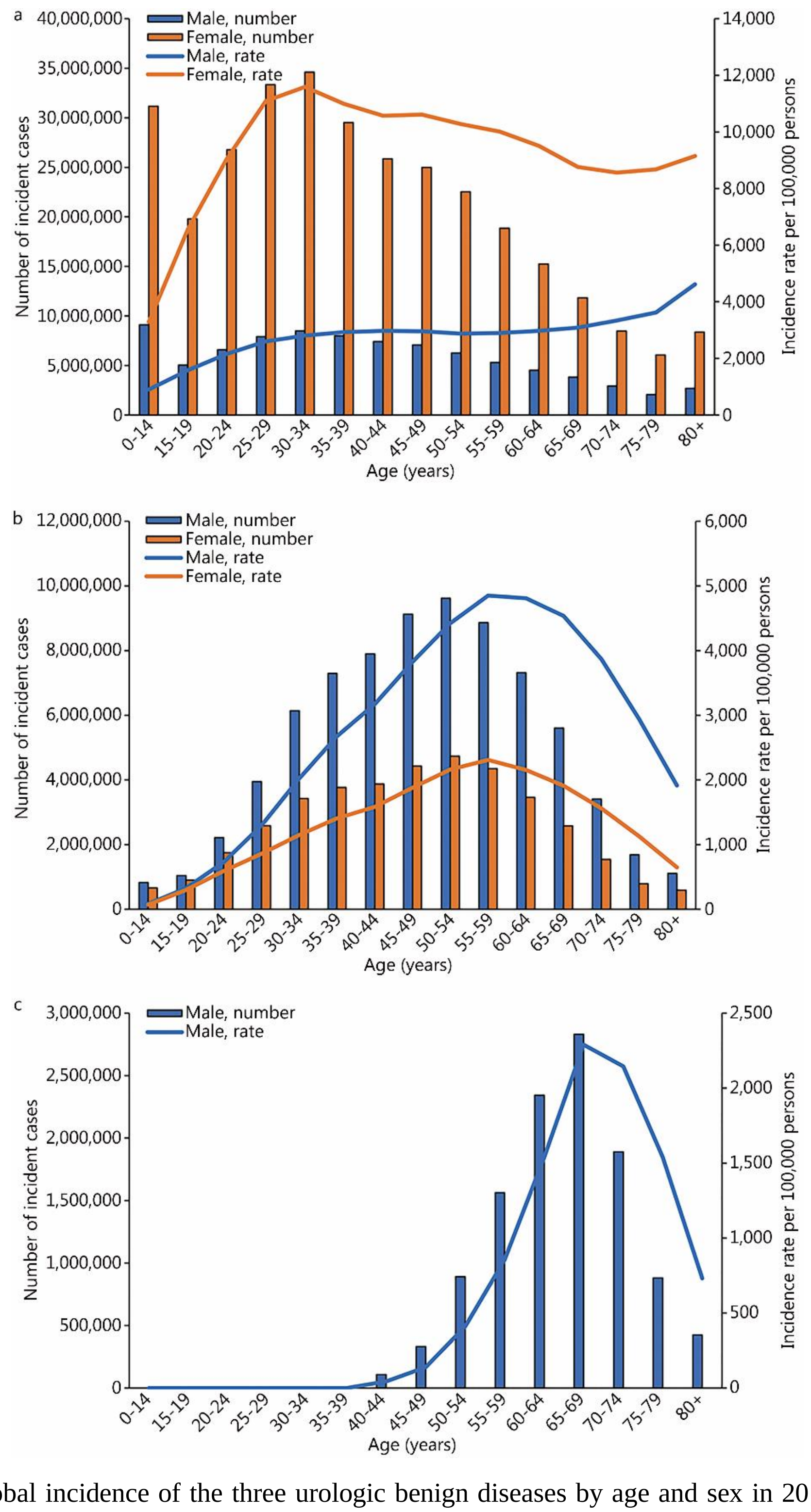

Fig. S15 Global incidence of the three urologic benign diseases by age and sex in 2019. **a** Urinary tract infections. **b** Urolithiasis. **c** Benign prostatic hyperplasia

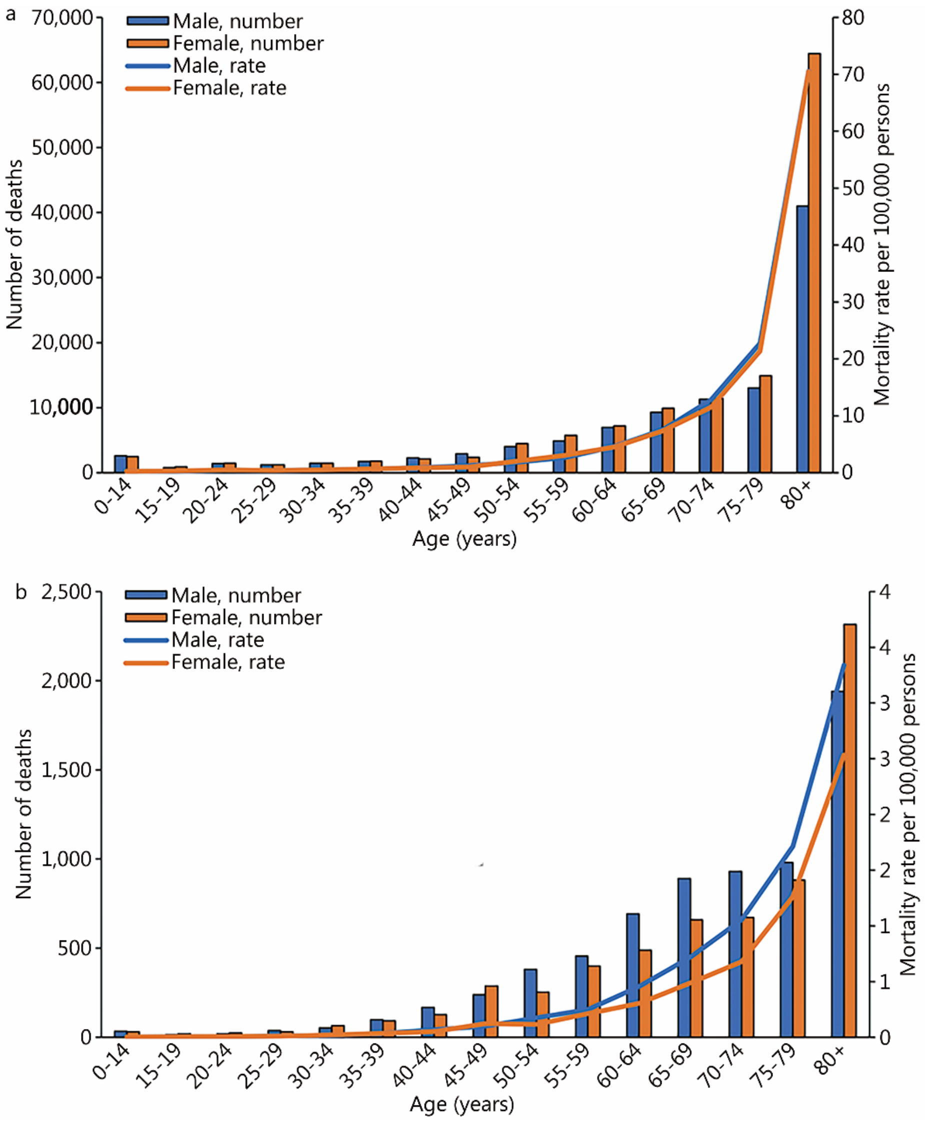

Fig. S16 Global mortality of urinary tract infections and urolithiasis by age and sex in 2019. **a** Urinary tract infections. **b** Urolithiasis

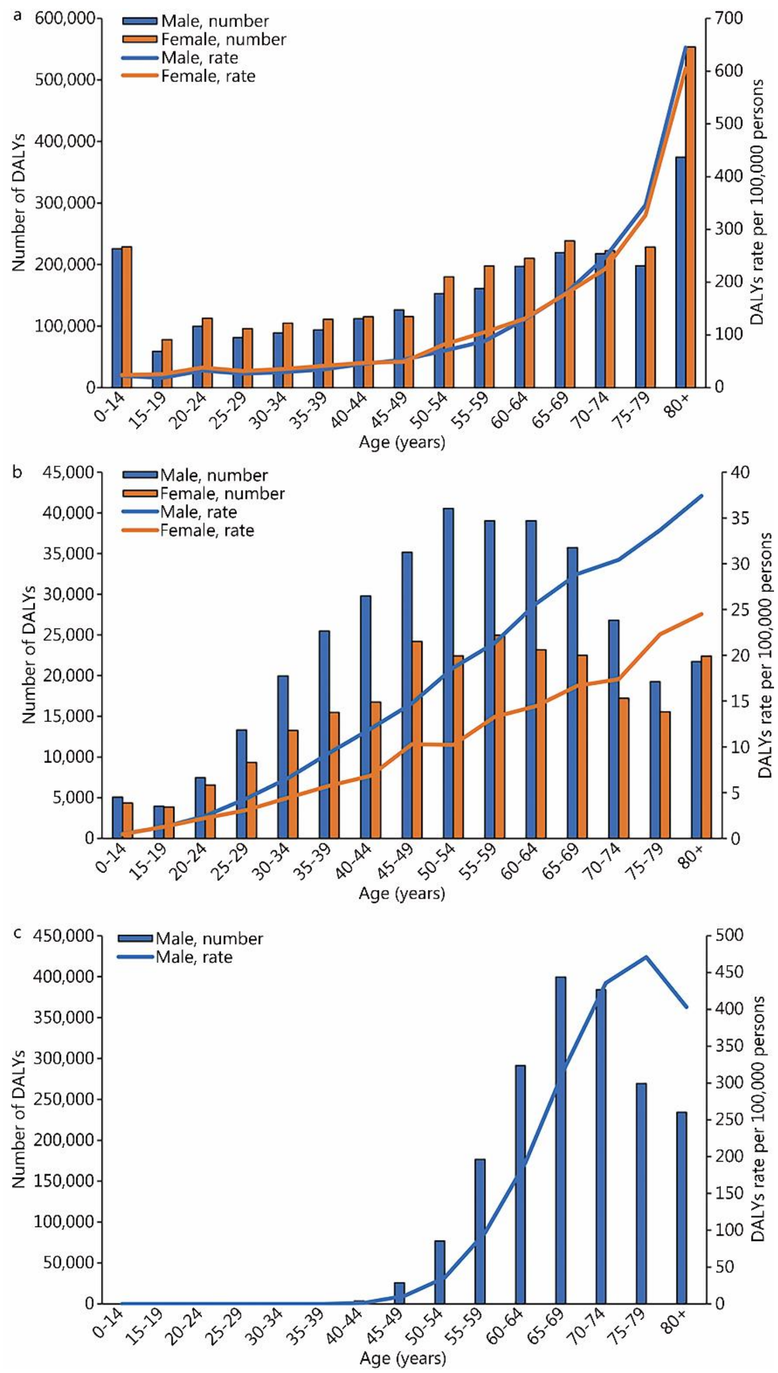

Fig. S17 Global DALYs of the three urologic benign diseases by age and sex in 2019. **a** Urinary tract infections. **b** Urolithiasis. **c** Benign prostatic hyperplasia
